# Exploring Linear *mono*-, *bis*- and *tris*-Acetylene Containing Agonists of the Human Olfactory Receptor OR1A1

**DOI:** 10.1021/acs.jmedchem.5c00282

**Published:** 2025-06-16

**Authors:** Weihong Liu, Luca S. Dobson, Chen Zhang, Jiahui Sun, Phillip T. Lowe, Yingjian Liu, Hanyi Zhuang, David O’Hagan

**Affiliations:** † School of Chemistry, 7486 University of St Andrews, North Haught, St Andrews KY16 9ST, U.K.; ‡ Intelligent Perception Lab, Hanwang Technology Co., Ltd, Beijing 100193, China

## Abstract

Structure–activity relationship studies on olfactory receptors such as OR1A1 enhance an understanding of the molecular mechanism of olfactory perception. Such receptors are considered to be important in regulating physiological roles beyond olfactory perception. Here a series of linearized ketones, alcohols, and a cyclic ether, extended between the oxygen functionality and a terminal *tert*-butyl group with either *mono*-, *bis*-, or *tris*-acetylene spacers, was prepared to explore the response of human olfactory receptor OR1A1. The best agonists were *bis*-acetylene rods combined with an aryl spacer, including the *bis*-acetylene ketones **13** and **14**, as well as the primary aryl-*bis*-acetylene alcohol **20** and the corresponding secondary alcohol **21**. In the latter case, there was a clear stimulatory preference for the (*R*)-**21** enantiomer. The experimental data were supported by molecular docking of the various ligands on the OR1A1 homology model. Further molecular dynamics simulations revealed atomic details in the OR1A1 binding pocket.

## Graphical abstract

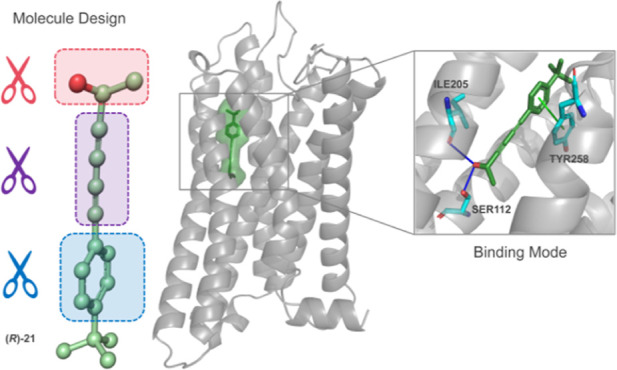

## Introduction

The human olfactory response is complex,^1,2^ but it is becoming clear that we possess a repertoire of around 400 olfactory receptors (OR) that together generate a combinatorial response to individual stimulatory molecules.^3–5^ ORs comprise the largest family of receptors under the G protein-coupled receptor (GPCR) superfamily. The identification of olfactory receptors at the genetic level and the overexpression of human olfactory receptors *in vitro* has allowed individual receptors to be explored in terms of their structure–activity relationships (SAR).^6^ In this context, the structure–activity profile of agonists of the human olfactory receptor OR1A1 is developing. The OR1A1 receptor is known to be activated by a range of compounds some illustrated in Figure 1. For example, muscone **1** and musk ketone **2** itself are very poor agonists.^7–9^ Across the commercial nitro musks,^10^ OR1A1 responds only to musk ambrette **3**, and to a lesser extent, musk tibetene **4** and musk xylene **5**, indicating a restricted level of response when compounds become more conformationally constrained.^10^ The best agonists are conformationally flexible aliphatic alcohols, aldehydes, and ketones converging around acyclic (mostly) C_10_-terpenoid structures such as octan-3-one **6**, citronellol **7**, and citronellal **8** (Figure 1).^7–9^ Most recently, the food register 3-methyl-2,4-nonanedione **9** ^11^ was identified as an extremely potent agonist and typifies the nature of the flexible structures that trigger OR1A1.

**Figure 1:**
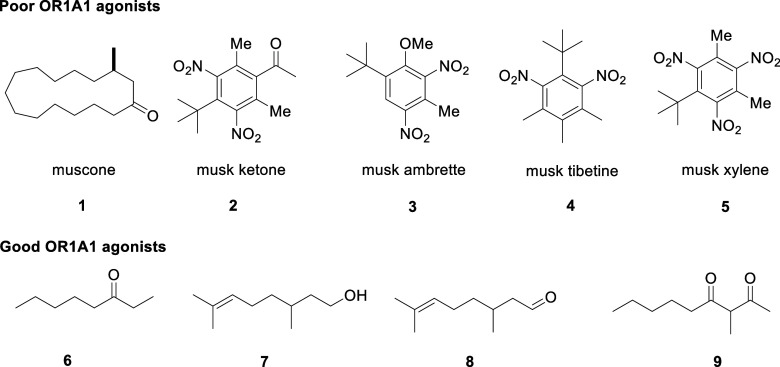
Poor **1–5** and good **6–9** agonists of the OR1A1 olfactory receptor.

The relationships between ORs and odorants continue to reveal unexpected complexities. Nonetheless, it is universally acknowledged that the structure of a protein profoundly influences its function. Previously, the conformation of OR1A1 has been primarily inferred through AlphaFold2 predictions or homology modeling.^8,12^ More recently, using a consensus protein design strategy, the cryo-EM structure of the consensus subfamily 1 human olfactory receptor, consOR1, was resolved.^13^ Furthermore, a combination of experimental techniques, such as amino acid mutations and computational methods including molecular docking and molecular dynamics simulations, have enhanced our ability to predict interaction sites between OR1A1 and various odorants. In 2004, Man et al. employed comparative analyses of orthologous and paralogous pairs of human and mouse olfactory receptors to identify 22 amino acid positions critical for odorant-binding, predominantly located on transmembrane helices 2 to 7 and the second extracellular loop.^14^ This work provided foundational insights into the molecular determinants of odor recognition within the GPCR family. Subsequently, Schmiedeberg et al. demonstrated that the amino acids ASN109^3×37^ and GLY108^3×36^ in OR1A1, along with their evolutionarily conserved counterparts in mouse ORs, play critical roles in binding and distinguishing enantiomers of small molecules like citronellol, primarily through direct interactions involving hydrogen bonds and steric effects.^8^ Furthermore, Geithe et al. identified the amino acids ASN109^3×37^, GLY108^3×36^, ILE205^5×46^, TYR251^6×44^, and TYR267^7×41^ in the enantioselective binding pocket of OR1A1 that facilitate distinct interactions with carvone enantiomers via hydrogen bonding, steric, and hydrophobic interactions.^6^ These studies have enhanced our understanding of the binding patterns between OR1A1 and ligands.

It is now widely accepted that ORs have important roles beyond detecting odorants in olfaction.^15^ These ectopic ORs are distributed throughout the human body and may indicate physiological roles comparable to non-olfactory GPCRs. Previous studies have shown that OR1A1, being expressed in the liver, may be involved in the regulation of glucose and lipid metabolism homeostasis. For example, OR1A1 may function as a surface receptor in hepatocytes that regulates triglyceride synthesis via the PKA-CREB-HES-1 signaling axis.^16^ In another study, it was shown that monoterpene ligands of OR1A1 ameliorated hepatic lipid deposition through the AMPK/SREBP-1/FASN pathway.^17^ It is also known that the mouse homologue of OR1A1, OLFR43, is highly expressed in intestinal L cells and is responsible for stimulating insulin secretion.^18^ These findings support the role of OR1A1 as a potential drug target and the discovery of novel OR1A1 ligands warrants potential pharmacological applications.

In this paper, we report our work on mapping SAR for OR1A1. Molecules were prepared to probe the spatial and steric constraints for agonist activity. The project placed a particular focus on identifying progressively longer structures with very limited conformational flexibility. The design approach taken used the acetylene group as a spacer and this resulted in the preparation of compounds **10–22** as illustrated in Figure 2. These compounds have either one, two or three acetylenes in the central chain, introduced to generate longitudinal compounds with a minimal steric and limited conformational impact beyond extending their length.

**Figure 2:**
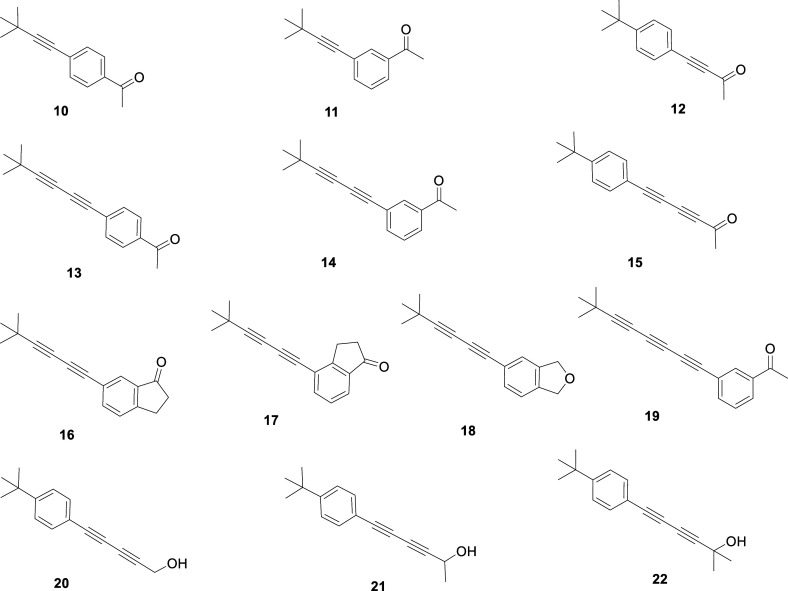
Linearized probes used to explore minimum agonist requirements of the human OR1A1 olfactory receptor.

## Results and Discussion

In overview, the *mono*-acetylene ketones **10–12** did not show significant activities as agonists of the OR1A1 receptor and were clearly too short to have an impact. The study extended to the *bis*-acetylene ketones **13–18**, which were used to increase the molecular length. As a class, the *bis*-acetylenes showed good agonist activity; however, the response was sensitive to regiochemical ordering of substituents favoring *meta* over *para* isomers. A further extension in length to the *meta*, *tris*-acetylene acetophenone **19** did not result in an active compound, suggesting the molecular length was now too long. It was further noted that free alcohols in the *bis*-acetylene series as in **20–22** offered potent agonist activity and the most potent agonist was the (*R*)-**21** enantiomer of this secondary alcohol. Molecular docking and molecular dynamics simulations corroborated the experimental results.

### Synthesis and Olfactory Receptor (OR1A1) Assays

The *mono*-acetylene ketones **10–12** were synthesized via Sonogashira cross-coupling reactions^19,20^ of iodoacetophenones **23** and **24** with *tert*-butyl acetylene **25** as illustrated in Scheme 1. These reactions proceeded smoothly, and *para* **10** and *meta* **11** acetophenones were isolated in modest to good yields (68% and 63%, respectively).

**Scheme 1:**
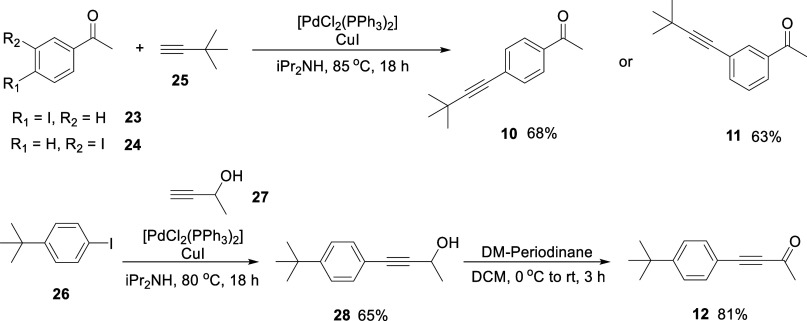
Synthesis of *mono*-Acetylenes **10–12**

Monoacetylenes **10–12** were assayed against the OR1A1 receptor; however, they showed agonist activity only at very high concentrations and it was clear that shorter molecules with a single acetylene spacer were not sufficient to trigger a response (Supporting Information Figure S4a). The synthesis effort then focused on *bis*-acetylene ketones **13–18**. The *bis*-acetylene ketones **13** and **14** were synthesized in a four-step sequence as illustrated in Scheme 2. A key approach to their assembly was the deployment of the Cadiot–Chodkiewicz cross-coupling reaction^21,22^ which used bromoacetylenes **34** and **35** with *tert*-butyl acetylene **25** to generate the desired *bis*-acetylene connectivity in **13** and **14**. *bis*-Acetylene ketone **15** was also identified as a target and the three-step route outlined in Scheme 3 was developed. Again a Cadiot–Chodkiewicz cross-coupling reaction^21,22^ this time between bromopropargyl alcohol **36** and 4-(*tert*-butyl)­phenylacetylene **37**, generated the free propargylic alcohol **21** in good yield (73%). This was then oxidized to ketone **15** using the Dess–Martin periodane reagent.^23,24^

**Scheme 2:**
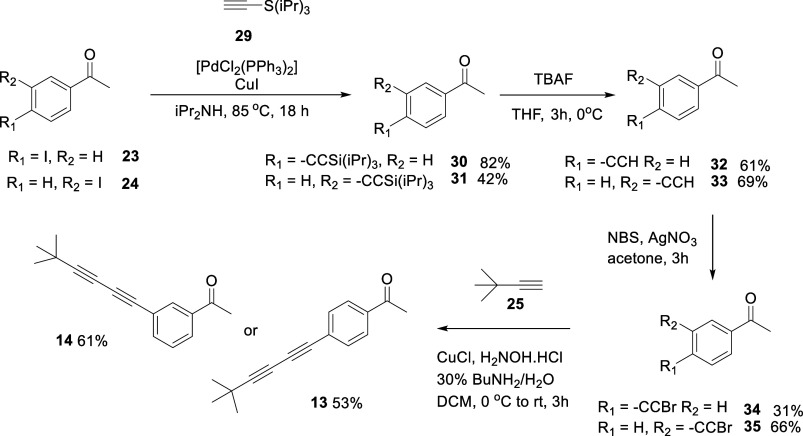
Synthesis of *bis*-Acetylene Ketones **13** and **14**

**Scheme 3:**
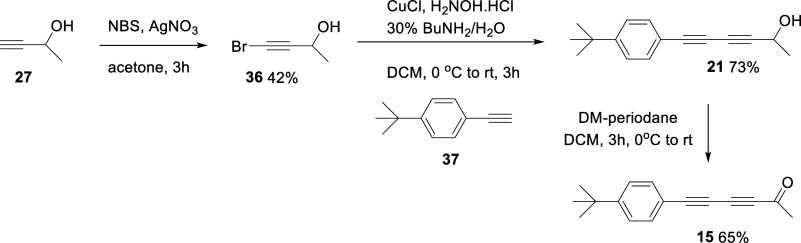
Synthesis of *bis*-Acetylene Ketone **15**

Ketones **13**, **14** and **15** were tested on OR1A1.^7,25,26^ We found that the acetophenones **13** and **14** activated OR1A1 while the acetylenenic ketone **15** was inactive. Notably, between the acetophenones **13** and **14**, there was increased efficacy for the *meta*-aryl geometry in **14**. Since the *bis*-acetylene ketone **15** was inactive toward OR1A1, the possibility of whether it could be an antagonist for OR1A1 was explored. While the concentration of the positive control, carvone, was held constant, increasing the concentration of **15** did not result in a decrease in receptor activity, indicating that **15** was not an antagonist (Supporting Information Figure S5) (Figure 3).

**Figure 3:**
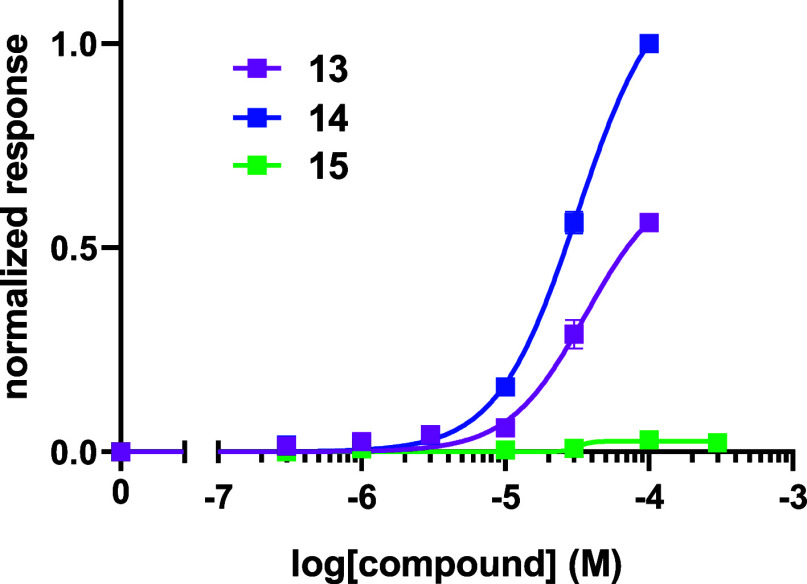
Normalized responses of synthesized *bis*-acetylenes **13–15** with the OR1A1 receptor. The responses are normalized to the maximum response to compound **14**.

In order to explore extending the acetylene spacers further, a synthesis of the *tris*-acetylene **19** with *meta* substitution was conducted as illustrated in Scheme 4. The familiar synthesis protocols were adopted but in this case assembly of **19** involved a Sonogashira cross-coupling followed by two sequential Cadiot–Chodkiewicz reactions. In the event *tris*-acetylene **19** proved to be a weak agonist and had a similar response to **10** and **11** in assays with the OR1A1 receptor (Supporting Information Figure S4b). Clearly, increasing the linker from a two- to a three-acetylene spacer unit had a detrimental effect, and the two-acetylene spacer emerged as optimal in this study.

**Scheme 4:**
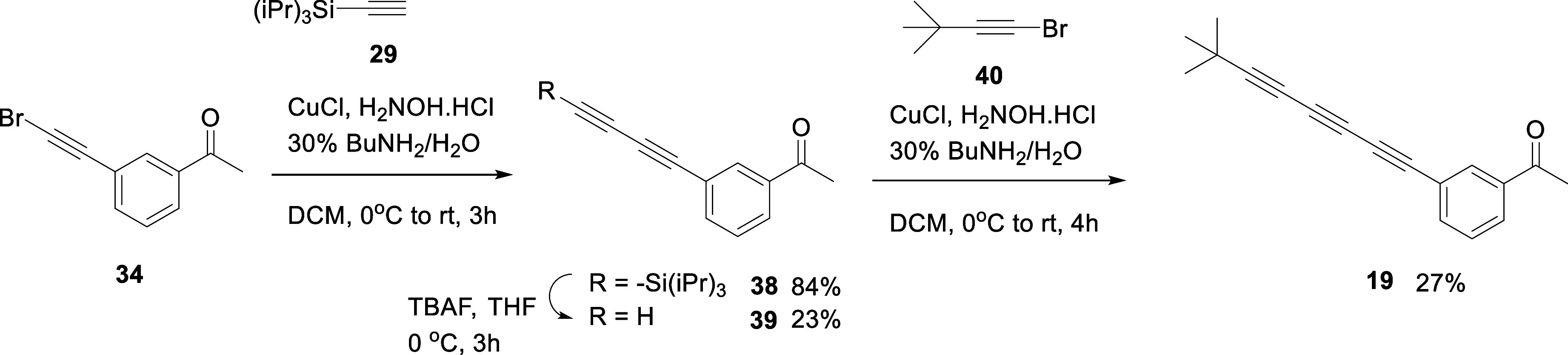
Synthesis of *tris*-Acetylene Ketone **19**

In acetophenones **13** and **14**, the aryl to acyl bond is the only rotatable bond that influences conformation. Given that the *meta*-aryl isomer **14** was the more efficacious, in the next phase, the fully conformationally constrained indanones **16** and **17** were prepared. These constructs physically lock the carbonyl group into a fixed orientation but maintain a *meta* geometry, and these were chosen to be the appropriate probes to explore the preferred orientation of the ketone carbonyl when binding to the receptor. The selected route for the preparation of indanones **16** and **17** is illustrated in Scheme 5. The routes progressed through similar steps to the previous assemblies but started from the bromoindanone isomers **41** and **42**. A sequence of Sonogashira reactions with silyl protected acetylene **29**, and then silyl deprotection followed by terminal bromination of the acetylene generated bromoacetylenes **47** and **48**. This then allowed Cadiot–Chodkiewicz cross-coupling reactions to generate **16** and **17**.

**Scheme 5:**
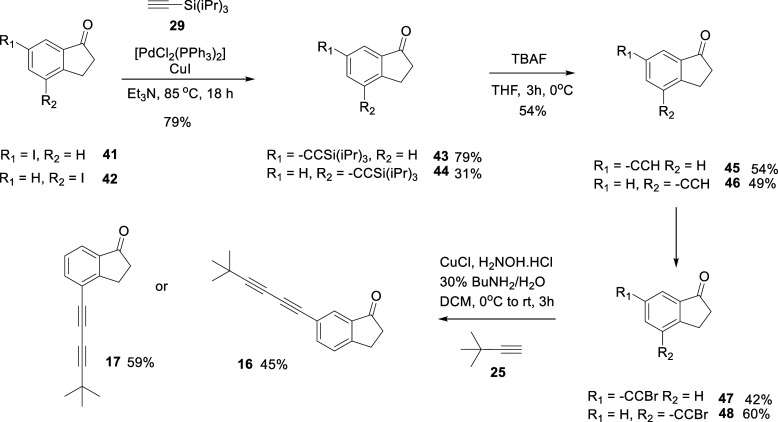
Synthetic Route to Indanones **16** and **17**

The assay outcomes of the indanone isomers **16** and **17** are summarized in Figure 4. Indanone **16** was not active whereas **17** induced micromolar range potency with modest to good agonist efficacy. Even for indanone **17**, the response suggests that the indanone shape is less optimal than the linear *bis*-acetylene **14** which displayed better potency when triggering a response on the olfactory receptor (Table 1). Nonetheless, when comparing **16** and **17**, the orientation of the carbonyl group emerges as highly influential in determining the strength of interaction with the receptor, although the aliphatic ring carbons may compromise agonist activity overall, relative to the linear probes.

**Figure 4:**
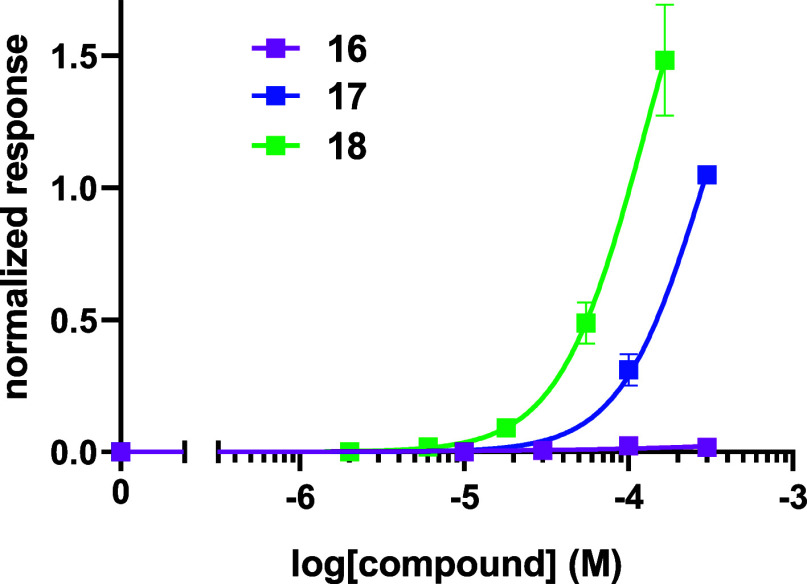
Normalized responses of indanone *bis*-acetylenes **16** and **17**, and dihydroisobenzylfuran *bis*-acetylene **18** with OR1A1 receptor. The responses are normalized to the maximum response of OR1A1 to compound **21** (not shown).

**Table 1:** OR1A1 Responses and Binding Energy of Ligands[Table-fn t1fna]

ligands	log EC_50_	binding energy (kcal/mol)
**13**	–4.44 ± 0.28	–36.34 ± 0.79
**14**	–4.45 ± 0.08	–36.81 ± 0.50
**17**	N/A	–35.95 ± 1.41
**18**	N/A	–35.83 ± 1.49
**20**	–4.37 ± 0.15	–35.90 ± 1.23
**21**	–4.36 ± 0.27	
(*R*)-**21**	–5.13 ± 1.36	–39.34 ± 1.25
(*S*)-**21**	–4.62 ± 0.08	–38.09 ± 0.83
**22**	N/A	–35.07 ± 1.35

a The binding energy, calculated using the MMGBSA method, was determined from the mean values and standard deviations derived from three independent molecular dynamics simulations. The EC_50_ values represent the concentration of the ligand required to achieve a half-maximal response. The best-fit values are shown with 95% confident interval. “N/A” means the data could not be fitted to a curve to give a meaningful EC_50_. The ligands **10**, **11**, **12**, **15**, **16**, and **19** did not elicit a measurable response in the experiment and are not shown.

The combination of SAR outlined above led to identification of dihydroisobenzylfuran *bis*-acetylene **18** as a synthetic tagget. This fused aryl heterocycle retains a *bis*-acetylene spacer with the favored *meta* geometry. The position of the heterocyclic oxygen was designed such that it adopts a location on the rigid framework that approximates that of the carbonyl of the active indanone **17**. This structure was therefore prepared to further probe the regiospecific mapping of the oxygen atom. The synthetic route to **18** is outlined in Scheme 6.

**Scheme 6:**
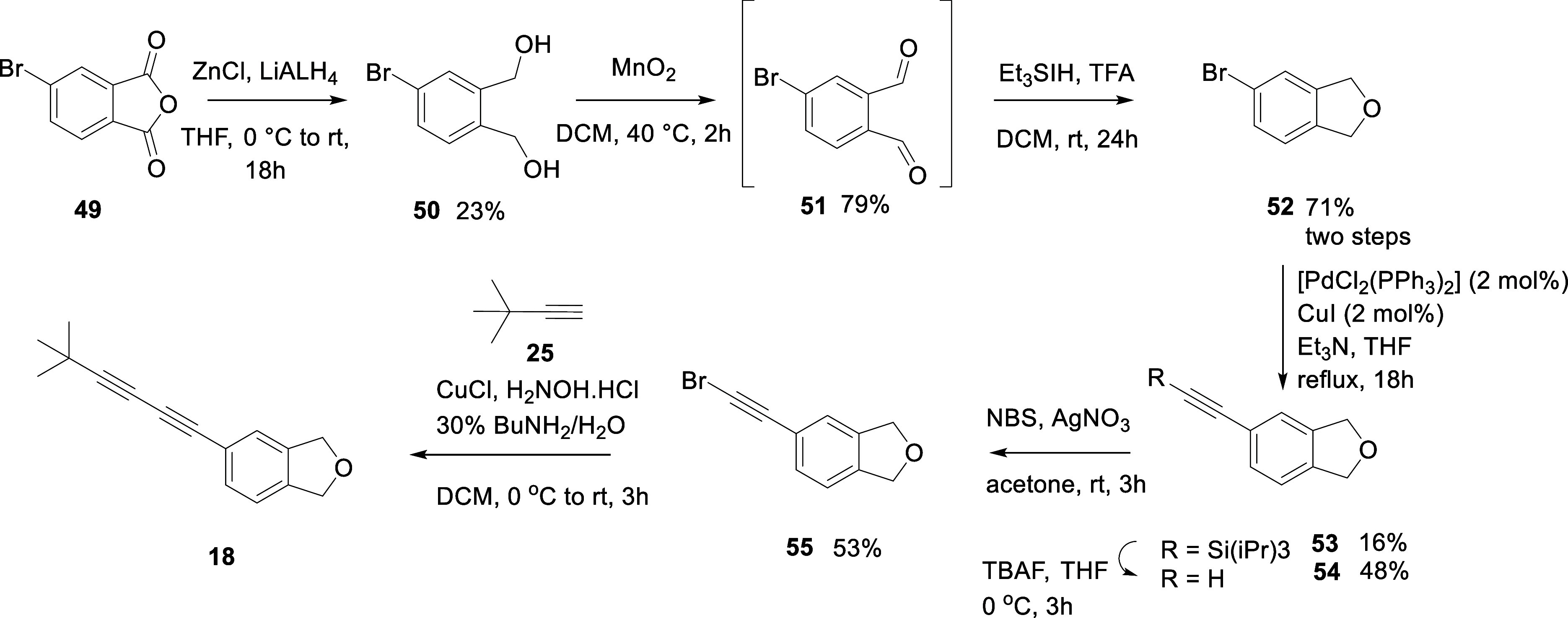
Synthesis of Dihydroisobenzylfuran *bis*-Acetylene **18**

The binding energy, calculated using the MMGBSA method, was determined from the mean values and standard deviations derived from three independent molecular dynamics simulations. The EC_50_ values represent the concentration of the ligandodorant required to achieve a half-maximal response. The best-fit values are shown with 95% confident interval. “N/A” means the data could not be fitted to a curve to give a meaningful EC_50_. The ligandodorants **10**, **11**, **12**, **15**, **16**, and **19** did not elicit a measurable response in the experiment and were not shown.

The combination of SAR outlined above led to identification of dihydroisobenzylfuran *bis*-acetylene **18** as a synthetic tagrget. This fused aryl heterocycle retains a *bis*-acetylene spacer with the favored *meta* geometry. The position of the heterocyclic oxygen was designed such that it adopts a location on the rigid framework that approximates that of the carbonyl of the active indanone **17**. This structure was therefore prepared to further probe the regiospecific mapping of the oxygen atom. The synthetic route to **18** is outlined in Scheme 6.

The synthesis started with the reduction of cyclic anhydride **49** with LiAlH_4_ and zinc chloride to generate diol **50** and then cyclization to form the dihydroisobenzylfuran **52**. This cyclization was achieved in a telescoped reaction sequence where oxidation of diol **50**, with MnO_2_ ^27^ gave dialdehyde **51**. Dialdehyde **51** was directly cyclized without isolation, after the addition of triethylsilane and trifluoroacetic acid.^28^ This two-step protocol proved to be relatively efficient. Bromoaryl-benzofuran **52** was progressed first by cross-coupling to generate the silyl protected acetylene **53** and then desilylation gave the free acetylene **54** which was then brominated^29^ to generate **55**, necessary for a final Cadiot–Chodkiewicz reaction with *tert*-butylacetylene **25**. This allowed access to the *bis*-acetylene target **18**. When assayed against OR1A1 and compared with indanones **16** and **17**, *bis*-acetylene **18** displayed a stronger response (Figure 4 and Table 1).

The study now explored the agonist activity of linearized *bis*-acetylene primary, secondary and tertiary alcohols **20–22**. The route to secondary alcohol **21** is already described in Scheme 3 and it displayed a much stronger interaction with the OR1A1 receptor than the corresponding ketone **15**, which was essentially inactive. Thus, the hydrogen bonding donor capacity of alcohol **21** has a significant effect on agonist activity. In order to explore this further, the corresponding primary **20** and tertiary **22** alcohols were prepared by adding or subtracting a terminal methyl group. Alcohols **20** and **22** were accessed by the route summarized in Scheme 7. Accordingly, bromopropargyl alcohols **58** and **59** were prepared for independent cross-coupling reactions with *tert*-butyl-phenylacetylene **37** and this gave the desired alcohols **20** and **22**, respectively.

**Scheme 7:**
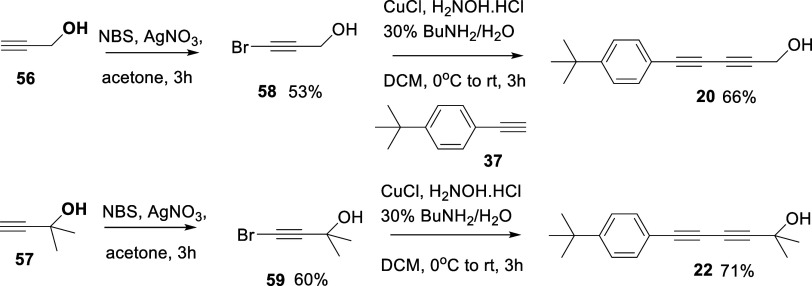
Synthesis Routes to the Primary and Tertiary *bis*-Acetylene Alcohols **20** and **22**

The three alcohols **20–22** were all active agonists of the OR1A1 receptor and the primary alcohol **20** and secondary alcohol **21** elicited a similar level of response on the receptor. Since **21** contains a stereogenic center, it was presented to the assay as a racemate. The assay outcomes are summarized in Figure 5. Citronellol **7** is already reported to be a good agonist of the OR1A1 receptor although notably the enantiomers of citronellol, (*R*)-**7** and (*S*)-**7**, with EC_50_ values of 82.5 μM ± 6.3 and 92.6 μM ± 10.2, respectively, are not significantly distinguished by the receptor.^8^ It became of interest therefore to consider the individual enantiomers of **21** to determine if the alcohol moiety directly attached at the stereogenic center has a more discriminating effect as might be expected. Accordingly, the enantiomers were prepared independently from either (*R*)-**27** and (*S*)-**27** propargyl alcohols following the protocols summarized in Scheme 3.

**Figure 5:**
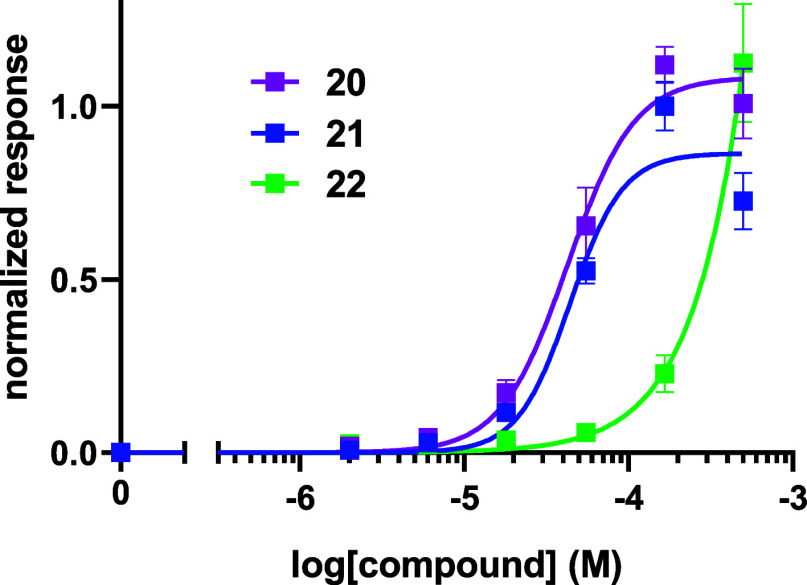
Normalized responses of alcohols **20**, **21** (racemate), and **22**, with the OR1A1 receptor. The responses are normalized to the maximum response to compound **21**.

The assays summarized in Figure 6 show a clear difference in agonist activity between the enantiomers with the (*R*)-**21** enantiomer showing the strongest agonist effect (Table 1). Given that tertiary alcohol **22** was the weakest agonist in this alcohol series and will find it most challenging to donate its hydrogen due to steric occlusion, the outcomes are consistent with previous studies, which have recognized the importance a hydrogen bonding donor role for long chain aliphatic alcohols.^7–9^ Notably, primary alcohol **20** also has strong agonist activity, indicating that it too can make a good hydrogen bond, consistent with a lack of steric occlusion.

**Figure 6:**
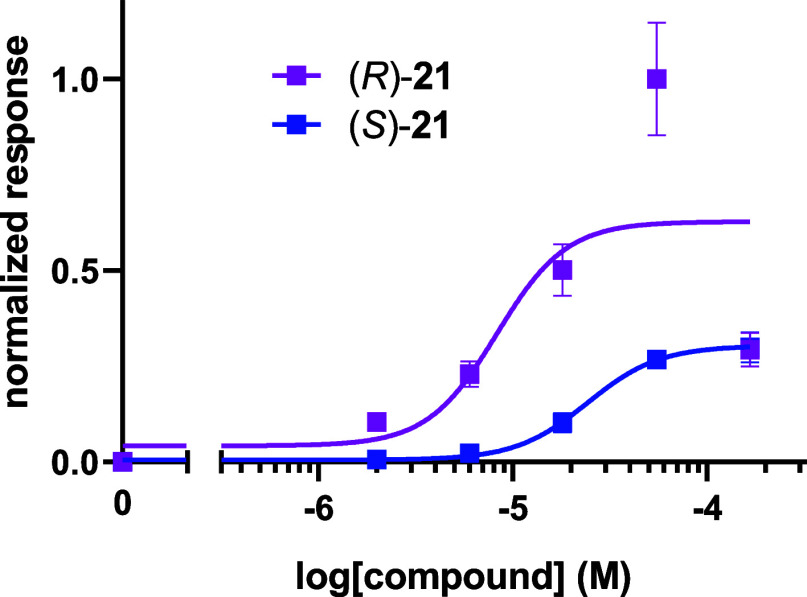
Normalized responses of (*R*)-**21** and (*S*)-**21** with the OR1A1 receptor. The responses are normalized to the maximum response to compound (*R*)-**21**.

To better understand the SAR between OR1A1 and the array of acetylenes, we used homology modeling and molecular docking to delineate the binding pocket of OR1A1. The structure of OR1A1 was derived based on the structure of the most recently resolved consensus OR1,^13^ and the quality of the modeled structure was subsequently assessed using the Ramachandran plot (Supporting Information Figure S6). This assessment confirmed the method’s feasibility, indicating that most residues adopted reasonable conformations.

The binding energies of compounds that elicited OR1A1 responses were derived from MD simulations, and their EC_50_ values are shown (Table 1). MD simulations were conducted using Gromacs for receptor–agonist complexes. These complex systems underwent three 150 ns MD simulations, starting from the docked conformations with the highest score. The results of these simulations provided insights into the binding free energy, interaction sites, and the stability of the binding modes. Throughout the 150 ns period, the RMSD values of the receptor stabilized within a consistent range across the three parallel simulations for each agonist, demonstrating the robustness of the data (Supporting Information Figure S7). The average of the binding free energies obtained from three parallel MD simulations was used for comparison with log EC_50_. Among the tested ligands, compound **18** showed a relatively low binding affinity in both experimental and computational assessments, with no available log EC_50_ value and a binding energy of −35.83 kcal/mol. By contrast, (*R*)-**21**, with a log EC_50_ of −5.128 and the lowest binding energy, exhibited the strongest binding affinity. Furthermore, the different experimental results and binding energies of the two enantiomers (*S*)-**21** and (*R*)-**21** also made us aware of the importance of molecular chirality in the interaction of these agonists with ORs. Overall, there was a relatively strong correlation between the log EC_50_ and binding energy, with a correlation coefficient of 0.95 (*p* < 0.05, excluding data with a log EC_50_ value of N/A).

The binding energy, calculated using the MMGBSA method, was determined from the mean values and standard deviations derived from three independent molecular dynamics simulations. The EC_50_ values represent the concentration of the ligand required to achieve a half-maximal response. The best-fit values are shown with 95% confident interval. “N/A” means the data could not be fitted to a curve to give a meaningful EC_50_. The ligands **10**, **11**, **12**, **15**, **16**, and **19** did not elicit a measurable response in the experiment and were not shown.

The binding sites of OR1A1 with ligands were analyzed using molecular docking. A global view revealed the precise positioning of (*R*)-**21** within the receptor’s binding pocket, located between TM3, TM4, TM5 and TM6 (Figure 7a). Other ligands also showed similar binding positions. Specific details of the binding pocket (Figure 7b) revealed interactions within a distance of 4 Å of (*R*)-**21**. The alcohol is stabilized by hydrogen bonds formed with residues SER112^3×40^ and ILE205^5×46^, as well as π–π stacking with TYR258^6×55^. The binding sites were compared with the conserved residues highlighted based on sequence homology analysis by Man et al.^14^ and 12 common residues were identified, including SER112^3×40^ and ILE205^5×46^ (Figure 7c). These residues were also found to play important roles in the binding of other ligands to OR1A1 in the previous study.^6–8,13^ Notably, close-up views of the OR1A1 binding sites for (*R*)-**21**, (*S*)-**21**, **13**, and **18** are provided (Figure 7d). Both (*R*)-**21** and (*S*)-**21** can form a hydrogen bond with the hydroxyl group of SER112^3×40^ and π–π stacking with TYR258^6×55^, but only (*R*)-**21** is capable of also forming a hydrogen bond to the carbonyl oxygen of ILE205^5×46^. TYR258^6×55^, located on TM6, interacts with ligands through π–π stacking. Since the oscillation of TM6 is a key indicator of GPCR activation and directly affects downstream signaling,^30,31^ TYR258^6×55^ may play a critical role in modulating receptor function. Compound **13** forms a hydrogen bond with SER112^3×40^. As well as π–π stacking with TYR258^6×55^, compound **18** forms a hydrogen bond with ASN109^3×37^. ASN109^3×37^ is also a key site of action reported in previous studies.^6,8^ Similar binding modes were observed in the assessment of other of the agonists (Supporting Information Figure S8). Compounds **14** and **17** exhibited binding patterns akin to compounds **18** and **13**, respectively. Compound **20** showed similarities to (*R*)-**21**, and compound **22**, due to steric occlusion, forms a hydrogen bond with THR182 in the extracellular loop 2 region, in addition to π–π stacking with TYR258^6×55^.

**Figure 7:**
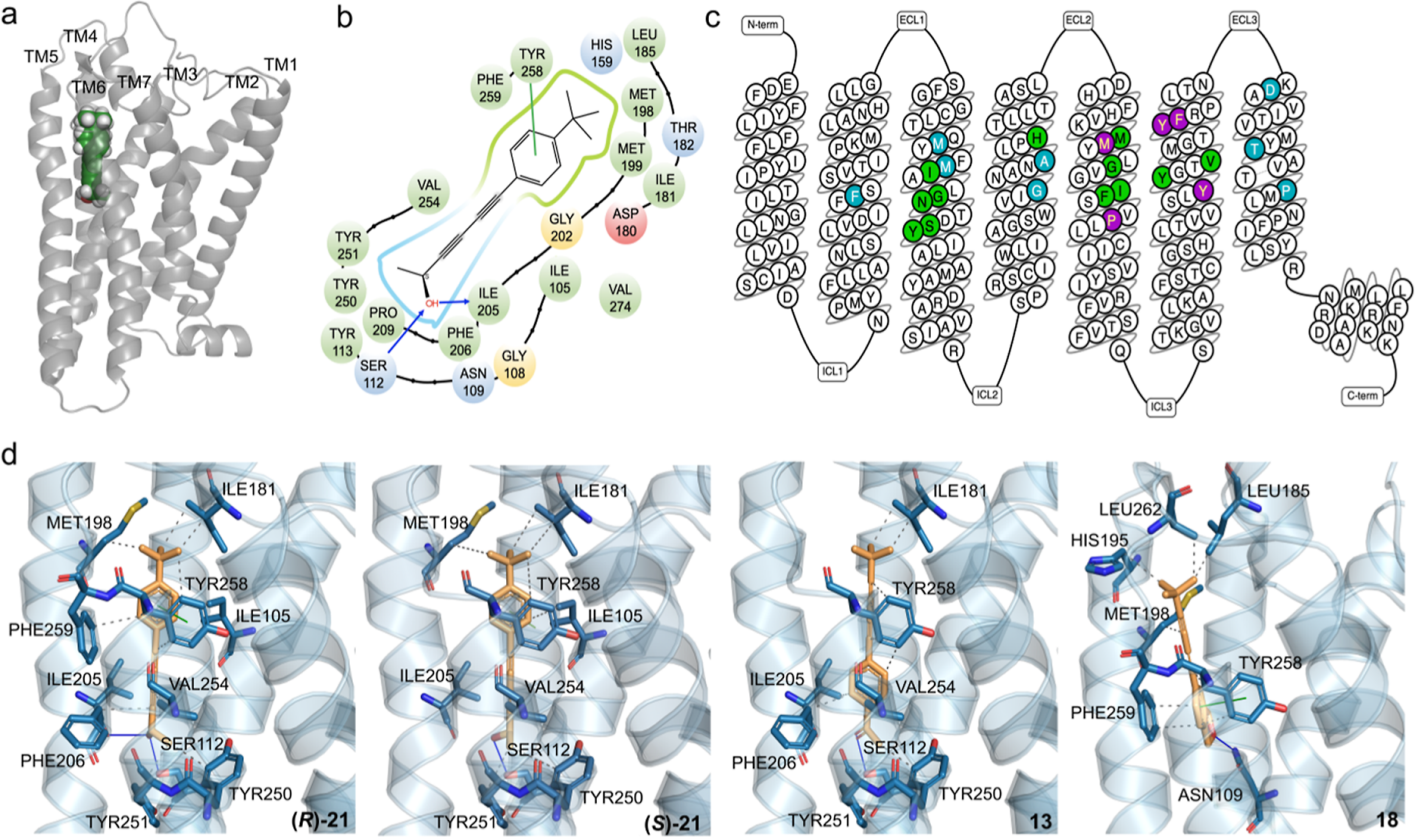
Structural analysis of OR1A1 binding sites. (a) Global view of OR1A1 which was homology modeled from the experimental structure (PDB ID: 8UXY) bound with (*R*)-**21**. (b) The binding pocket of OR1A1 and (*R*)-**21** (within 4 Å). Blue arrows indicate hydrogen bonds. The residues are color-coded by hydrophobicity and electrophilicity. (c) Snakeplot of OR1A1 highlighting the binding pocket of (*R*)-**21**. Green residues are consistent with previous studies by Man et al.,^14^ while purple and blue residues are unique binding sites identified in this study and in Man et al.,^14^ respectively. (d) Close-up views of the key interactions between OR1A1 and four compounds: (*R*)-**21**, (*S*)-**21**, **13** and **18**, where gray dashed lines represent hydrophobic interactions, green solid lines represent π–π stacking, and blue solid lines represent hydrogen bonds.

The MMGBSA free energy decomposition of residues in the binding pocket of (*R*)-**21** is presented as a histogram. Residues with a binding free energy lower than −1.0 kcal/mol, including ILE205^5×46^, PHE206^5×47^, and ILE105^3×33^, play a significant role in maintaining the binding between the protein and compound (*R*)-**21**. Additionally, although the binding free energies of TYR251^6×48^ (−0.95 kcal/mol), ASN109^3×37^ (−0.44 kcal/mol), and SER112^3×40^ (−0.43 kcal/mol) are slightly higher, they also have non-negligible significance in the overall interaction network. These interactions reflect the complexity and robustness of ligand binding, providing multiple anchoring points that enhance the stability and affinity of the receptor–agonist complex.^32^ A contact frequency heatmap of OR1A1 binding site residues versus agonist atoms identified ASN109^3×37^, SER112^3×40^, GLY108^3×36^, ILE205^5×46^, and TYR258^6×55^ as high-frequency contact residues, consistent with free energy calculations (Figure 8b). Specifically, the hydroxyl group showed preferential interactions with GLY108^3×36^, SER112^3×40^, and ILE205^5×46^, while the aryl ring primarily contacted TYR258^6×55^ (via π–π stacking) and GLY202^5×43^ (through hydrophobic interactions).

**Figure 8:**
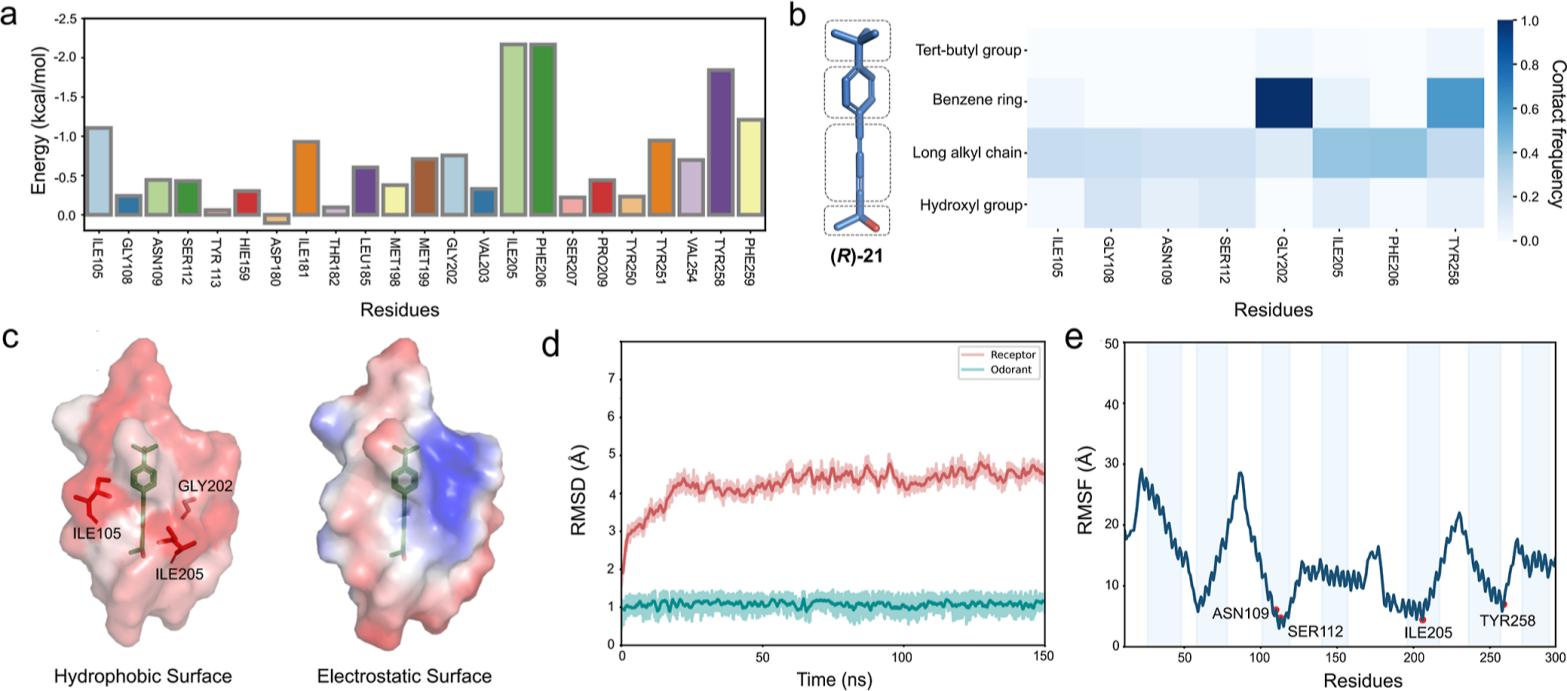
Analysis of the stability and dynamics of receptor–ligand complexes. (a) MMGBSA free energy decomposition for residues in the binding pocket of compound (*R*)-**21** (within 6 Å). The bar graph illustrates the contribution of specific residues to the binding free energy. (b) Heatmap of contact frequencies between OR1A1 binding site residues and (*R*)-**21**. (c) The hydrophobic and electrostatic surface of the OR1A1 binding pocket (within 4 Å). (d) RMSD plot from MD simulations, calculated from the average of three parallel simulations, showing the stability of the receptor–ligand complex over time for compound (*R*)-**21**. The RMSD of the receptor (red) and the ligand (cyan) are indicated. (e) RMSF plot from MD simulations for compound (*R*)-**21**, calculated from the average of three parallel simulations, highlighting the flexibility of different transmembrane helices. Key residues ASN109^3×37^, SER112^3×40^, ILE205^5×46^, and TYR258^6×55^ are indicated.

The hydrophobic and electrostatic properties of the binding pocket were also emphasized (Figure 8c). Hydrophobic residues ILE105^3×33^, GLY202^5×43^, and ILE205^5×46^ coordinated to form a stabilizing microenvironment for ligand accommodation through nonpolar interactions within the binding pocket. Meanwhile, positively charged residues are distributed around the hydroxyl group of (*R*)-**21**, increasing stability. The feasibility of the combined model was further confirmed by the RMSD and RMSF plots obtained from the MD simulations. The RMSD plot indicated stability of the complex of (*R*)-**21** and OR1A1 over time while the RMSF plot highlighted the flexibility of different residues (Figure 8d,e). The low RMSF values of key residues were shown, including ASN109^3×37^, SER112^3×40^, ILE205^5×46^, and TYR258^6×55^, which is also consistent with previous findings.

## Conclusion

In summary, the synthesis of a novel class of OR1A1 agonists with acetylene spacers has been detailed. These were designed to offer linearity but with minimal conformational flexibility and they represent a very different structural profile in that respect to agonists of the OR1A1 receptor to date. The aryl *bis*-acetylene constructs were the most active agonists relative to related *mono*- and *tris*-acetylene constructs, and we find that the most potent agonists of this class have terminal alcohol or ketone groups. A comparison of the enantiomers of secondary alcohol **21** revealed a clear preference for (*R*)-**21**. Results of molecular docking and MD simulations are congruent with the pharmacological findings. Our analysis of the binding modes of OR1A1 with a range of agonists should be instructive for the design of subsequent small molecule agonists.

The discovery of novel ligands that modulate OR1A1 activity with high potency and selectivity reveals details of ligand–receptor interactions and may shed light on the development of new pharmacological agents targeting physiologically relevant ORs.

## Experimental Section

### Synthetic Methods

Methods for the synthesis and then analysis of compounds **10–22** are described in the Supporting Information. These compounds were prepared ≥95% purity as determined by NMR or HPLC and (*R*)-**21** and (*S*)-**21** were prepared in ≥98% ee as determined by chiral HPLC.

### Cell Culture and Luciferase Assay

Olfactory receptor activation was evaluated using the Dual-Glo Luciferase Assay System (Promega (Beijing) Biotech Co., Ltd., Beijing, China). HEK293T-derived cells were maintained in minimal essential medium (Sigma) containing 10% fetal bovine serum (Sigma) (M10), 500 g/mL penicillin–streptomycin (Invitrogen), and 6 g/mL amphotericin B (Sigma) in a 37 °C incubator with 5% CO_2_. After culturing to a desired confluence, the cells were plated on 96-well plates (Corning Inc., Kennebunk, ME, USA). For each 96-well plate, 1 μg of CRE-luciferase, 0.5 μg of pRL-SV40, 0.5 μg of OR, 0.5 μg of RTP1S, and 0.25 μg of M3R were transfected using the transfection reagent Lipofectamine2000 (Invitrogen, Life Technologies Corp., Carlsbad, CA, USA). Twenty-four hours post transfection, the transfection media were removed, and the cells were rinsed with 50 μL PBS buffer (pH 7.4) per well. The cells were then stimulated with 25 μL 1% (v/v) ligand samples diluted in CD293 stimulation medium (Gibco Brand, ThermoFisher Biochemical Products (Beijing) Co., Ltd., Beijing, China). Four hours post stimulation, firefly luciferase (FL) and Renilla luciferase (RL) activities were measured following manufacturer’s protocols, using a Synergy H1 plate reader (BioTek Instruments, Winooski, VT, USA). Normalized luciferase activity was calculated using the formula: FL/RLX-FL/RLXC, where FL/RLX is the response to the ligands, FL/RLXC is the basal value of the same OR. Each experiment was repeated at least 3 times and error bars indicate standard error mean. Data were analyzed with GraphPad Prism.

### Homology Modeling

The structure of OR1A1 was derived by homology modeling with Swiss-model,^33^ using the high-resolution cryo-EM structure of consOR1 (PDB ID: 8UXY) as a template. The model was then refined by energy minimization using Gromacs.^34^ The structure’s quality was assessed by the Ramachandran plot.^35,36^

### Molecular Docking

Molecular docking was performed using AutoDock Vina.^37^ The structures of both the receptor and ligands were prepared by adding hydrogen atoms and assigning Gasteiger charges. The receptor was optimized using the OPLS_2005 force field and ligand conformations were refined and generated via the Python package Meeko.^38^ Residues within 5 Å around the ligand of consOR1 were defined as flexible residues for the flexible receptor preparation. The 20 Å docking grid was aligned based on the centroid of this ligand. The flexible-receptor docking calculation was performed using the AutoDock4 force field. After energy minimization of the complex structure and removal of the ligand, a rigid redocking was performed to redock igands back into the binding sites.

### Molecular Dynamics Simulations

MD simulations of the docked structures of OR1A1 and small molecules were performed using Gromacs. The simulation process comprised the following steps:

System preparation: the structure was prepared using the pdb4amber tool from AmberTools.^39^ Receptor–ligand complexes were placed in a cubic water box with the TIP3P water model, and ions were added to neutralize the system’s charge, maintaining a distance of at least 10 Å from the edge of the box.

Energy minimization: a three-step energy minimization process was conducted to optimize the initial conformation of the system, imposing constraints of 5, 3, and 0 kcal/(mol·Å^2^) on the heavy atoms. All simulations were conducted using the ff14SB force field.^40^

Heating phase: the system was gradually heated from 0 to 300 K over a 1 ns period using a Nosé–Hoover thermostat, while restraints were kept at 3 kcal/(mol·Å^2^), facilitating its approach to equilibrium.

Equilibration: a 10 ns equilibration phase was implemented to ensure stability, with restraints set the same as the heating phase.

Production phase: a 150 ns production MD simulation was conducted in the *NPT* ensemble, with the temperature held at 300 K and the pressure held at 1 atm. The last snapshot of the equilibration phase was used as the initial conformation for three independent production runs, each starting with a unique random seed.

### MD Analysis

RMSD analysis: root mean square deviation (RMSD) values for the heavy atoms of the receptor backbone were calculated using the gmx rms function in Gromacs, providing a comprehensive understanding of the conformational stability of receptor–ligand complexes during simulations.

RMSF analysis: root mean square fluctuation (RMSF) analysis of the C_α_ atoms in the receptor was conducted using the Python package MDAnalysis,^41^ identifying dynamic variations in specific regions such as TM1 (26–29), TM2 (58–79), TM3 (101–120), TM4 (140–158), TM5 (196–218), TM6 (236–258), and TM7 (271–290).

MMGBSA analysis: binding free energy was calculated using molecular mechanics generalized Born surface area (MMGBSA) method.^42^ The final 50 ns snapshots were analyzed to evaluate contributions from individual residues, identifying key residues impacting the energy profile.

Contact frequency and interaction analysis: ligand–receptor interactions were explored using the Python package GetContacts, which measured the frequency of contacts between specific atoms in the complex, revealing crucial interactions that sustained the stability of systems.

## Supplementary Material























## Notes

Supplementary Figures S1–S8, general experimental methods, compound preparation protocols, ^1^H and ^13^C NMR spectra of selected compounds (PDF)
Molecular formula strings and data (CSV)
Homology modeling structure of OR1A1 (PDB)
Docking results of compound **13** with OR1A1 (PDB)
Docking results of compound **14** with OR1A1 (PDB)
Docking results of compound **17** with OR1A1 (PDB)
Docking results of compound **18** with OR1A1 (PDB)
Docking results of compound **20** with OR1A1 (PDB)
Docking results of compound **22** with OR1A1 (PDB)
Docking results of compound (*R*)-**21** with OR1A1 (PDB)
Docking results of compound (*S*)-**21** with OR1A1 (PDB)

## Abbreviations

FL: firefly luciferase
GPCR: G protein-coupled receptor
MD: molecular dynamics
MMGBSA: molecular mechanics generalized Born surface area
OR: olfactory receptor
RL: Renilla luciferase
RMSD: root-mean-square deviation
RMSF: root-mean-square fluctuation
SAR: structure–activity relationship
