# Supplementary material for: Exploring Linear mono-, bis- and tris-Acetylene Containing Agonists of the Human Olfactory Receptor OR1A1
Source: J Med Chem. 2025 Jun 16;68(12):12562–72. doi: 10.1021/acs.jmedchem.5c00282 (PMC12207589; doi:10.1021/acs.jmedchem.5c00282)
Supplement: Supplementary file 1 [file jm5c00282_si_001.pdf]

Supporting Information

Exploring linear *mono*-, *bis*- and *tris*- acetylene containing agonists of the human olfactory receptor OR1A1

Weihong Liu[2‡], Luca S. Dobson[1‡], Chen Zhang[2‡], Jiahui Sun[2], Phillip T Lowe[1], Yingjian Liu[2], Hanyi Zhuang[2*], David O'Hagan[1*]

[1] School of Chemistry, University of St Andrews, North Haught, St Andrews, KY16 9ST, UK.

[2] Intelligent Perception Lab, Hanwang Technology Co., Ltd, Beijing 100193, China.

Corresponding Authors

E.mail do1@st-andrews.ac.uk

E.mail hanyizhuang@hotmail.com

Table of Contents

General experimental methods Pages S3 – S4

Compound preparation protocols Pages S5 – S30

Figure S1. Chiral HPLC (Chiralcel OJ-H) analysis of 1:1 add mixed (*S* + *R*)-**21**. Page S31

Figure S2. Chiral HPLC (Chiralcel OJ-H) analysis of (*R*)-**21**. Page S31

Figure S3. Chiral HPLC (Chiralcel OJ-H) analysis of (S)-**21**. Page S32

NMR of selected compounds Pages S33 – S54

Figure S4. Dose-response curves of the response of OR1A1 to a) the mono-acetylenes **10-12** and b) the *tris*-acetylene **19**. Page S55

Figure S5. The absence of antagonistic activity of Compound **15.** Page S56

Figure S6. Ramachandran plot of OR1A1. Page S57

Figure S7. RMSD profiles from the MD simulations of OR1A1 with compounds **13**, **14**, **17**, **18**, **20**, *(S)*-**21**, and **22.** Page S58

Figure S8. Binding modes of ligands with OR1A1. Page S59

References Pages S60 – S61

General experimental methods

All reactions were carried out under argon or nitrogen atmosphere with standard Schlenk techniques unless otherwise specified. The reaction glassware was flame dried or oven dried overnight and cooled under vacuum. Commercially available chemicals were purchased from Acros, Alfa Aesar, Apollo Scientific, Fisher Scientific, Fluorochem, Manchester Organics, Sigma Aldrich, and TCI (UK) and used as received unless otherwise stated. Anhydrous solvents such as DCM, diethyl ether, THF, toluene and hexane were dried in a solvent purification system (Mbraun MB SPS-200). Anhydrous acetonitrile, DMF, methanol and pentane were purchased as dry solvent and used as received. Room temperature refers to the temperature range 15-27 ^{o}C. The heating temperatures are measured as the temperature of an oil bath or Drysyn heating block. *In vacuo* refers to the use of a rotary evaporator with a membrane pump at 30-50 mbar at 40 ^{o}C unless otherwise stated.

^{1}H, ^{13}C and ^{19}F NMR spectra were recorded on a Bruker AVIII-HD 700 MHz with CryoProbe Prodigy TCI (^{1}H, 700 MHz, ^{13}C, 176 MHz, ^{19}F, 659 MHz), a Bruker AVIII 500 MHz with CyroProbe Prodigy BBO, or a Bruker AVIII-HD 500 MHz with SmartProbe BBFO+ (500 MHz ^{1}H, 126 MHz ^{13}C, and 470 MHz for ^{19}F) or Bruker AV 400 MHz with BBFO probe (400 MHz ^{1}H, 100 MHz ^{13}C, and 376 MHz for ^{19}F). NMR analyses were carried out at room temperature in indicated deuterated solvents unless otherwise noted. Chemical shift data were reported as δ units of ppm corrected by the solvent residue peak and coupling constants, *J*, are reported in Hz. Multiplicities are indicated by: s for singlet, d for doublet, t for triplet, q for quartet and m for multiplet and br. for broad brand. Spectra for novel compounds are shown (see supporting figures), when necessary, resonances were assigned using two-dimensional experiments (COSY, HSQC, HMBC, TOCSY).

Analytical thin layer chromatography was carried out on aluminium backed Merck TLC silica gel 60 F254 plates. These plates were visualised using UV light at 254 nm wavelength, dyed by potassium permanganate or phosphomolybdic acid followed by heating. Flush column chromatography was performed with Sigma-Aldrich silica gel, 60 Å pore size and 230-400 mesh, 40-63 µm particle size under 5 psi compressed air. Melting points were measured on an Electrothermal 9100 melting point apparatus, a Griffin electric thermal melting point

apparatus with thermometer or hot stage microscopy with thermometer reading uncorrected.

Mass spectra measurements were carried at either the University of St Andrews Mass Spectrometer Facility or University of Edinburgh Mass Spectrometer Facility using given methods.

Chiral HPLC analysis was obtained on a Shimadzu HPLC comprising of a DGU-20A5R degassing unit, LC-20AD liquid chromatography pump, SIL-20AHT autosampler, SPD-20A UV/Vis detector and a CTO-20A column oven. Separation was achieved using a Daicel Chiralcel OJ-H column.

Compound preparation protocols

1-(4-(3,3-Dimethylbut-1-yn-1-yl)phenyl)ethan-1-one (10)

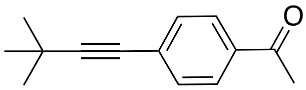

Bis(triphenylphosphine)palladium(II)dichloride (15 mol%, 85.6 mg, 0.122 mmol) was added to a solution of 4'-iodoacetophenone **23** (200 mg, 0.813 mmol), 3,3-dimethyl-1-butyne **25** (0.120 mL, 0.975 mmol), copper (I) iodide (15 mol%, 23.2 mg, 0.122 mmol) in diisopropylamine (7 mL). The reaction mixture was heated to 80 ^{o}C and stirred for 24 h. The reaction was then cooled to room temperature and was passed through a pad of silica gel with an eluant of ethyl acetate. The solvent was removed under reduced pressure to give the crude product, which was purified by column chromatography (0-20% petroleum ether/ethyl acetate) to give the product **10** as a red solid (110.7 mg, 68%). ^{1}H NMR (400 MHz, $CDCl_3$) δ_H 7.84-7.90 (2H, m, Ar**H**), 7.43-7.48 (2H, m, Ar**H**), 2.59 (3H, s, C**H$_3$**), 1.33 (9H, s, (C**H$_3$**)$_3$). Data is in agreement with that reported in the literature.[1]

1-(3-(3,3-Dimethylbut-1-yn-1-yl)phenyl)ethan-1-one (11)

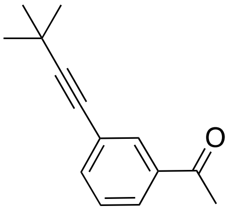

Bis(triphenylphosphine)palladium(II)dichloride (15 mol%, 85.6 mg, 0.122 mmol) was added to a solution of 3'-iodoacetophenone **24** (200 mg, 0.813 mmol), 3,3-dimethyl-1-butyne **25** (0.120 mL, 0.975 mmol), copper (I) iodide (15 mol%, 23.2 mg, 0.122 mmol) in diisopropylamine (7 mL). The reaction mixture was heated to 80 ^{o}C and stirred for 24 h. The reaction was then cooled to room temperature and was passed through a pad of silica gel with an eluant of ethyl acetate. The solvent was removed under reduced pressure to give the crude product, which was purified by column chromatography (0-20% petroleum ether/ethyl acetate) to give the purified product **11** as an orange oil (102.6 mg, 63%). IR ν_{max}/cm^{-1} 2963 (C-H), 2866 (C-H), 1686 (C=O), 1231, 681. ^{1}H NMR (500 MHz, $CDCl_3$) δ_H 7.96 (1H, td, *J* = 1.8, 0.6

Hz, CC**H**C), 7.84 (1H, ddd, *J* = 7.7, 1.8, 1.3 Hz, COCCC**H**CH), 7.56 (1H, dt, *J* = 7.7, 1.3 Hz, CC**H**CH), 7.37 (1H, dt, *J* = 7.7, 0.6 Hz, COCCCHC**H**), 2.60 (3H, s, C**H$_3$**), 1.33 (9H, s, (C**H$_3$**)$_3$). ^{13}C NMR (126 MHz, $CDCl_3$) δ_C 197.8 (C=O), 137.1 (CO**C**), 136.1 (C**C**HCH), 131.7 (C**C**HC), 128.6 (COCCCHC**H**), 127.2 (COCC**C**HCH), 124.8 ((CH_3)$_3$CCC**C**), 99.9 ((CH_3)$_3$C**C**), 78.3 ((CH_3)$_3$CC**C**), 31.1 ((**C**H_3)$_3$), 26.8 (**C**H_3). HRMS (ESI$^+$): Exact mass calculated for $C_{14}H_{17}O^+$ [M+H]$^+$: 201.1279, found: 201.1270.

4-(4-(*Tert*-butyl)phenyl)but-3-yn-2-ol (28)

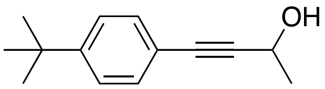

Bis(triphenylphosphine)palladium(II)dichloride (15 mol%, 81.0 mg, 0.115 mmol) was added to a solution of 1-*tert*-butyl-4-iodobenzene **26** (0.136 mL, 0.769 mmol), 3-butyn-2-ol **27** (72.3 μL, 0.923 mmol), copper (I) iodide (15 mol%, 21.9 mg, 0.115 mmol) in diisopropylamine (7 mL). The reaction mixture was heated to 80 ^oC and stirred for 24 h. The reaction was then cooled to room temperature and was passed through a pad of silica gel with an eluant of ethyl acetate. The solvent was removed under reduced pressure to give the crude product. The crude was purified by column chromatography (0-20% petroleum ether/ethyl acetate) to give the purified product **28** as a pale yellow oil (101.1 mg, 65%). ^{1}H NMR (500 MHz, $CDCl_3$) δ_H 7.30-7.39 (4H, m, Ar**H**), 4.76 (1H, q, *J* = 6.6 Hz, C**H**OH), 1.55 (3H, d, *J* = 6.6 Hz, C**H$_3$**), 1.30 (9H, s, (C**H$_3$**)$_3$). Data is in agreement with that reported in the literature.[2]

4-(4-(*Tert*-butyl)phenyl)but-3-yn-2-one (12)

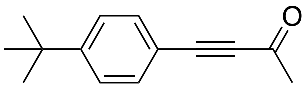

Dess-Martin periodinane (115.3 mg, 0.272 mmol) was added to a solution of 4-(4-(*tert*-butyl)phenyl)but-3-yn-2-ol **28** (50.0 mg, 0.247 mmol) in DCM (1.24 mL) and the reaction was allowed to warm to room temperature and stirred for 3 hours. On completion, the reaction was quenched by addition of 1 M sodium hydroxide solution. The layers were separated and the aqueous layer was extracted three times with DCM. The combined organic layers were

dried over sodium sulfate, filtered and concentrated under reduced pressure to give the crude product, which was purified by column chromatography (20% ethyl acetate/petroleum ether) to give the purified product **12** as a yellow solid (40.3 mg, 81%). ^{1}H NMR (500 MHz, $CDCl_3$) δ_H 7.49-7.54 (2H, m, Ar**H**), 7.37-7.43 (2H, m, Ar**H**), 2.45 (3H, s, C**H₃**), 1.32 (9H, s, (C**H₃**)₃). Data is in agreement with that reported in the literature.[3]

1-(4-((Triisopropylsilyl)ethynyl)phenyl)ethan-1-one (30)

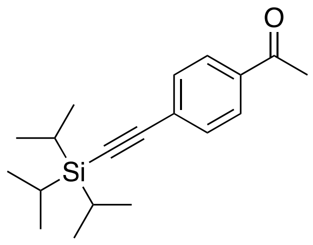

Bis(triphenylphosphine)palladium(II)dichloride (2 mol%, 28.5 mg, 0.0410 mmol) was added to a solution of 4'-iodoacetophenone **23** (500 mg, 2.03 mmol), tri(isopropylsilyl)acetylene **29** (0.729 mL, 3.25 mmol), copper (I) iodide (2 mol%, 28.5 mg, 0.0410 mmol) and triethylamine (5 mL) in THF (5 mL). The reaction mixture was heated to 80 ^{o}C and stirred for 24 h. The reaction was then cooled to room temperature and was passed through a pad of silica gel with an eluant of ethyl acetate. The solvent was removed under reduced pressure to give the crude product, which was purified by column chromatography (0-20% petroleum ether/ethyl acetate) to give the purified product **30** as a colourless oil (500.2 mg, 82%). ^{1}H NMR (400 MHz, $CDCl_3$) δ_H 7.87-7.92 (2H, m, Ar**H**), 7.52-7.57 (2H, m, Ar**H**), 2.60 (3H, s, C**H$_3$**), 1.12-1.15 (21H, m, SiC**H**, SICH(C**H$_3$**)$_2$). Data is in agreement with that reported in the literature.[4]

1-(4-Ethynylphenyl)ethan-1-one (32)

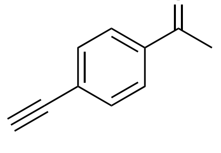

TBAF (1.0 M in THF, 0.429 mL, 0.429 mmol) was added to a solution of 1-(4-((triisopropylsilyl)ethynyl)phenyl)ethan-1-one **30** (100 mg, 0.328 mmol) in THF (0.8 mL) at 0

°C and the reaction was stirred for 3 hours. The reaction was quenched with water and warmed to room temperature. The aqueous layer was extracted three times with DCM. The combined organic layers were dried over sodium sulfate, filtered and concentrated under vacuum to give the crude product, which was was purified by column chromatography (0-20% petroleum ether/ethyl acetate) to give the purified product **32** as a white solid (28.8 mg, 61%). ^{1}H NMR (400 MHz, $CDCl_3$) δ_H 7.90-7.93 (2H, m, Ar**H**), 7.56-7.59 (2H, m, Ar**H**), 3.25 (1H, s, C**H**), 2.61 (3H, s, C**H$_3$**). Data is in agreement with that reported in the literature.[5]

1-(4-(Bromoethynyl)phenyl)ethan-1-one (34)

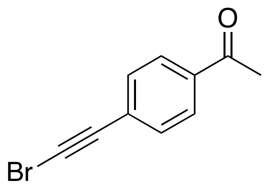

1-(4-Ethynylphenyl)ethan-1-one **32** (93.5 mg, 0.649 mmol) was added to a solution of N-bromosuccinimide (112.2 mg, 0.778 mmol) and silver nitrate (11.0 mg, 10 mol%) in acetone (1 mL) and the solution was stirred at room temperature for 3 hours. On completion, the solvent was removed under reduced pressure. The resulting residue was dissolved in water (3 mL) and the aqueous layer was extracted with diethyl ether (3 x 3 mL). The combined organic layers were washed with brine (5 mL), dried over magnesium sulfate, filtered and concentrated under reduced pressure. The crude product was purified by column chromatography (20% ethyl acetate in petroleum ether) to give the purified product **34** as a white solid (44.4 mg, 31%). ^{1}H NMR (400 MHz, $CDCl_3$) δ_H 7.88-7.92 (2H, m, Ar**H**), 7.51-7.56 (2H, m, Ar**H**), 2.60 (3H, s, C**H$_3$**). Data is in agreement with that reported in the literature.[6]

1-(4-(5,5-Dimethylhexa-1,3-diyn-1-yl)phenyl)ethan-1-one (13)

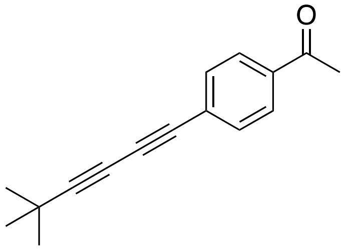

A 30% butylamine/water solution (0.5 mL) was added to a RBF containing copper(I) chloride (4.93 mg, 0.0498 mmol) at 0 °C. A hydroxylamine hydrochloride was then added slowly until the blue colour was no longer visible. A solution of 3,3-dimethyl-1-butyne **25** (20.4 μL, 0.166 mmol) in DCM (0.1 mL) was added, quickly followed by a solution of 1-(4-(bromoethynyl)phenyl)ethan-1-one **34** (44.4 mg, 0.199 mmol) in DCM (0.1 mL). The reaction was warmed to room temperature and stirred for 3 hours. On completion, the aqueous layer was extracted with DCM, and the organic layer was dried over sodium sulfate, filtered and concentrated under vacuum. The crude product was purified by column chromatography (0 to 20% petroleum ether/ethyl acetate) to give pure **13** as a yellow solid (25.3 mg, 53%). m.p. 173-175 °C. IR ν_{max}/cm^{-1} 2970 (C-H), 1670 (C=O), 1358, 1260, 1179, 833. ^{1}H NMR (500 MHz, $CDCl_3$) δ_H 7.87-7.90 (2H, m, C**H**CCO), 7.52-7.55 (2H, m, C**H**CHCCO), 2.59 (3H, s, C**H$_3$**), 1.29 (9H, s, (C**H$_3$**)$_3$). ^{13}C NMR (126 MHz, $CDCl_3$) δ_C 197.3 (**C**=O), 136.7 (**C**CO), 132.7, 128.3, 127.2 (CH**C**CCCC), 94.3 (**C**C(CH_3)$_3$), 77.5 (alkyne C), 75.1 (CHC**C**CC), 63.7 (alkyne C), 30.6 ((**C**H$_3$)$_3$), 26.8 (**C**H$_3$). HRMS (ESI$^+$): Exact mass calculated for $C_{16}H_{17}O^+$ [M+H]$^+$: 225.1274, found: 225.1272.

1-(3-((Triisopropylsilyl)ethynyl)phenyl)ethan-1-one (31)

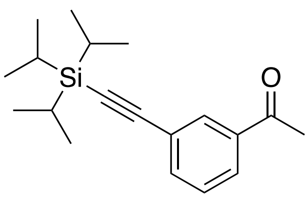

Bis(triphenylphosphine)palladium(II)dichloride (2 mol%, 28.5 mg, 0.0410 mmol) was added to a solution of 3’-iodoacetophenone **24** (500 mg, 2.03 mmol), tri(isopropylsilyl)acetylene **29** (0.729 mL, 3.25 mmol), copper (I) iodide (2 mol%, 28.5 mg, 0.0410 mmol) and triethylamine (5 mL) in THF (5 mL). The reaction mixture was heated to 80 °C and stirred for 24 h. The

reaction was then cooled to room temperature and was passed through a pad of silica gel with an eluant of ethyl acetate. The solvent was removed under reduced pressure to give the crude product, which was purified by column chromatography (0-25% petroleum ether/ethyl acetate) to give the purified product **31** as a colourless oil (274.6 mg, 42%). ^{1}H NMR (400 MHz, $CDCl_3$) δ_H 8.03 (1H, td, *J* = 1.8, 0.5 Hz, Ar**H**), 7.89 (1H, ddd, *J* = 7.8, 1.8, 1.3 Hz, Ar**H**), 7.66 (1H, dt, *J* = 7.8, 1.3 Hz, ArH), 7.41 (1H, td, *J* = 7.8, 0.5 Hz, ArH), 2.61 (3H, s, C**H₃**), 1.14 (21H, m, SiC**H**, SICH(C**H₃**)₂). Data is in agreement with that reported in the literature.[7]

1-(3-Ethynylphenyl)ethan-1-one (33)

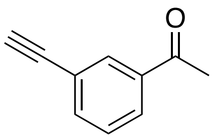

TBAF (1.0 M in THF, 0.844 mL, 0.844 mmol) was added to a solution of 1-(3-((triisopropylsilyl)ethynyl)phenyl)ethan-1-one **31** (195.0 mg, 0.914 mmol) in THF (1.5 mL) at 0 °C and the reaction was stirred for 3 hours. The reaction was quenched with water and warmed to room temperature. The aqueous layer was extracted with DCM (3x). The combined organic layers were dried over sodium sulfate, filtered and concentrated under vacuum to give the crude product, which was purified by column chromatography (0-20% petroleum ether/ethyl acetate) to give the purified product **33** as a white solid (64.8 mg, 69%). ^{1}H NMR (400 MHz, $CDCl_3$) δ_H 8.07 (1H, t, *J* = 1.7 Hz, Ar**H**), 7.94 (1H, dt, *J* = 7.8, 1.4 Hz, Ar**H**), 7.68 (1H, dt, *J* = 7.8, 1.4 Hz, Ar**H**), 7.44 (1H, td, *J* = 7.8, 0.6 Hz, Ar**H**), 3.14 (1H, s, C**H**), 2.61 (3H, s, C**H₃**). Data is in agreement with that reported in the literature.[8]

1-(3-(Bromoethynyl)phenyl)ethan-1-one (35)

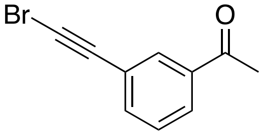

1-(3-Ethynylphenyl)ethan-1-one **33** (64.8 mg, 0.449 mmol) was added to a solution of N-bromosuccinimide (96.0 mg, 0.539 mmol) and silver nitrate (7.63 mg, 0.0449 mmol) in acetone (1 mL) and the solution was stirred at room temperature for 3 hours. On completion

the solvent was removed under reduced pressure, the resulting residue was dissolved in water (3 mL) and the aqueous layer was extracted with diethyl ether (3 x 3 mL). The combined organic layers were washed with brine (5 mL), dried over magnesium sulfate, filtered and concentrated under reduced pressure. The crude product was purified by column chromatography (20% ethyl acetate in petroleum ether) to give the pure **35** as a white solid (66.6 mg, 66%). m.p. 61-63 °C. IR ν_{max}/cm^{-1} 2913 (C-H), 1660 (C=O), 1445, 1380, 1302, 805. ^{1}H NMR (500 MHz, $CDCl_3$) δ_H 8.03 (1H, t, *J* = 1.8 Hz, BrCCCC**H**CO), 7.93 (1H, dt, *J* = 7.9, 1.5 Hz, CHC**H**CCO), 7.63 (1H, dt, *J* = 7.7, 1.5 Hz, BrCCCC**H**CH), 7.43 (1H, t, *J* = 7.9 Hz, BrCCCCHC**H**), 2.60 (3H, s, C**H₃**). ^{13}C NMR (126 MHz, $CDCl_3$) δ_C 197.2 (**C**=O), 137.4 (**C**CO), 136.3 (BrCCC**C**HCH), 132.2 (BrCCC**C**HCO), 128.9 (COCCHC**H**), 128.4 (COC**C**HCH), 123.5 (BrCC**C**), 79.2 (alkyne **C**), 51.5 (alkyne **C**), 26.8 (**C**H_3). HRMS (ESI$^+$): Exact mass calculated for $C_{10}H_8{}^{79}BrO^+$ $[M+H]^+$: 222.9753, found: 222.9749.

1-(3-(5,5-Dimethylhexa-1,3-diyn-1-yl)phenyl)ethan-1-one (14)

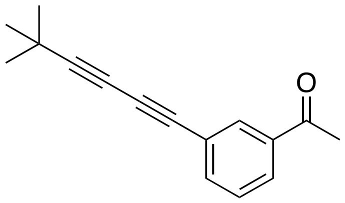

A 30% butylamine/water solution (0.844 mL) was added to an RBF containing copper(I) chloride (7.40 mg, 0.0747 mmol) at 0 °C, hydroxylamine hydrochloride was then added until the blue colour disappeared. A solution of 3,3-dimethyl-1-butyne **25** (30.6 μL, 0.166 mmol) in DCM (0.15 mL) was added, quickly followed by a solution of 1-(3-(bromoethynyl)phenyl)ethan-1-one **35** (66.6 mg, 0.299 mmol) in DCM (0.15 mL). The reaction was warmed to room temperature and stirred for 3 hours. On completion, the aqueous layer was extracted with DCM. The organic layer was dried over sodium sulfate, filtered and concentrated under vacuum to give the crude product, which was purified by column chromatography (0 to 20% petroleum ether/ethyl acetate) to give the purified product **14** as a yellow solid (41.1 mg, 61%). m.p. 121-123 °C. ^{1}H NMR (500 MHz, $CDCl_3$) δ_H 8.03 (1H, t, *J* = 1.7 Hz, CC**H**CCO), 7.91 (1H, dd, *J* = 7.9, 1.7, 1.3 Hz, CHC**H**CCO), 7.63 (1H, dt, *J* = 7.7, 1.3 Hz, CCC**H**CH), 7.41 (1H, t, *J* = 7.9 Hz, COCCHC**H**CH), 2.58 (3H, s, C**H₃**), 1.29 (9H, s, (C**H₃**)₃). ^{13}C NMR

(126 MHz, $CDCl_3$) δ_C 197.3 (**C**=O), 137.3 (**C**CO), 136.6 (CC**C**HCH), 132.6 (C**C**HCCO), 128.9 (COCCHC**H**), 128.4 (CH**C**HCCO), 123.0 (BrCC**C**), 93.2, 75.3 (alkyne C), 74.9, 63.6 (alkyne C), 30.6 ((**C**H_3)$_3$), 26.8 (**C**H_3). HRMS (ESI$^+$): Exact mass calculated for $C_{16}H_{17}O^+$ [M+H]$^+$: 225.1279, found: 225.1271.

4-Bromobut-3-yn-2-ol (36)

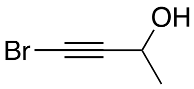

3-Butyn-2-ol **27** (0.559 mL, 7.13 mmol) was added to a solution of N-bromosuccinimide (1.40 g, 7.85 mmol) and silver nitrate (121.2 mg, 0.713 mmol) in acetone (12 mL) and the solution was stirred at room temperature for 3 hours. On completion, the solvent was removed under reduced pressure. The resulting residue was dissolved in water (3 mL) and the aqueous layer was extracted with diethyl ether (3 x 3 mL). The combined organic layers were washed with brine (5 mL), dried over magnesium sulfate, filtered and concentrated under reduced pressure. The crude was purified by column chromatography (20% ethyl acetate in petroleum ether) to give the purified product **36** as a white solid (445.1 mg, 42%). ^{1}H NMR (400 MHz, $CDCl_3$) δ_H 4.55 (1H, q, *J* = 6.6 Hz, C**H**OH), 1.46 (3H, d, *J* = 6.6 Hz, C**H$_3$**). Data is in agreement with that reported in the literature.[9]

6-(4-(*tert*-Butyl)phenyl)hexa-3,5-diyn-2-ol (21)

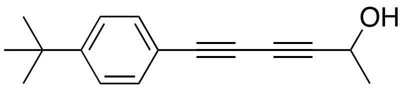

A 30% butylamine/water solution (2.16 mL) was added to an RBF containing copper(I) chloride (18.8 mg, 0.190 mmol) at 0 ^{o}C, hydroxylamine hydrochloride was then added until the blue colour disappeared. A solution of 4-(*tert*-butyl)phenylacetylene **37** (0.112 mL, 0.632 mmol) in DCM (0.82 mL) was added, quickly followed by a solution of 4-bromobut-3-yn-2-ol **36** (113.0 mg, 0.758 mmol) in DCM (0.82 mL). The reaction was warmed to room temperature and stirred for 3 hours. On completion, the aqueous layer was extracted with DCM and the organic layer was dried over sodium sulfate, filtered and concentrated under vacuum to give the

crude product. The crude product was purified by column chromatography (0 to 20% petroleum ether/ethyl acetate) to give the purified product **21** as a white solid (196.0 mg, 73%). m.p. 77-79 °C. IR ν_{max}/cm^{-1} 3302 (O-H), 2957 (C-H), 1367 (O-H), 1074 (O-H), 1067 (O-H), 836. ^{1}H NMR (500 MHz, $CDCl_3$) δ_H 7.41-7.44 (2H, m, $(CH_3)_3$CCC**H**CH), 7.32-7.36 (2H, m, $(CH_3)_3$CCCHC**H**), 4.66 (1H, q, *J* = 6.6 Hz, C**H**OH), 1.52 (3H, d, *J* = 6.6 Hz, C**H₃**), 1.30 (9H, s, (C**H₃**)₃). HRMS (ESI$^+$): Exact mass calculated for $C_{16}H_{18}ONa^+$ [M+Na]$^+$: 249.1250, found: 249.1250.

6-(4-(*tert*-Butyl)phenyl)hexa-3,5-diyn-2-one (15)

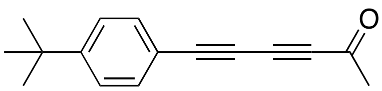

Dess-Martin periodinane (206.1 mg, 0.486 mmol) was added to a solution of 6-(4-(*tert*-butyl)phenyl)hexa-3,5-diyn-2-ol **21** (100.0 mg, 0.442 mmol) in DCM (2.22 mL) and the reaction was allowed to warm to room temperature and stirred for 3 hours. On completion, the reaction was quenched by addition of 1 M sodium hydroxide solution, the layers were separated and the aqueous layer was extracted three times with DCM. The combined organic layers were dried over sodium sulfate, filtered and concentrated under reduced pressure to give the crude product, which was purified by column chromatography (20% ethyl acetate/petroleum ether) to give the purified product **15** as an orange solid (64.4 mg, 65%). m.p. 141-143 °C. IR ν_{max}/cm^{-1} 2961 (C-H), 2203 (C-C alkyne), 1672 (C=O), 1603 (C=O), 1362, 1267, 1107, 908, 835. ^{1}H NMR (500 MHz, $CDCl_3$) δ_H 7.47-7.50 (2H, m, $(CH_3)_3$CCC**H**), 7.37-7.40 (2H, m, $(CH_3)_3$CCCHC**H**), 2.41 (3H, s, C**H₃**), 1.32 (9H, s, (C**H₃**)₃). ^{13}C NMR (126 MHz, $CDCl_3$) δ_C 183.6 (C=O), 154.4 (($CH_3)_3$C**C**), 133.0 (2C, $(CH_3)_3$CC**C**H), 125.9 (2C, $(CH_3)_3$CCCH**C**H), 117.2 (CH**C**CCCCCO), 87.2 (CHC**C**), 78.8 (**C**CO), 75.7 (alkyne C), 71.8 (alkyne C), 35.2 (**C**$(CH_3)_3$), 32.8 (**C**H_3), 31.2 (3C, (**C**$H_3)_3$). HRMS (ESI$^+$): Exact mass calculated for $C_{16}H_{16}ONa^+$ [M+Na]$^+$: 247.1093, found: 247.1087.

1-(3-((Triisopropylsilyl)buta-1,3-diyn-1-yl)phenyl)ethan-1-one (38)

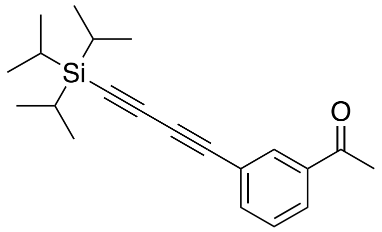

A 30% butylamine/water solution (2.52 mL) was added to an RBF containing copper(I) chloride (22.2 mg, 0.224 mmol) at 0 °C, hydroxylamine hydrochloride was added until the blue colour disappeared. A solution of tri(isopropylsilyl)acetylene **29** (0.167 mL, 0.747 mmol) in DCM (2 mL) was added, quickly followed by a solution of 1-(3-(bromoethynyl)phenyl)ethan-1-one **34** (200 mg, 0.897 mmol) in DCM (2 mL). The reaction was warmed to room temperature and stirred for 3 hours. On completion, the aqueous layer was extracted with DCM. The organic layer was dried over sodium sulfate, filtered and concentrated under vacuum to give the crude product, which was purified by column chromatography (0 to 20% petroleum ether/ethyl acetate) to give the purified product as a yellow solid **38** (203.7 mg, 84%). m.p. 82-84 °C. IR ν_{max}/cm^{-1} 2943 (C-H), 2864 (C-H), 1686 (C=O), 1360, 1231, 883, 804, 679. ^{1}H NMR (500 MHz, $CDCl_3$) δ_H 8.08 (1H, t, *J* = 1.7 Hz, CC**H**CCO), 7.94 (1H, dt, *J* = 7.8, 1.4 Hz, CHC**H**CCO), 7.68 (1H, dt, *J* = 7.8, 1.4 Hz, C**H**CHCHCCO), 7.43 (1H, t, *J* = 7.8 Hz, C**H**CHCCO), 2.59 (3H, s, C**H₃**), 1.10-1.14 (21H, m, SiC**H**, SICH(C**H₃**)₂). ^{13}C NMR (126 MHz, $CDCl_3$) δ_C 197.1 (C=O), 137.4 (**C**CO), 136.9 (**C**HCHCHCCO), 132.9 (C**C**HCCO), 129.0 (**C**HCHCCO), 128.8 (CH**C**HCCO), 122.4 (SiCCCC**C**), 89.2 (alkyne **C**), 89.1 (alkyne **C**), 75.8 (alkyne **C**), 74.5 (SiCCC**C**), 26.8 (**C**H₃), 18.7 (6C, (**C**H₃)₂), 11.4 (3C, **C**H(CH₃)₂). HRMS (ESI$^+$): Exact mass calculated for $C_{21}H_{29}OSi^+$ [M+H]$^+$: 325.1988, found: 325.1973.

1-(3-(Buta-1,3-diyn-1-yl)phenyl)ethan-1-one (39)

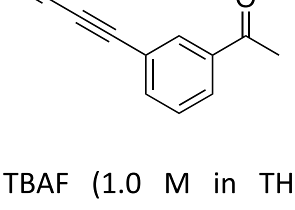

TBAF (1.0 M in THF, 0.329 mL, 0.329 mmol) was added to a solution of 1-(3-((triisopropylsilyl)buta-1,3-diyn-1-yl)phenyl)ethan-1-one **38** (82.0 mg, 0.253 mmol) in THF

(0.62 mL) at 0 °C and the reaction was stirred for 3 hours. The reaction was quenched with water, warmed to room temperature and the aqueous layer was extracted three times with DCM. The combined organic layers were dried over sodium sulfate, filtered and concentrated under vacuum to give the crude product. The crude product was purified by column chromatography (0-20% petroleum ether/ethyl acetate) to give the purified product as a yellow solid **39** (10.0 mg, 23%). m.p. 141-143 °C. IR ν_{max}/cm^{-1} 2963 (C-H), 2916 (C-H), 1684 (C=O), 1260, 1016, 795. ^{1}H NMR (500 MHz, $CDCl_3$) δ_H 8.09 (1H, t, *J* = 1.6 Hz, CC**H**CCO), 7.96 (1H, dt, *J* = 7.8, 1.6 Hz, CHC**H**CCO), 7.69 (1H, dt, *J* = 7.8, 1.6 Hz, C**H**CHCHCCO), 7.45 (1H, t, *J* = 7.8 Hz, C**H**CHCCO), 2.60 (3H, s, C**H₃**), 2.51 (1H, s, alkyne C**H**). HRMS (ESI$^+$): Exact mass calculated for $C_{12}H_9O^+$ $[M+H]^+$: 169.0648, found: 169.0644.

1-Bromo-3,3-dimethylbut-1-yne (40)

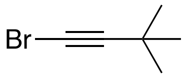

3,3-Dimethylbut-1-yne **25** (1.5 mL, 12.2 mmol) was added to a solution of N-bromosuccinimide (2.38 g, 13.4 mmol) and silver nitrate (207.2 mg, 1.22 mmol) in acetone (60 mL) and the solution was stirred at room temperature for 3 hours. On completion, the solvent was removed under reduced pressure. The resulting residue was dissolved in water (35 mL) and the aqueous layer was extracted with diethyl ether (3 x 35 mL). The combined organic layers were washed with brine (90 mL), dried over magnesium sulfate, filtered and concentrated under reduced pressure. The crude was purified by column chromatography (20% ethyl acetate in petroleum ether) to give the purified product **40** as a colourless liquid (216.4 mg, 11%). ^{1}H NMR (500 MHz, $CDCl_3$) δ_H 1.23 (9H, s, (C**H₃**)₃). ^{13}C NMR (126 MHz, $CDCl_3$) δ_C 88.4 (alkyne **C**), 37.2 (alkyne **C**), 30.8 ((**C**H₃)₃), 27.8 ((CH₃)₃**C**). Data is in agreement with that reported in the literature.[10]

1-(3-(7,7-Dimethylocta-1,3,5-triyn-1-yl)phenyl)ethan-1-one (19)

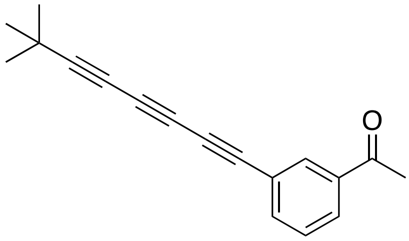

A 30% butylamine/water solution (0.2 mL) was added to an RBF containing copper(I) chloride (1.76 mg, 0.0178 mmol) at 0 ^{o}C, hydroxylamine hydrochloride was added until the blue colour disappeared. A solution of 1-(3-(buta-1,3-diyn-1-yl)phenyl)ethan-1-one **39** (10.0 mg, 0.0592 mmol) in DCM (0.1 mL) was then added, quickly followed by a solution of 1-bromo-3,3-dimethylbut-1-yne **40** (11.4 mg, 0.0710 mmol) in DCM (0.1 mL). The reaction was warmed to room temperature and stirred for 3 hours. On completion, the aqueous layer was extracted with DCM, and the organic layer dried over sodium sulfate, filtered and concentrated under vacuum to give the crude product. The crude product was purified by column chromatography (0 to 20% petroleum ether/ethyl acetate) to give the purified product **19** as a yellow solid (4.0 mg, 27%). m.p. 102-104 ^{o}C. ^{1}H NMR (500 MHz, $CDCl_3$) δ_H 8.07 (1H, td, *J* = 1.8, 0.5 Hz, CC**H**CCO), 7.95 (1H, dt, *J* = 7.8, 1.8, 1.3 Hz, CHC**H**CCO), 7.68 (1H, dt, *J* = 7.8, 1.3 Hz, C**H**CHCHCCO), 7.43 (1H, td, *J* = 7.8, 0.5 Hz, C**H**CHCCO), 2.60 (3H, s, C**H$_3$**), 1.12 (9H, s, (C**H$_3$**)$_3$). HRMS (ESI$^+$): Exact mass calculated for $C_{18}H_{17}O^+$ [M+H]$^+$: 249.1279, found: 249.1267.

6-((Triisopropylsilyl)ethynyl)-2,3-dihydro-1*H*-inden-1-one (43)

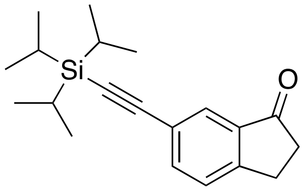

Bis(triphenylphosphine)palladium(II)dichloride (2 mol%, 33.3 mg, 0.0474 mmol) was added to a solution of 6-bromo-1-indanone **41** (500 mg, 2.37 mmol), tri(isopropylsilyl)acetylene **29** (0.850 mL, 3.79 mmol), copper (I) iodide (2 mol%, 9.03 mg, 0.0474 mmol) and triethylamine (5.84 mL) in THF (5.84 mL), the reaction mixture was then heated to 80 ^{o}C and stirred for 24 h. The reaction was then cooled to room temperature and was passed through a pad of silica

gel with an eluant of ethyl acetate. The solvent was removed under reduced pressure to give the crude product, which was purified by column chromatography (0-25% petroleum ether/ethyl acetate) to give the purified product **43** as a colourless oil (585.1 mg, 79%). m.p. 84-86 °C. IR ν_{max}/cm^{-1} 2940 (C-H), 2862 (C-H), 2156 (C-C alkyne), 1711 (C=O), 1287, 881, 839, 662. ^1H NMR (400 MHz, $CDCl_3$) δ_H 7.86 (1H, dd, *J* = 1.6, 0.9 Hz, C**H**CCO), 7.67 (1H, dd, *J* = 7.9, 1.6 Hz, C**H**CCHCCO), 7.42 (1H, dd, *J* = 7.9, 0.9 Hz, C**H**CCH_2CH_2), 3.13-3.16 (2H, m, C$\mathbf{H_2}$$CH_2$CO), 2.70-2.73 (2H, m, C$\mathbf{H_2}$O), 1.11-1.13 (21H, s, SiC**H**, SICH(C$\mathbf{H_3}$)$_2$). HRMS (ESI^+): Exact mass calculated for $C_{20}H_{28}OSiNa^+$ $[M+Na]^+$: 335.1087, found: 335.1802.

6-Ethynyl-2,3-dihydro-1*H*-inden-1-one (45)

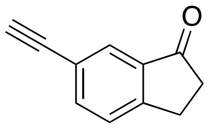

TBAF (1.0 M in THF, 0.876 mL, 0.876 mmol) was added to a solution of 6-((triisopropylsilyl)ethynyl)-2,3-dihydro-1*H*-inden-1-one **43** (247.3 mg, 0.791 mmol) in THF (1.92 mL) at 0 °C and the reaction was stirred for 3 hours, after which the reaction was quenched with water and warmed to room temperature. The aqueous layer was extracted three times with DCM. The combined organic layers were dried over sodium sulfate, filtered and concentrated under vacuum to give the crude product, which was purified by column chromatography (0-20% petroleum ether/ethyl acetate) to give the purified product **45** as a yellow oil (66.3 mg, 54%). IR ν_{max}/cm^{-1} 2361, 2342, 1559 (C=O), 1541 (C=O). ^1H NMR (400 MHz, $CDCl_3$) δ_H 7.87 (1H, s, ArH), 7.68 (1H, dd, *J* = 7.9, 1.6 Hz, C**H**CCHCCO), 7.44 (1H, dp, *J* = 7.9, 0.9 Hz, C**H**CCH_2CH_2), 3.14-3.17 (2H, m, C$\mathbf{H_2}$$CH_2$CO), 3.10 (1H, s, alkyne-**H**), 2.70-2.73 (2H, m, C$\mathbf{H_2}$O). HRMS (EI): Exact mass calculated for $C_{11}H_8O$: 156.0570, found: 155.0566.

6-(Bromoethynyl)-2,3-dihydro-1*H*-inden-1-one (47)

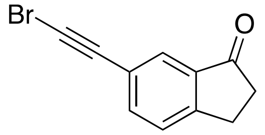

6-Ethynyl-2,3-dihydro-1*H*-inden-1-one **45** (66.3 mg, 0.425 mmol) was added to a solution of N-bromosuccinimide (90.7 mg, 0.509 mmol) and silver nitrate (7.22 mg, 0.0425 mmol) in acetone (1 mL) and the solution was stirred at room temperature for 3 hours. On completion, the solvent was removed under reduced pressure, the resulting residue was dissolved in water (1 mL) and the aqueous layer was extracted with diethyl ether (3 x 1 mL). The combined organic layers were washed with brine (5 mL), dried over magnesium sulfate, filtered and concentrated under reduced pressure, and the crude product purified by column chromatography (20% ethyl acetate in petroleum ether) to give the purified product **47** as a colourless oil (41.6 mg, 42%). IR ν_{max}/cm^{-1} 2922 (C-H), 2864 (C-H), 2361, 1686 (C=O), 1231, 679 (C-Br). ^1H NMR (400 MHz, $CDCl_3$) δ_H 7.83 (1H, s, Ar**H**), 7.64 (1H, dd, *J* = 7.9, 1.6 Hz, C**H**CCHCCO), 7.43 (1H, d, *J* = 7.9 Hz, C**H**CCH$_2$CH$_2$), 3.13-3.16 (2H, m, C**H$_2$**CH$_2$CO), 2.70-2.73 (2H, m, C**H$_2$**O). HRMS (EI): Exact mass calculated for $C_{11}H_7O^{79}Br$: 233.9675, found: 233.9677.

6-(5,5-Dimethylhexa-1,3-diyn-1-yl)-2,3-dihydro-1*H*-inden-1-one (16)

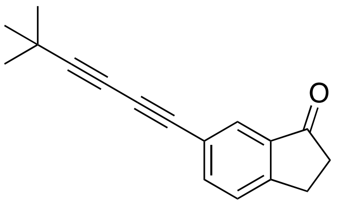

A 30% butylamine/water solution (0.5 mL) was added to an RBF containing copper(I) chloride (4.37 mg, 0.177 mmol) at 0 °C, hydroxylamine hydrochloride was then added until the blue colour disappeared. A solution of 3,3-dimethylbut-1-yne **25** (18.2 µL, 0.147 mmol) in DCM (0.2 mL) was added, quickly followed by a solution of 6-(bromoethynyl)-2,3-dihydro-1*H*-inden-1-one **47** (41.6 mg, 0.177 mmol) in DCM (0.2 mL). The reaction was warmed to room temperature and stirred for 3 hours. On completion, the aqueous layer was extracted with DCM and the organic layer was dried over sodium sulfate, filtered and concentrated under vacuum to give the crude product. The crude product was purified by column

chromatography (0 to 20% petroleum ether/ethyl acetate) to give the purified product as a yellow solid **16** (18.8 mg, 45%). m.p. 135-137 °C. IR v_{max}/cm^{-1} 2922 (C-H), 2361 (alkyne C-C), 1653 (C=O), 1558, 1506, 1456, 1057. ^{1}H NMR (400 MHz, $CDCl_3$) δ_H 7.83 (1H, dd, *J* = 1.6, 0.8 Hz, CC**H**CCO), 7.65 (1H, dd, *J* = 7.9, 1.6 Hz, C**H**CCHCCO), 7.42 (1H, dq, *J* = 7.9, 0.8 Hz, C**H**CCH_2CH_2), 3.12-3.15 (2H, m, C$\mathbf{H_2}$CH_2CO), 2.69-2.72 (2H, m, C$\mathbf{H_2}$CO), 1.29 (9H, s, (C$\mathbf{H_3}$)$_3$). ^{13}C NMR (126 MHz, $CDCl_3$) δ_C 206.0 (**C**=O), 155.5 (**C**CH_2CH_2), 138.3 (**C**HCCHCCO), 137.4 (**C**CO), 127.8 (C**C**HCCO), 127.0 (**C**HCCH_2CH_2), 121.8 (CCCC**C**CH), 93.0 ((CH_3)$_3$C**C**), 75.0 (alkyne C), 74.9 (alkyne C), 63.7 ((CH_3)$_3$CC**C**), 36.5 (**C**H_2CO), 30.6 (3C, (**C**H_3)$_3$), 29.9 (**C**(CH_3)$_3$), 26.1 (**C**H_2CH_2CO). HRMS (ESI$^+$): Exact mass calculated for $C_{17}H_{17}O$ [M+H]$^+$: 237.1279, found: 237.1267.

4-((Triisopropylsilyl)ethynyl)-2,3-dihydro-1*H*-inden-1-one (44)

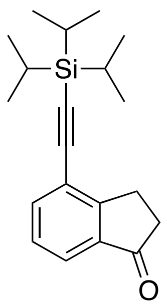

Bis(triphenylphosphine)palladium(II)dichloride (2 mol%, 33.3 mg, 0.0474 mmol) was added to a solution of 4-bromo-1-indanone **42** (500 mg, 2.37 mmol), tri(isopropylsilyl)acetylene **29** (0.850 mL, 3.79 mmol), copper (I) iodide (2 mol%, 9.03 mg, 0.0474 mmol) and triethylamine (5.84 mL) in THF (5.84 mL). The reaction mixture was heated to 80 °C and stirred for 24 h. The reaction was then cooled to room temperature and was passed through a pad of silica gel with an eluant of ethyl acetate. The solvent was removed under reduced pressure to give the crude product, which was purified by column chromatography (0-25% petroleum ether/ethyl acetate) to give the purified product **44** as a colourless oil (228.0 mg, 31%). ^{1}H NMR (400 MHz, $CDCl_3$) δ_H 7.67-7.73 (2H, m, Ar**H**), 7.31-7.37 (1H, m, Ar**H**), 3.17-3.23 (2H, m, C$\mathbf{H_2}$CH_2CO), 2.70-2.75 (2H, m, C$\mathbf{H_2}$CO), 1.13-1.19 (21H, m, SiC**H**, SICH(C$\mathbf{H_3}$)$_2$). Data is in agreement with that reported in the literature.[11]

4-Ethynyl-2,3-dihydro-1*H*-inden-1-one (46)

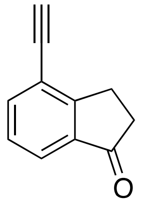

TBAF (1.0 M in THF, 0.948 mL, 0.948 mmol) was added to a solution of 4-((triisopropylsilyl)ethynyl)-2,3-dihydro-1*H*-inden-1-one **44** (228.0 mg, 0.730 mmol) in THF (1.78 mL) at 0 °C and the reaction was stirred for 3 hours. The reaction was quenched with water and warmed to room temperature. The aqueous layer was extracted with DCM (3x). The combined organic layers were dried over sodium sulfate, filtered and concentrated under vacuum to give the crude product. The crude product was purified by column chromatography (0-20% petroleum ether/ethyl acetate) to give the purified product **46** as a yellow solid (56.1 mg, 49%). m.p. 150-152 °C. IR ν_{max}/cm^{-1} 2972 (C-H), 2922 (C-H), 2361 (alkyne C-C), 1786 (C=O), 1697 (C=O), 1327, 1200, 1065, 702. ^{1}H NMR (400 MHz, $CDCl_3$) δ_H 7.74 (1H, dd, *J* = 7.5, 1.1 Hz, Ar**H**), 7.70 (1H, dd, *J* = 7.5, 1.1 Hz, Ar**H**), 7.36 (1H, tt, *J* = 7.5, 1.1 Hz, Ar**H**), 3.36 (1H, s, alkyne H), 3.18-3.21 (2H, m, C$\mathbf{H_2}$$CH_2$CO), 2.71-2.74 (2H, m, CH_2C$\mathbf{H_2}$CO). HRMS (ESI$^+$): Exact mass calculated for $C_{11}H_8ONa^+$ [M+Na]$^+$: 179.0473, found: 179.0467.

4-(Bromoethynyl)-2,3-dihydro-1*H*-inden-1-one (48)

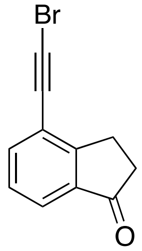

4-Ethynyl-2,3-dihydro-1*H*-inden-1-one **46** (56.1 mg, 0.359 mmol) was added to a solution of N-bromosuccinimide (76.7 mg, 0.431 mmol) and silver nitrate (6.10 mg, 0.0359 mmol) in acetone (1 mL) and the solution was stirred at room temperature for 3 hours. On completion, the solvent was removed under reduced pressure, the resulting residue was dissolved in water (1 mL) and the aqueous layer was extracted with diethyl ether (3 x 1 mL). The combined organic layers were washed with brine (5 mL), dried over magnesium sulfate, filtered and

concentrated under reduced pressure. The crude product was purified by column chromatography (20% ethyl acetate in petroleum ether) to give the purified product as a yellow solid **48** (50.3 mg, 60%). m.p. 168-170 °C. IR ν_{max}/cm^{-1} 2361 (alkyne C-C), 2342 (alkyne C-C), 1653 (C=O), 1558 (C=O), 1506. ^{1}H NMR (400 MHz, $CDCl_3$) δ_H 7.73 (1H, dd, *J* = 7.6, 1.0 Hz, Ar**H**), 7.66 (1H, dd, *J* = 7.6, 1.0 Hz, Ar**H**), 7.35 (1H, tt, *J* = 7.6, 1.0 Hz, Ar**H**), 3.17-3.20 (2H, m, C**H₂**CH₂CO), 2.70-2.73 (2H, m, CH₂C**H₂**CO). HRMS (ESI$^+$): Exact mass calculated for $C_{11}H_7{}^{79}BrONa^+$ [M+Na]$^+$: 256.9578, found: 256.9573.

4-(5,5-Dimethylhexa-1,3-diyn-1-yl)-2,3-dihydro-1*H*-inden-1-one (17)

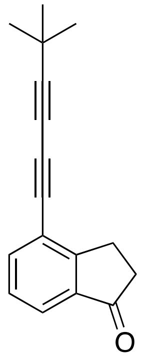

A 30% butylamine/water solution (0.6 mL) was added to an RBF containing copper(I) chloride (2.11 mg, 0.0214 mmol) at 0 °C, hydroxylamine hydrochloride was added until the blue colour disappeared. A solution of 3,3-dimethylbut-1-yne **25** (22.0 μL, 0.178 mmol) in DCM (0.2 mL) was added, quickly followed by a solution of 4-(bromoethynyl)-2,3-dihydro-1*H*-inden-1-one **48** (50.3 mg, 0.214 mmol) in DCM (0.2 mL). The reaction was warmed to room temperature and stirred for 3 hours. On completion, the aqueous layer was extracted with DCM, the organic layer was dried over sodium sulfate, filtered and concentrated under vacuum to give the crude product. The crude product was purified by column chromatography (0 to 20% petroleum ether/ethyl acetate) to give the purified product as a yellow solid **17** (18.9 mg, 59%). m.p. 78-80 °C. IR ν_{max}/cm^{-1} 2965 (C-H), 2924 (C-H), 2231 (alkyne C-C), 1708 (C=O), 1558 (C=O), 1325, 1260, 1032, 781. ^{1}H NMR (500 MHz, $CDCl_3$) δ_H 7.72 (1H, dd, *J* = 7.6, 1.1 Hz, C**H**CCO), 7.68 (1H, dd, *J* = 7.6, 1.1 Hz, (CH₃)₃CCCCCCC**H**), 7.34 (1H, tt, *J* = 7.6, 1.1 Hz, C**H**CHCCO), 3.19-3.22 (2H, m, C**H₂**CH₂CO), 2.40-2.72 (2H, m, CH₂C**H₂**CO), 1.31 (9H, s, (C**H₃**)₃). ^{13}C NMR (126 MHz, $CDCl_3$) δ_C 206.5 (**C**=O), 158.4 (**C**CO), 138.1 ((CH₃)₃CCCCCC**C**H), 137.5 ((CH₃)₃CCCCC**C**), 127.7 (**C**HCHCCO), 124.0 (**C**HCCO), 121.4 (**C**CH₂CH₂CO), 94.1 ((CH₃)₃C**C**), 79.4 (alkyne **C**), 72.3

(($(CH_3)_3$CCCC**C**), 63.7 (alkyne **C**), 36.2 (CH_2**C**H_2CO), 30.6 ((**C**H_3)$_3$), 28.5 (($(CH_3)_3$**C**), 25.7 (**C**H_2CH_2CO). HRMS (ESI$^+$): Exact mass calculated for $C_{17}H_{17}O^+$ [M+H]$^+$: 237.1279, found: 237.1268.

(4-Bromo-1,2-phenylene)dimethanol (50)

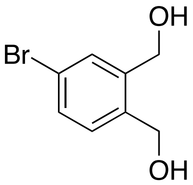

Zinc chloride (880 mg, 6.50 mmol) was added to a suspension of lithium aluminium hydride (2.0 M in THF, 12.8 mL, 25.8 mmol) in dry THF (80 mL) at 0 ^{o}C. A solution of 4-bromophthalic anhydride **49** (2.5 g, 10.8 mmol) in dry THF (12.5 mL) was added and the reaction was warmed to room temperature and stirred for 18 hours. The reaction was cooled to 0 ^{o}C. Water (25 mL) and 10% hydrochloric acid solution (75 mL) were added and the aqueous phase was extracted with ethyl acetate (3 x 50 mL). The combined organic layers were washed with brine, dried over magnesium sulfate, filtered and concentrated in vacuo to give the crude product, which was purified by column chromatography (1:1 petroleum ether/ethyl acetate to 100% ethyl acetate) to give the purified product **50** as a white solid (1.76 g, 75%). ^{1}H NMR (400 MHz, $CDCl_3$) δ_H 7.50 (1H, d, *J* = 2.1 Hz, CHC**H**CCH_2OH), 7.43 (1H, dd, *J* = 8.0, 2.1 Hz, BrCC**H**CCH_2OH), 7.21 (1H, d, *J* = 8.0 Hz, C**H**CHCCH_2OH), 4.66 (2H, s, C**H$_2$**OH), 4.65 (2H, s, C**H$_2$**OH), 3.12 (1H, s, O**H**), 3.06 (1H, s, O**H**). Data is in agreement with that reported in the literature.[12]

5-Bromo-1,3-dihydroisobenzofuran (52)

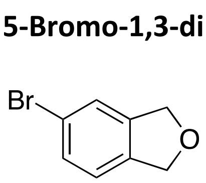

Manganese dioxide (1.08 g, 12.4 mmol) was added to a solution of (4-bromo-1,2-phenylene)dimethanol **50** (538.3 mg, 2.48 mmol) in DCM (43 mL). The reaction was stirred at 40 ^{o}C for 2 hours. Additional manganese dioxide (1.08 g, 12.4 mmol) was added and the mixture was stirred at 40 ^{o}C for a further 40 minutes. The mixture was then filtered through

celite. Triethylsilane (1.19 mL, 7.44 mmol) was added dropwise and the mixture was stirred for 30 minutes, followed by the addition of trifluoroacetic acid (0.949 mL, 12.4 mmol). The reaction mixture was stirred for 24 hours. The reaction mixture was concentrated under vacuum to give the crude product, which was purified by column chromatography (hexane/ethyl acetate, 0% to 20% ethyl acetate) to give the purified product **52** as a white solid (349.8 mg, 71%). ^{1}H NMR (400 MHz, $CDCl_3$) δ_H 7.37-7.41 (2H, m, Ar**H**), 7.11 (1H, dt, *J* = 8.5 Hz, Ar**H**), 5.07-5.09 (2H, m, C$\mathbf{H_2}$), 5.04-5.06 (2H, m, C$\mathbf{H_2}$). Data is in agreement with that reported in the literature.[13]

((1,3-Dihydroisobenzofuran-5-yl)ethynyl)triisopropylsilane (53)

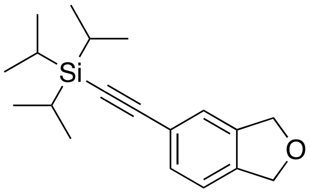

Bis(triphenylphosphine)palladium(II)dichloride (2 mol%, 24.7 mg, 0.0352 mmol) was added to a solution of 5-bromo-1,3-dihydroisobenzofuran **52** (349.8 mg, 1.76 mmol), tri(isopropylsilyl)acetylene **29** (0.632 mL, 2.82 mmol), copper (I) iodide (2 mol%, 6.70 mg, 0.0352 mmol) and triethylamine (4.35 mL) in THF (4.35 mL). The reaction mixture was heated to 80 °C and stirred for 24 h. The reaction was then cooled to room temperature and was passed through a pad of silica gel with an eluant of ethyl acetate. The solvent was removed under reduced pressure to give the crude product, which was purified by column chromatography (0-25% petroleum ether/ethyl acetate) to give purified **53** as a yellow oil (86.9 mg, 16%). IR ν_{max}/cm^{-1} 2941 (C-H), 2862 (C-H), 1047 (C-O), 808. ^{1}H NMR (500 MHz, $CDCl_3$) δ_H 7.39 (1H, d, *J* = 7.8 Hz, Ar**H**), 7.36 (1H, s, CC**H**C), 7.17 (1H, d, *J* = 7.8 Hz, Ar**H**), 5.09 (2H, d, *J* = 1.6 Hz, C$\mathbf{H_2}$), 5.08 (2H, d, *J* = 1.6 Hz, C$\mathbf{H_2}$) 1.12-1.14 (21H, m, SiC**H**, SICH(C$\mathbf{H_3}$)$_2$) . ^{13}C NMR (126 MHz, $CDCl_3$) δ_C 139.5, 139.4, 131.4, 124.7, 122.7, 121.0, 107.1 (SiC**C**C), 90.4 (Si**C**CC), 73.7 (**C**H_2), 73.4 (**C**H_2), 18.8 (6C, (**C**H_3)$_2$C), 11.4 (3C, (CH_3)$_2$**C**). HRMS (ESI$^+$): Exact mass calculated for $C_{19}H_{29}OSi^+$ [M+H]$^+$: 301.1988, found: 301.1972.

5-Ethynyl-1,3-dihydroisobenzofuran (54)

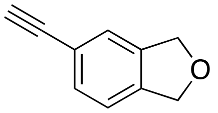

TBAF (1.0 M in THF, 0.376 mL, 0.376 mmol) was added to a solution of ((1,3-dihydroisobenzofuran-5-yl)ethynyl)triisopropylsilane **53** (86.7 mg, 0.289 mmol) in THF (0.7 mL) at 0 ^{o}C and the reaction was stirred for 3 hours. The reaction was quenched with water and warmed to room temperature, the aqueous layer was extracted three times with DCM. The combined organic layers were dried over sodium sulfate, filtered and concentrated under vacuum to give the crude product, which was purified by column chromatography (0-20% petroleum ether/ethyl acetate) to give the purified product **54** as a yellow oil (20.1 mg, 48%). IR v_{max}/cm^{-1} 2862 (C-H), 1771, 1045 (C-O), 808. ^{1}H NMR (500 MHz, $CDCl_3$) δ_H 7.40 (1H, d, *J* = 7.8 Hz, ArH), 7.36 (1H, s, CC**H**C), 7.19 (1H, d, *J* = 7.8 Hz, ArH), 5.10 (2H, d, *J* = 2.4 Hz, C**H$_2$**), 5.09 (2H, d, *J* = 2.4 Hz, C**H$_2$**), 3.06 (1H, s, alkyne H). ^{13}C NMR (126 MHz, $CDCl_3$) δ_C 140.1, 139.6, 131.6, 124.8, 121.3, 121.1, 83.7 (alkyne **C**H), 77.1 (alkyne **C**), 73.6 (**C**H$_2$), 73.4 (**C**H$_2$). HRMS (ESI$^+$): Exact mass calculated for $C_{10}H_8ONH_4^+$ $[M+NH_4]^+$: 162.0913, found: 162.0912.

5-(Bromoethynyl)-1,3-dihydroisobenzofuran (55)

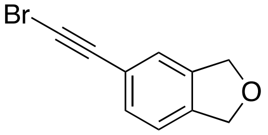

5-Ethynyl-1,3-dihydroisobenzofuran **54** (20.0 mg, 0.139 mmol) was added to a solution of N-bromosuccinimide (29.6 mg, 0.167 mmol) and silver nitrate (2.36 mg, 0.0139 mmol) in acetone (0.404 mL) and the solution was stirred at room temperature for 3 hours. On completion, the solvent was removed under reduced pressure. The resulting residue was dissolved in water and the aqueous layer was extracted with diethyl ether (3 x), the combined organic layers were washed with brine, dried over magnesium sulfate, filtered and concentrated under reduced pressure. The crude product was purified by column chromatography (20% ethyl acetate in petroleum ether) to give the purified product **55** as a yellow oil (16.4 mg, 53%). IR v_{max}/cm^{-1} 2916 (C-H), 2849 (C-H), 1037 (C-O), 815. ^{1}H NMR (500

MHz, $CDCl_3$) δ_H 7.36 (1H, d, *J* = 7.8 Hz, Ar**H**), 7.32 (1H, s, CC**H**C), 7.18 (1H, d, *J* = 7.8 Hz, Ar**H**), 5.08-5.09 (4H, m, C**H₂**). ^{13}C NMR (126 MHz, $CDCl_3$) δ_C 140.0, 139.6, 131.4, 124.7, 121.9, 121.2, 80.0 (alkyne **C**), 73.6 (**C**H_2), 73.4 (**C**H_2), 49.7 (**C**Br). HRMS (EI): Exact mass calculated for $C_{10}H_7{}^{79}BrO$: 221.9675, found: 221.9679.

5-(5,5-Dimethylhexa-1,3-diyn-1-yl)-1,3-dihydroisobenzofuran (18)

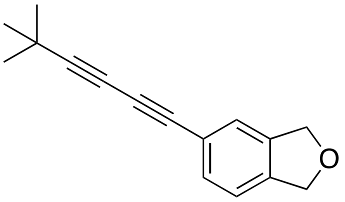

A 30% butylamine/water solution (0.21 mL) was added to an RBF containing copper(I) chloride (1.78 mg, 0.0180 mmol) at 0 °C, hydroxylamine hydrochloride was added until the blue colour disappeared. A solution of 5-(bromoethynyl)-1,3-dihydroisobenzofuran **55** (16.3 mg, 0.0731 mmol) in DCM (0.1 mL) was added, quickly followed by a solution of 3,3-dimethylbut-1-yne **25** (7.50 μL, 0.0601 mmol) in DCM (0.1 mL). The reaction was warmed to room temperature and stirred for 3 hours. On completion, the aqueous layer was extracted with DCM. The organic layer was dried over sodium sulfate, filtered and concentrated under vacuum to give the crude product, which was purified by column chromatography (0 to 20% petroleum ether/ethyl acetate) to give the desired product **18** as a yellow solid (8.4 mg, 65%). m.p. 41-43 °C. IR ν_{max}/cm^{-1} 2964 (C-H), 2922 (C-H), 2862 (C-H), 1047 (C-O), 820. ^1H NMR (500 MHz, $CDCl_3$) δ_H 7.37-7.39 (1H, m, Ar**H**), 7.33 (1H, s, CC**H**C), 7.17 (1H, d, *J* = 7.9 Hz, Ar**H**), 5.08-5.10 (2H, m, C**H₂**), 5.06-5.08 (2H, m, C**H₂**), 1.29 (9H, s, (C**H₃**)₃). HRMS (ESI^+): Exact mass calculated for $C_{16}H_{17}O^+$ $[M+H]^+$: 225.1279, found: 225.1276.

3-Bromoprop-2-yn-1-ol (58)

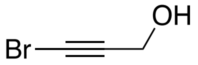

Propargyl alcohol **56** (1.04 mL, 17.8 mmol) was added to a solution of N-bromosuccinimide (3.49 g, 19.6 mmol) and silver nitrate (302 mg, 1.78 mmol) in acetone (30 mL) and the solution was stirred at room temperature for 3 hours. On completion, the solvent was removed under reduced pressure. The resulting residue was dissolved in water and the aqueous layer was extracted three times with diethyl ether. The combined organic layers were washed with brine, dried over magnesium sulfate, filtered and concentrated under reduced pressure. The crude was purified by column chromatography (20% ethyl acetate in petroleum ether) to give the purified product **58** as a yellow oil (16.4 mg, 53%). ^{1}H NMR (500 MHz, $CDCl_3$) δ_H 4.30 (2H, d, *J* = 6.0 Hz, C**H$_2$**), 1.65-1.89 (1H, broad m, O**H**). Data is in agreement with that reported in the literature.[14]

5-(4-(*Tert*-butyl)phenyl)penta-2,4-diyn-1-ol (20)

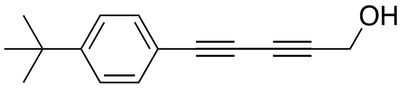

A 30% butylamine/water solution (2.16 mL) was added to an RBF containing copper(I) chloride (18.8 mg, 0.190 mmol) at 0 ^{o}C, hydroxylamine hydrochloride was then added until the blue colour disappeared. A solution of 4-(*tert*-butyl)phenylacetylene **37** (0.112 mL, 0.632 mmol) in DCM (0.82 mL) was added, quickly followed by a solution of 3-bromoprop-2-yn-1-ol **58** (102.4 mg, 0.758 mmol) in DCM (0.82 mL). The reaction was warmed to room temperature and stirred for 3 hours. On completion, the aqueous layer was extracted with DCM. The organic layer was dried over sodium sulfate, filtered and concentrated under vacuum to give the crude product which was purified by column chromatography (0 to 20% petroleum ether/ethyl acetate) to give the purified product **20** as a white solid (88.3 mg, 66%). m.p. 104-106 ^{o}C. IR ν_{max}/cm^{-1} 2947 (C-H), 1325 (O-H), 1117 (O-H), 1016 (O-H), 1009, 825. ^{1}H NMR (500 MHz, $CDCl_3$) δ_H 7.42-7.44 (2H, m, $(CH_3)_3$CCC**H**CH), 7.33-7.36 (2H, m, $(CH_3)_3$CCCHC**H**), 4.42 (1H, s, C**H**OH), 1.30 (9H, s, (C**H$_3$**)$_3$). ^{13}C NMR (126 MHz, $CDCl_3$) δ_C 153.0 ($(CH_3)_3$C**C**), 132.5 (2C, $(CH_3)_3$CC**C**HCH), 125.6 (2C, $(CH_3)_3$CCCH**C**H), 118.4 (CHOHCCCC**C**), 80.2 (**C**CHOH), 79.1 (CHC**C**C),

72.7 (alkyne C), 70.8 (alkyne C), 51.9 (**C**H_2OH), 35.1 ((CH_3)$_3$**C**), 31.2 (3C, (**C**H_3)$_3$). HRMS (ESI$^+$): Exact mass calculated for $C_{15}H_{16}ONa^+$ [M+Na]$^+$: 235.1099, found: 235.1093.

4-Bromo-2-methylbut-3-yn-2-ol (59)

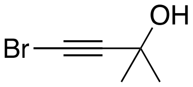

2-Methylbut-3-yn-2-ol **57** (1.15 mL, 11.9 mmol) was added to a solution of N-bromosuccinimide (2.33 g, 13.1 mmol) and silver nitrate (202.1 mg, 1.19 mmol) in acetone (20 mL) and the solution was stirred at room temperature for 3 hours. On completion, the solvent was removed under reduced pressure. The resulting residue was dissolved in water and the aqueous layer was extracted three times with diethyl ether. The combined organic layers were washed with brine, dried over magnesium sulfate, filtered and concentrated under reduced pressure. The crude was purified by column chromatography (20% ethyl acetate in petroleum ether) to give the purified product **59** as a colourless oil (1.38 g, 60%). ^{1}H NMR (500 MHz, $CDCl_3$) δ_H 2.06 (1H, broad s, O**H**), 1.65-1.89 (6H, s, C**H_3**). Data is in agreement with that reported in the literature.[15]

6-(4-(*Tert*-butyl)phenyl)-2-methylhexa-3,5-diyn-2-ol (22)

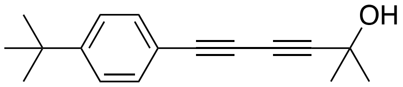

A 30% butylamine/water solution (2.16 mL) was added to an RBF containing copper(I) chloride (18.8 mg, 0.190 mmol) at 0 ^oC, hydroxylamine hydrochloride was then added until the blue colour disappeared. A solution of 4-(*tert*-butyl)phenylacetylene **37** (0.112 mL, 0.632 mmol) in DCM (0.82 mL) was added, quickly followed by a solution of 4-bromo-2-methylbut-3-yn-2-ol **59** (123.6 mg, 0.758 mmol) in DCM (0.82 mL). The reaction was warmed to room temperature and stirred for 3 hours. On completion, the aqueous layer was extracted with DCM. The organic layer was dried over sodium sulfate, filtered and concentrated under vacuum to give the crude product. The crude was purified by column chromatography (0 to 20% petroleum ether/ethyl acetate) to give the purified product **22** as a white solid (107.8 mg, 71%). m.p.

110-112 ^{o}C. IR ν_{max}/cm^{-1} 2970 (C-H), 2901 (C-H), 2361, 1363 (O-H), 1066 (O-H), 839. ^{1}H NMR (500 MHz, $CDCl_3$) δ_H 7.41-7.43 (2H, m, $(CH_3)_3$CCC**H**CH), 7.33-7.35 (2H, m, $(CH_3)_3$CCCHC**H**), 1.94 (1H, s, O**H**), 1.58 (6H, s, (C**H$_3$**)$_2$), 1.31 (9H, s, (C**H$_3$**)$_3$). ^{13}C NMR (126 MHz, $CDCl_3$) δ_C 151.9 ($(CH_3)_3$C**C**), 131.5 (2C, $(CH_3)_3$CC**C**HCH), 124.6 (2C, $(CH_3)_3$CCCH**C**H), 117.6 (CHOHCCCC**C**), 85.4 (**C**COH), 78.3 (CHC**C**C), 71.7 (**C**CCCOH), 66.4 (**C**CCOH), 64.9 ((**C**H$_2$)$_2$COH), 35.1 ($(CH_3)_3$**C**), 31.3 (2C, C(**C**H$_3$)$_2$), 31.3 (3C, (**C**H$_3$)$_3$). HRMS (ESI$^+$): Exact mass calculated for $C_{17}H_{20}ONa^+$ [M+Na]$^+$: 263.1412, found: 263.1406.

(*R*)-4-Bromobut-3-yn-2-ol ((*R*)-36)

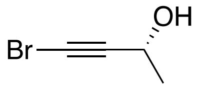

(*R*)-(-)-3-Butyn-2-ol **(*R*)-27** (0.559 mL, 7.13 mmol) was added to a solution of N-bromosuccinimide (1.40 g, 7.85 mmol) and silver nitrate (121.2 mg, 0.713 mmol) in acetone (12 mL) and the solution was stirred at room temperature for 3 hours. On completion, the solvent was removed under reduced pressure. The resulting residue was dissolved in water (3 mL) and the aqueous layer was extracted with diethyl ether (3 x 3 mL). The combined organic layers were washed with brine (5 mL), dried over magnesium sulfate, filtered and concentrated under reduced pressure. The crude product was purified by column chromatography (20% ethyl acetate in petroleum ether) to give the purified product **(*R*)-36** as a yellow liquid (734.4 mg, 69%). IR ν_{max}/cm^{-1} 2984 (C-H), 1373 (O-H), 1265, 1119 (C-O), 1043 (C-O). ^{1}H NMR (400 MHz, $CDCl_3$) δ_H 4.55 (1H, qd, *J* = 6.6, 5.4 Hz, C**H**OH), 1.46 (3H, dd, *J* = 6.6 Hz, C**H$_3$**). HRMS (ESI$^+$): Exact mass calculated for $C_4H_6{}^{79}BrO^+$: 148.9597, found: 148.9590. $[\alpha]^{20}_D$ = +23.1^{o} (c = 0.0167, $CHCl_3$).

(*R*)-6-(4-(*Tert*-butyl)phenyl)hexa-3,5-diyn-2-ol ((*R*)-21)

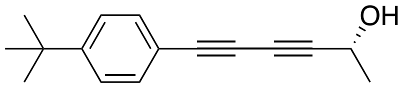

A 30% butylamine/water solution (2.16 mL) was added to an RBF containing copper(I) chloride (18.8 mg, 0.190 mmol) at 0 ^{o}C, hydroxylamine hydrochloride was then added until the blue

colour disappeared. A solution of 4-(*tert*-butyl)phenylacetylene **37** (0.112 mL, 0.632 mmol) in DCM (0.82 mL) was added, quickly followed by a solution of (*R*)-4-bromobut-3-yn-2-ol **(*R*)-36** (113.0 mg, 0.758 mmol) in DCM (0.82 mL). The reaction was warmed to room temperature and stirred for 3 hours. On completion, the aqueous layer was extracted with DCM. The organic layer was dried over sodium sulfate, filtered and concentrated under vacuum to give the crude product, which was purified by column chromatography (0 to 20% petroleum ether/ethyl acetate) to give the purified product **(*R*)-21** as a white solid (98.4 mg, 69%). m.p. 81-83 ^{o}C. IR ν_{max}/cm^{-1} 3297, 2957 (C-H), 1069 (O-H), 837. ^{1}H NMR (500 MHz, $CDCl_3$) δ_H 7.41-7.43 (2H, m, $(CH_3)_3$CCC**H**CH), 7.33-7.35 (2H, m, $(CH_3)_3$CCCHC**H**), 4.63-4.70 (1H, m, C**H**OH), 1.52 (3H, d, *J* = 6.6 Hz, C**H$_3$**), 1.30 (9H, s, (C**H$_3$**)$_3$). ^{13}C NMR (126 MHz, $CDCl_3$) δ_C 152.9 ($(CH_3)_3$C**C**), 132.5 (2C, $(CH_3)_3$CC**C**HCH), 125.6 (2C, $(CH_3)_3$CCCH**C**H), 118.3 (CHOHCCCC**C**), 83.7 (**C**CHOH), 79.2 (CHC**C**C), 72.6 (alkyne C), 69.2 (alkyne C), 59.1 (**C**HOH), 34.9 ($(CH_3)_3$**C**), 31.2 (3C, (C**H$_3$**)$_3$), 24.2 (**C**H$_3$). HRMS (ESI$^+$): Exact mass calculated for $C_{16}H_{18}ONa^+$ [M+Na]$^+$: 249.1250, found: 249.1250. $[\alpha]^{20}_D$ = +15.9^{o} (c = 0.0076, $CHCl_3$). Chiral HPLC analysis: Chiralcel OJ-H (94:6 hexane : IPA, flow rate 1 mL/min^{-1}, temperature 30 °C; detection: 211 nm, t_R: 17.4 min, **99.4 : 0.6 er.**

(*S*)-4-Bromobut-3-yn-2-ol ((*S*)-36)

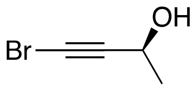

(*S*)-(-)-3-Butyn-2-ol **(*S*)-27** (0.559 mL, 7.13 mmol) was added to a solution of N-bromosuccinimide (1.40 g, 7.85 mmol) and silver nitrate (121.2 mg, 0.713 mmol) in acetone (12 mL) and the solution was stirred at room temperature for 3 hours. On completion, the solvent was removed under reduced pressure. The resulting residue was dissolved in water (3 mL) and the aqueous layer was extracted with diethyl ether (3 x 3 mL). The combined organic layers were washed with brine (5 mL), dried over magnesium sulfate, filtered and concentrated under reduced pressure. The crude was purified by column chromatography (20% ethyl acetate in petroleum ether) to give the purified product **(*S*)-36** as a yellow liquid (616.7 mg, 58%). IR ν_{max}/cm^{-1} 2981 (C-H), 1371 (O-H), 1234, 1128 (C-O), 1069 (C-O). ^{1}H NMR (500 MHz, $CDCl_3$) δ_H 4.44-4.50 (1H, m, C**H**OH), 1.40 (3H, dd, *J* = 6.6, 2.5 Hz, C**H$_3$**). HRMS (ESI$^+$):

Exact mass calculated for $C_4H_6{}^{79}BrO^+$: 148.9597, found: 148.9598. $[\alpha]^{20}_D$ = -24.1° (c = 0.0975, $CHCl_3$).

(*S*)-6-(4-(*Tert*-butyl)phenyl)hexa-3,5-diyn-2-ol ((*S*)-21)

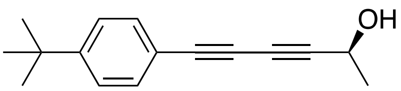

A 30% butylamine/water solution (2.16 mL) was added to an RBF containing copper(I) chloride (18.8 mg, 0.190 mmol) at 0 °C, hydroxylamine hydrochloride was then added until the blue colour disappeared. A solution of 4-(*tert*-butyl)phenylacetylene **37** (0.112 mL, 0.632 mmol) in DCM (0.82 mL) was added, quickly followed by a solution of (*S*)-4-bromobut-3-yn-2-ol **(*S*)-36** (113.0 mg, 0.758 mmol) in DCM (0.82 mL). The reaction was warmed to room temperature and stirred for 3 hours. On completion, the aqueous layer was extracted with DCM, the organic layer was dried over sodium sulfate, filtered and concentrated under vacuum to give the crude product. The crude product was purified by column chromatography (0 to 20% petroleum ether/ethyl acetate) to give the purified product **(*S*)-21** as a white solid (196.0 mg, 73%). m.p. 78-80 °C. IR v_{max}/cm^{-1} 2963 (C-H), 1363, 1267 (O-H), 1132 (O-H), 1016 (O-H), 835. ^{1}H NMR (500 MHz, $CDCl_3$) δ_H 7.41-7.44 (2H, m, $(CH_3)_3$CCC**H**CH), 7.33-7.36 (2H, m, $(CH_3)_3$CCCHC**H**), 4.66 (1H, q, *J* = 6.5 Hz, C**H**OH), 1.52 (3H, d, *J* = 6.5 Hz, C**H$_3$**), 1.30 (9H, s, (C**H$_3$**)$_3$). ^{13}C NMR (126 MHz, $CDCl_3$) δ_C 153.0 (($CH_3)_3$C**C**), 132.5 (2C, $(CH_3)_3$CC**C**HCH), 125.6 (2C, $(CH_3)_3$CCCHC**H**), 118.5 (CHOHCCCC**C**), 83.7 (**C**CHOH), 79.2 (CHC**C**C), 72.6 (alkyne C), 69.2 (alkyne C), 59.1 (**C**HOH), 35.1 (($CH_3)_3$**C**), 31.2 (3C, (C**H$_3$**)$_3$), 24.1 (**C**H$_3$). HRMS (ESI$^+$): Exact mass calculated for $C_{16}H_{18}ONa^+$ $[M+Na]^+$: 249.1250, found: 249.1250. $[\alpha]^{20}_D$ = -7.6° (c = 0.05, $CHCl_3$). Chiral HPLC analysis: Chiralcel OJ-H (94:6 hexane : IPA, flow rate 1 mL/min^{-1}, temperature 30 °C; detection: 211 nm, t_R: 20.6 min, 98.7 : 1.3 er.

Chiral HPLC analysis of (R)-21 and (S)-21

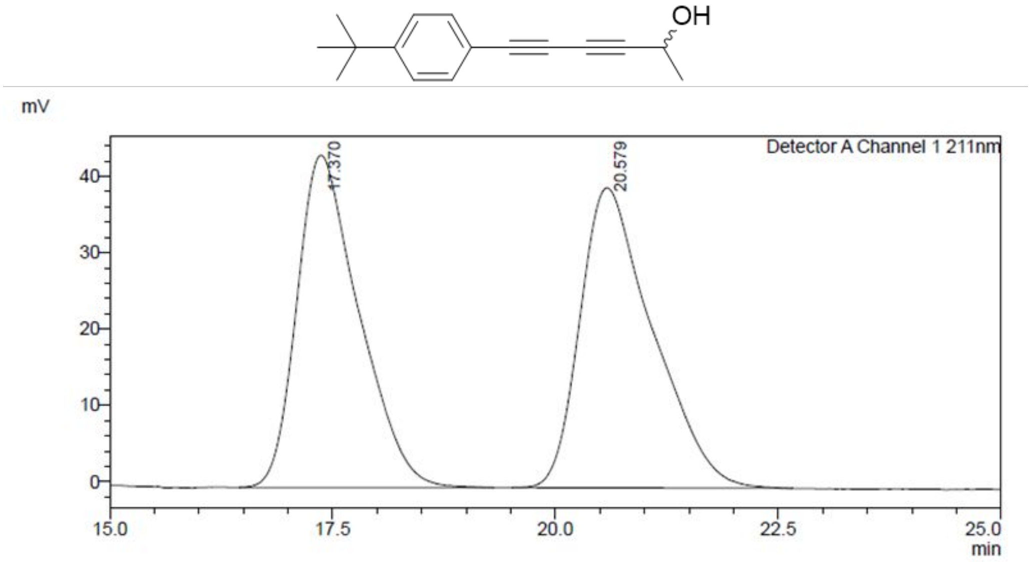

<Peak Table>

Detector A Channel 1 211nm

Peak#	Ret. Time	Area%
1	17.370	48.534
2	20.579	51.466
Total		100.000

Figure S1. Chiral HPLC (Chiralcel OJ-H) analysis of 1:1 add mixed (*S* + *R*)-**21**.

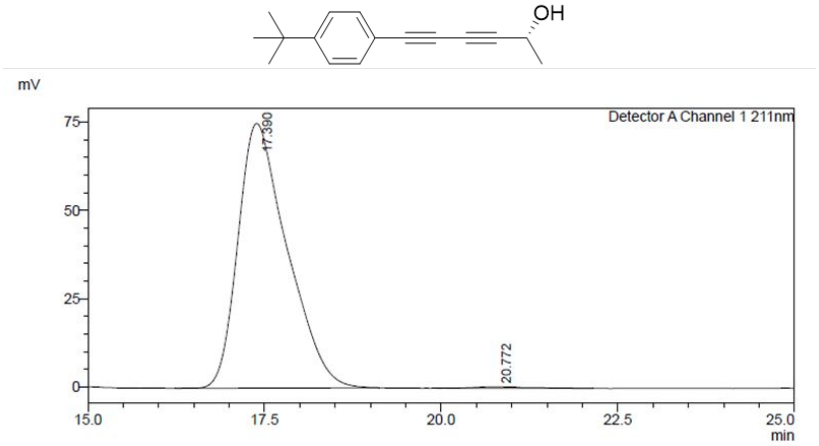

<Peak Table>

Detector A Channel 1 211nm

Peak#	Ret. Time	Area%
1	17.390	99.385
2	20.772	0.615
Total		100.000

Figure S2. Chiral HPLC (Chiralcel OJ-H) analysis of (*R*)-**21**.

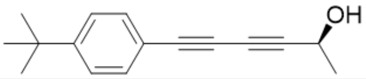

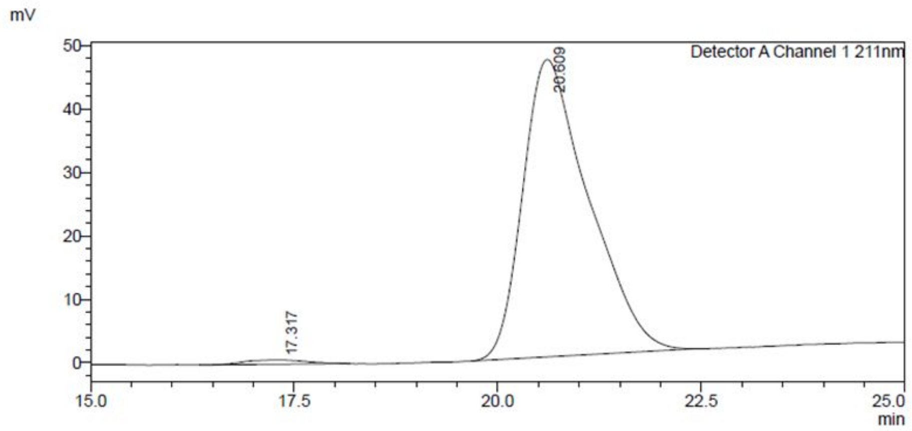

<Peak Table>

Detector A Channel 1 211nm

Peak#	Ret. Time	Area%
1	17.317	1.337
2	20.609	98.663
Total		100.000

Figure S3. Chiral HPLC (Chiralcel OJ-H) analysis of (*S*)-**21**.

NMR spectra of selected compounds

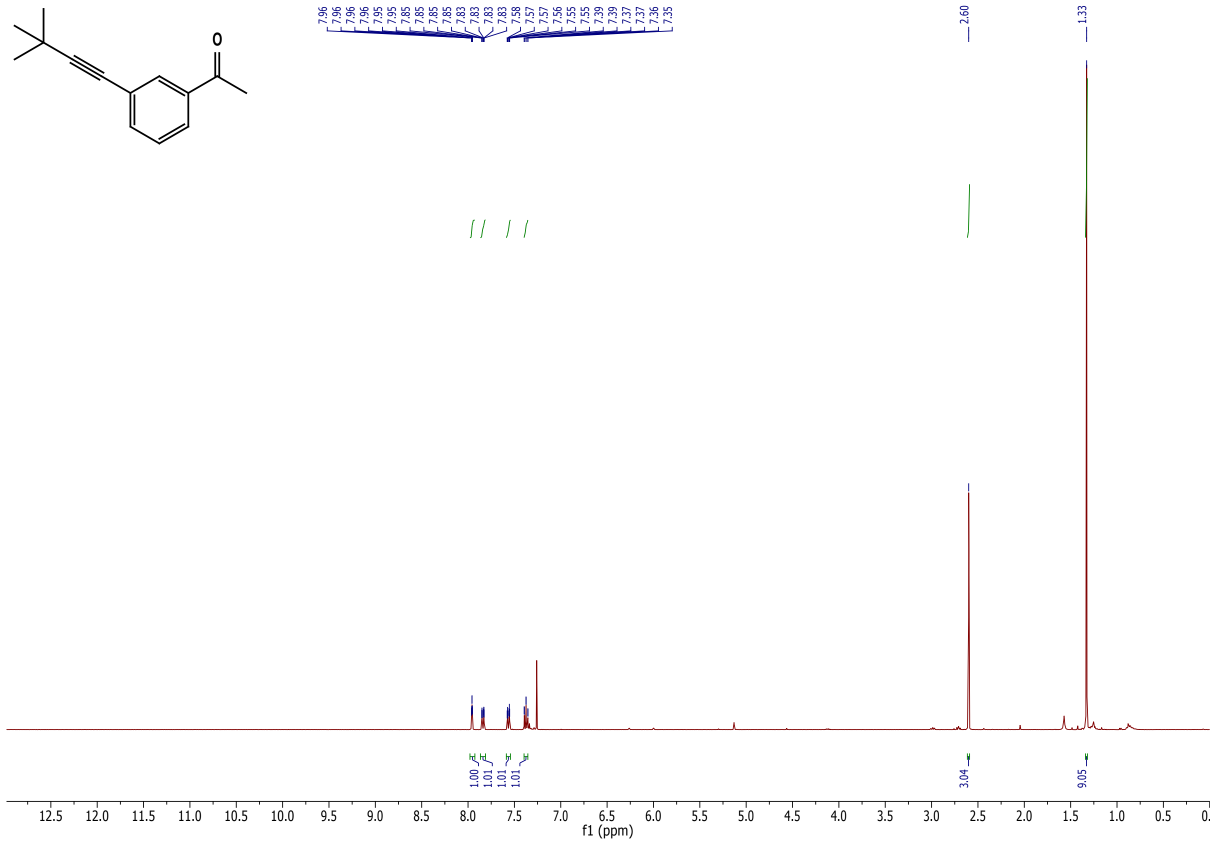

^{1}H NMR spectrum (400.1 MHz, $CDCl_3$) of 1-(3-(3,3-Dimethylbut-1-yn-1-yl)phenyl)ethan-1-one (**11**)

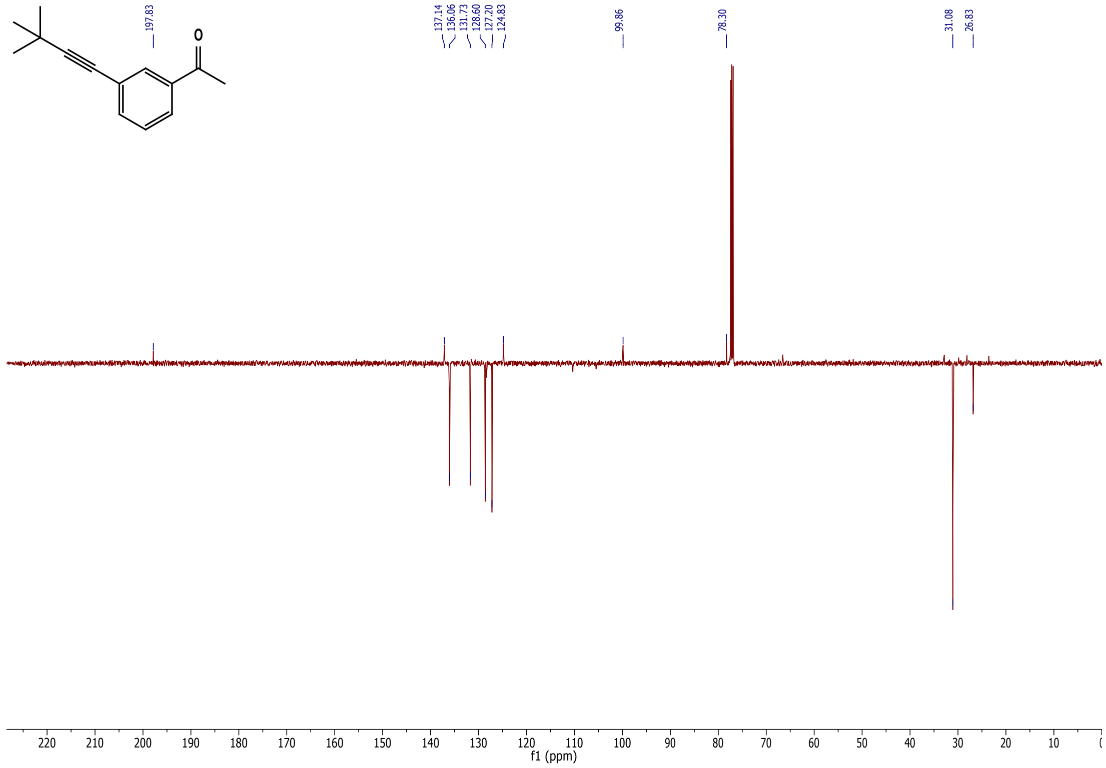

^{13}C NMR spectrum (400.1 MHz, $CDCl_3$) of 1-(3-(3,3-Dimethylbut-1-yn-1-yl)phenyl)ethan-1-one (**11**)

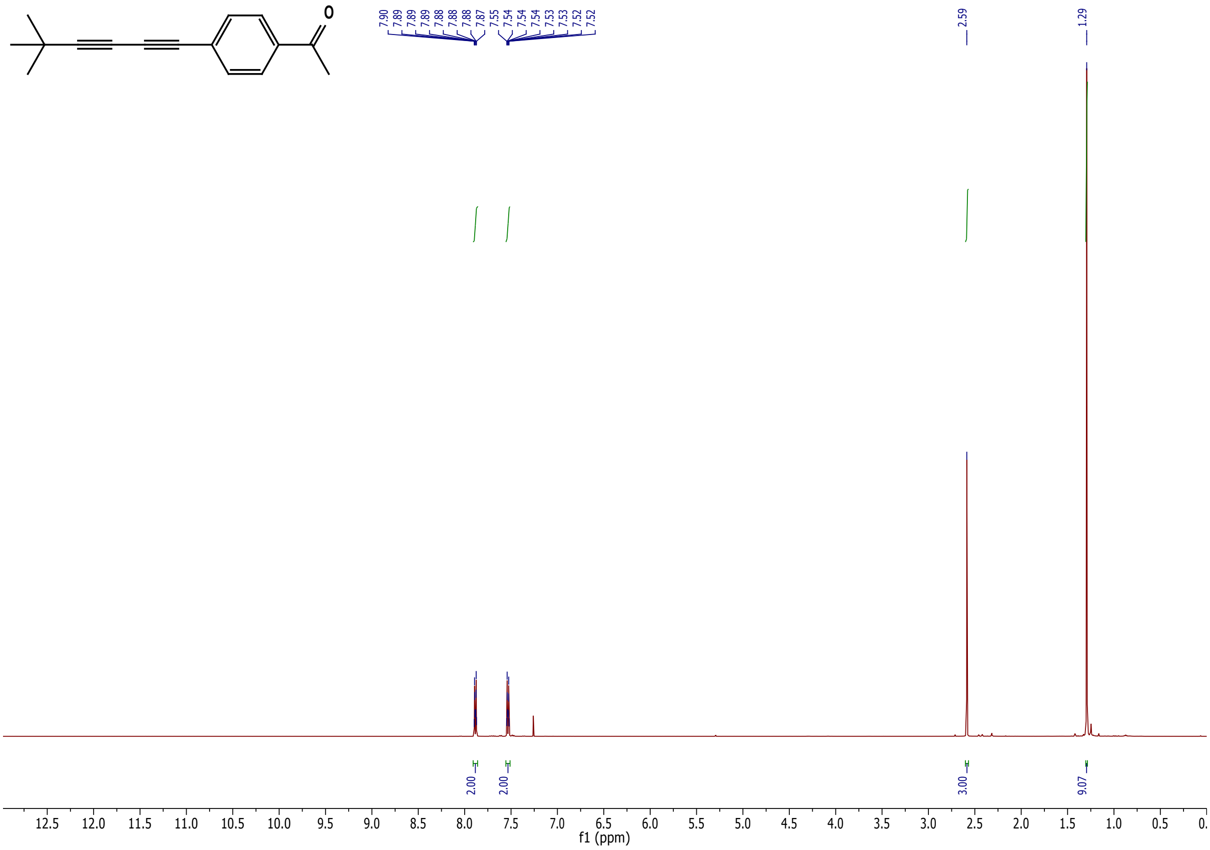

^{1}H NMR spectrum (500.1 MHz, $CDCl_3$) of 1-(4-(5,5-Dimethylhexa-1,3-diyn-1-yl)phenyl)ethan-1-one (**13**)

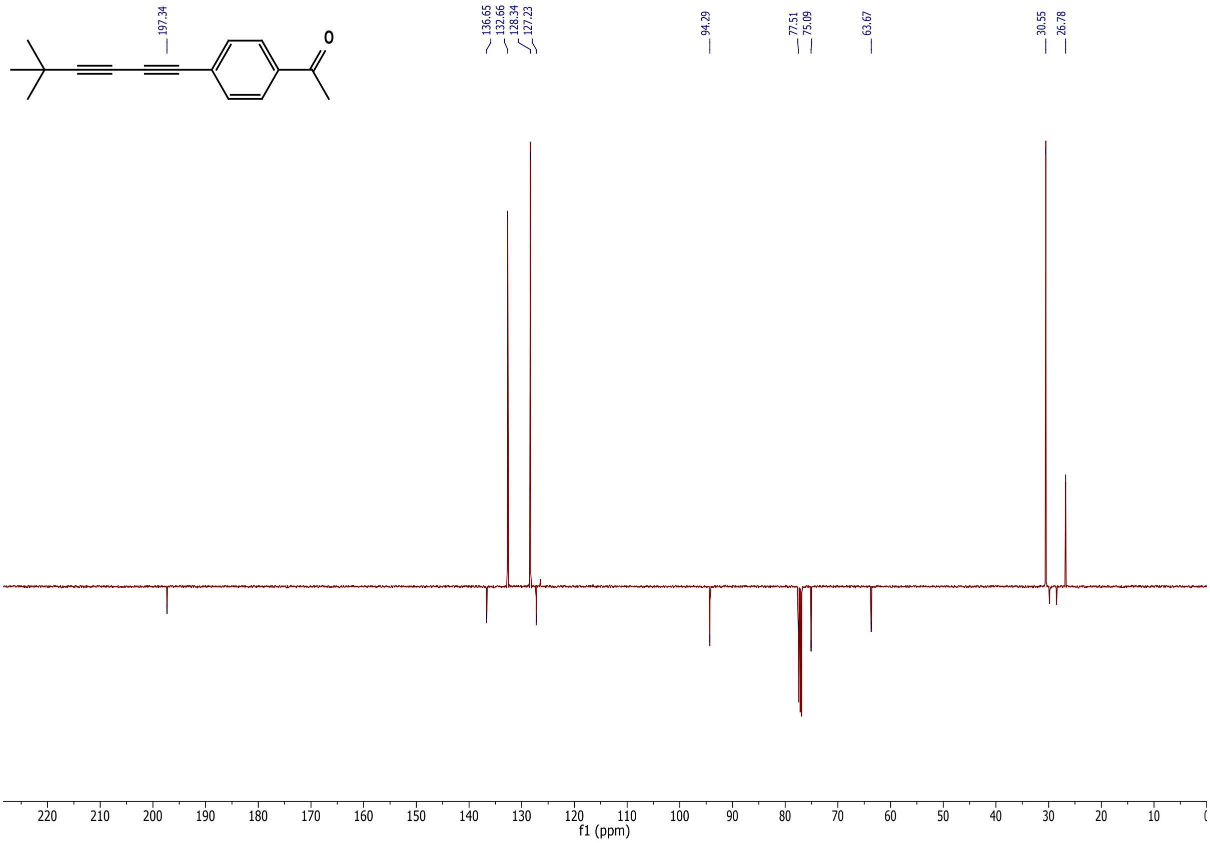

^{13}C NMR spectrum (126 MHz, $CDCl_3$) of 1-(4-(5,5-Dimethylhexa-1,3-diyn-1-yl)phenyl)ethan-1-one (**13**)

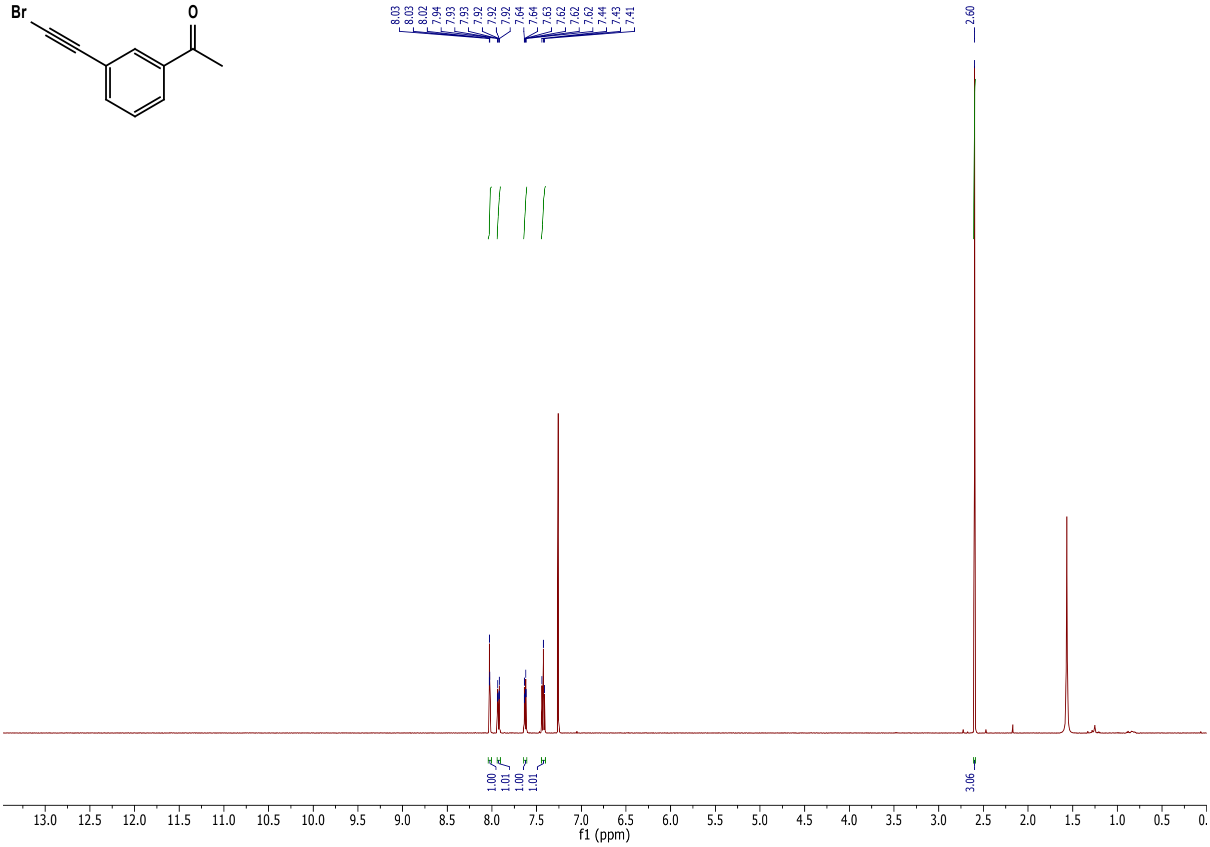

^{1}H NMR spectrum (500.1 MHz, $CDCl_3$) of 1-(3-(Bromoethynyl)phenyl)ethan-1-one (**35**)

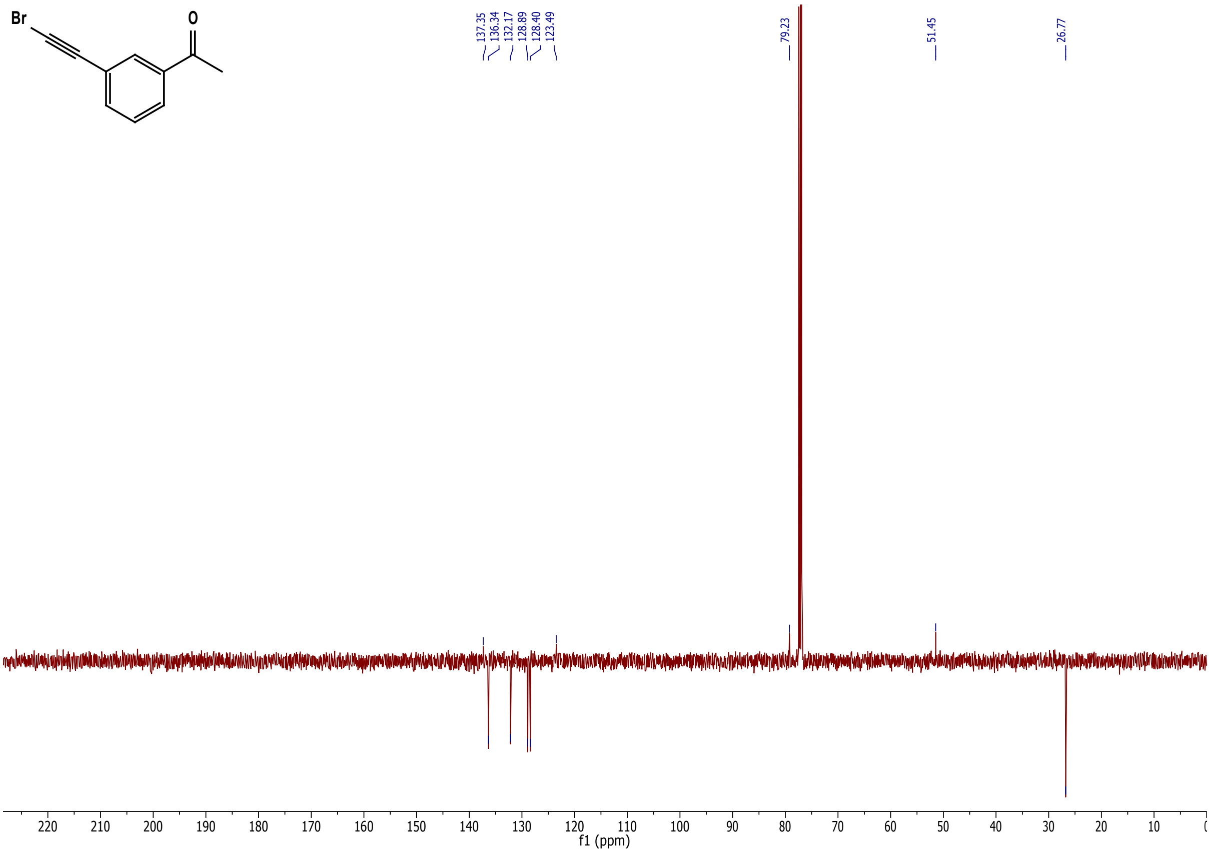

^{13}C NMR spectrum (126 MHz, $CDCl_3$) of 1-(3-(Bromoethynyl)phenyl)ethan-1-one (**35**)

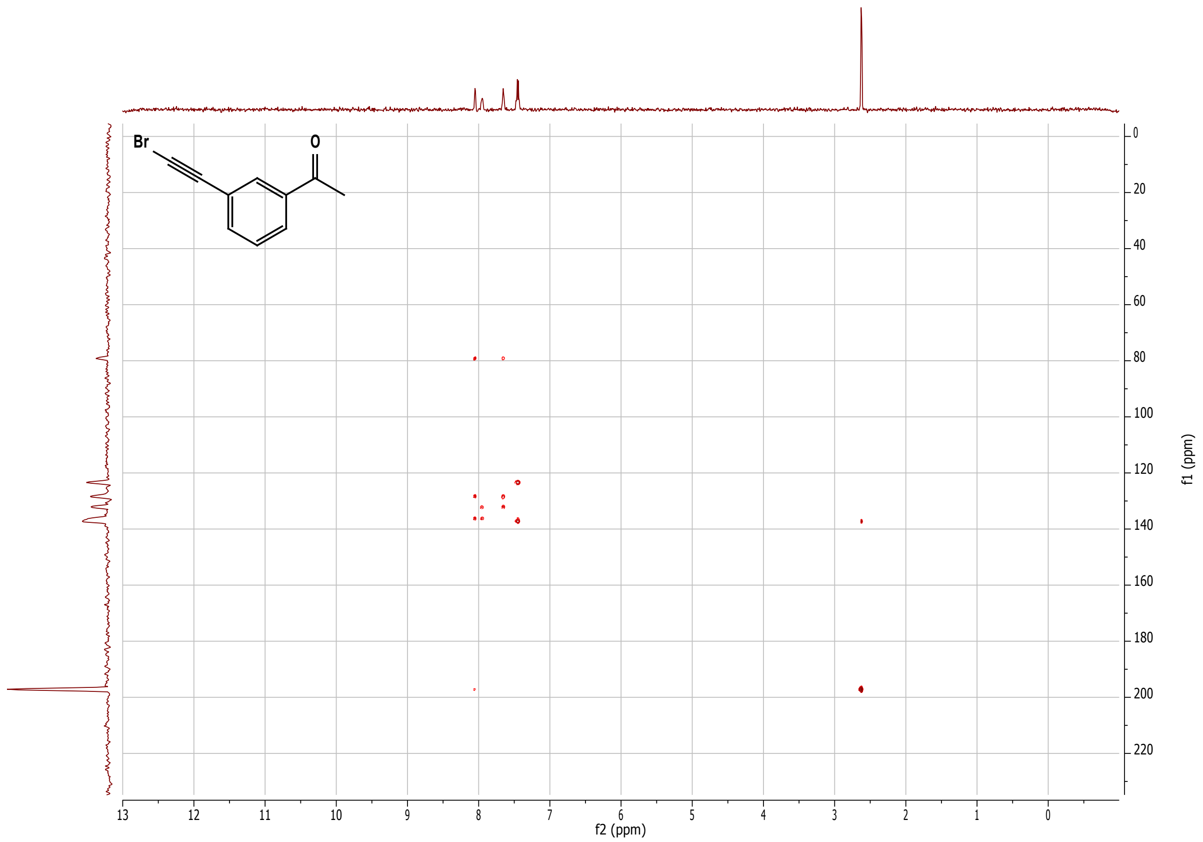

2D $^{1}H,^{13}C$ HMBC NMR spectrum (126 MHz, $CDCl_3$) of 1-(3-(Bromoethynyl)phenyl)ethan-1-one (**35**)

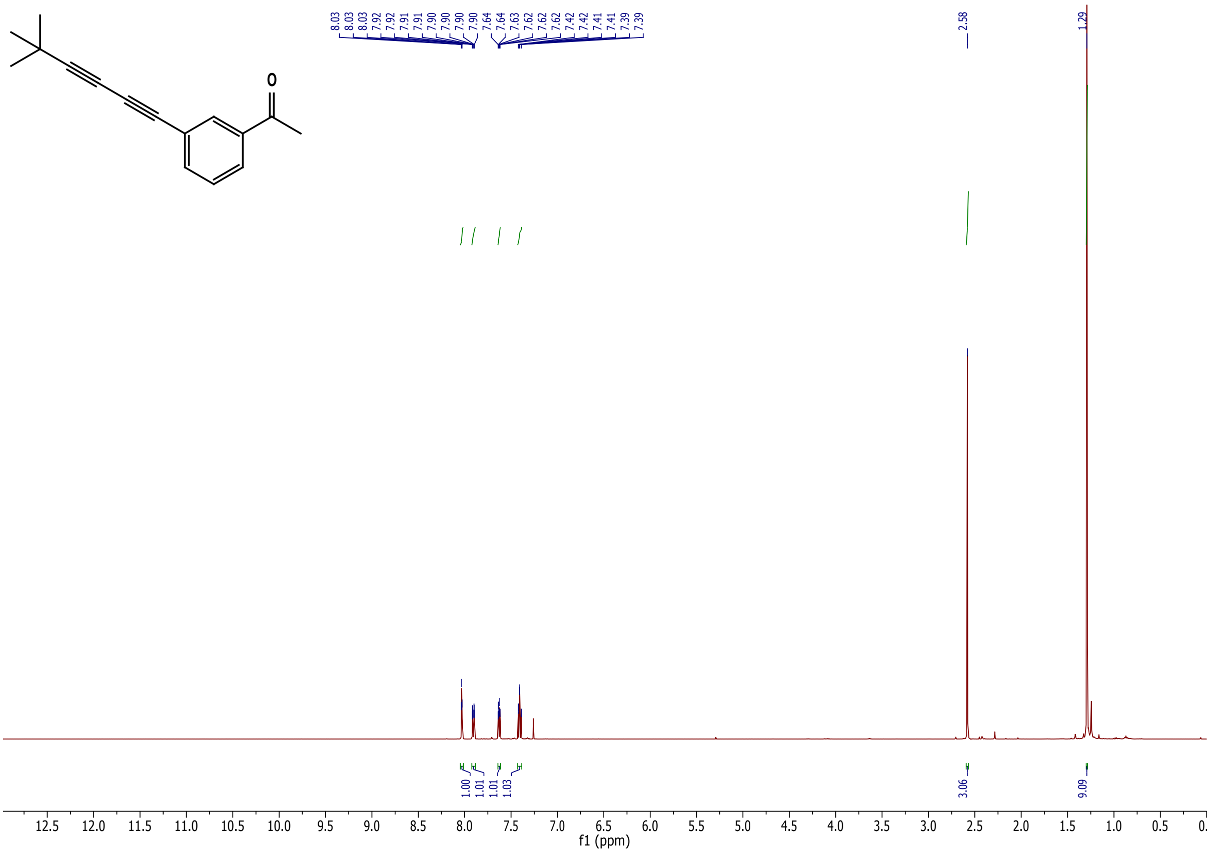

^{1}H NMR spectrum (500.1 MHz, $CDCl_3$) of 1-(3-(5,5-Dimethylhexa-1,3-diyn-1-yl)phenyl)ethan-1-one (**14**)

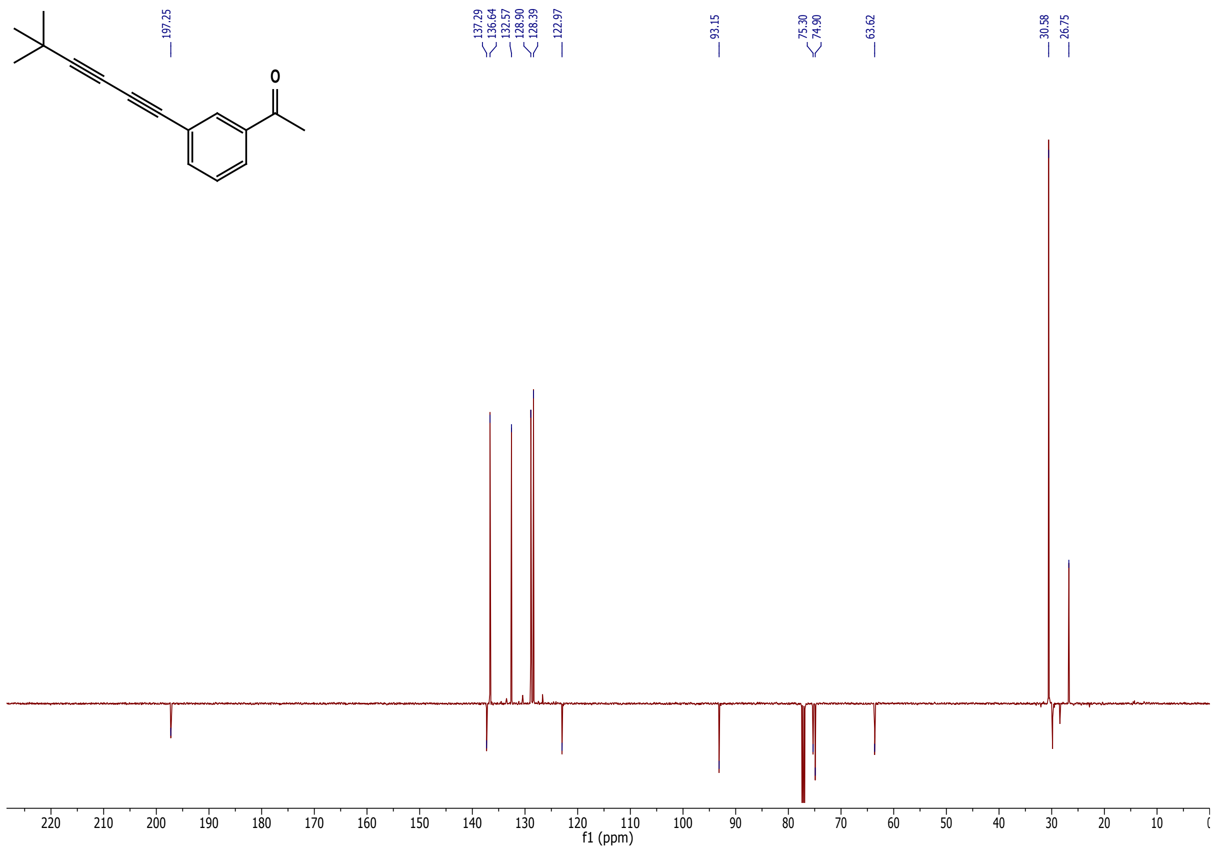

^{13}C NMR spectrum (126 MHz, $CDCl_3$) of 1-(3-(5,5-Dimethylhexa-1,3-diyn-1-yl)phenyl)ethan-1-one (**14**)

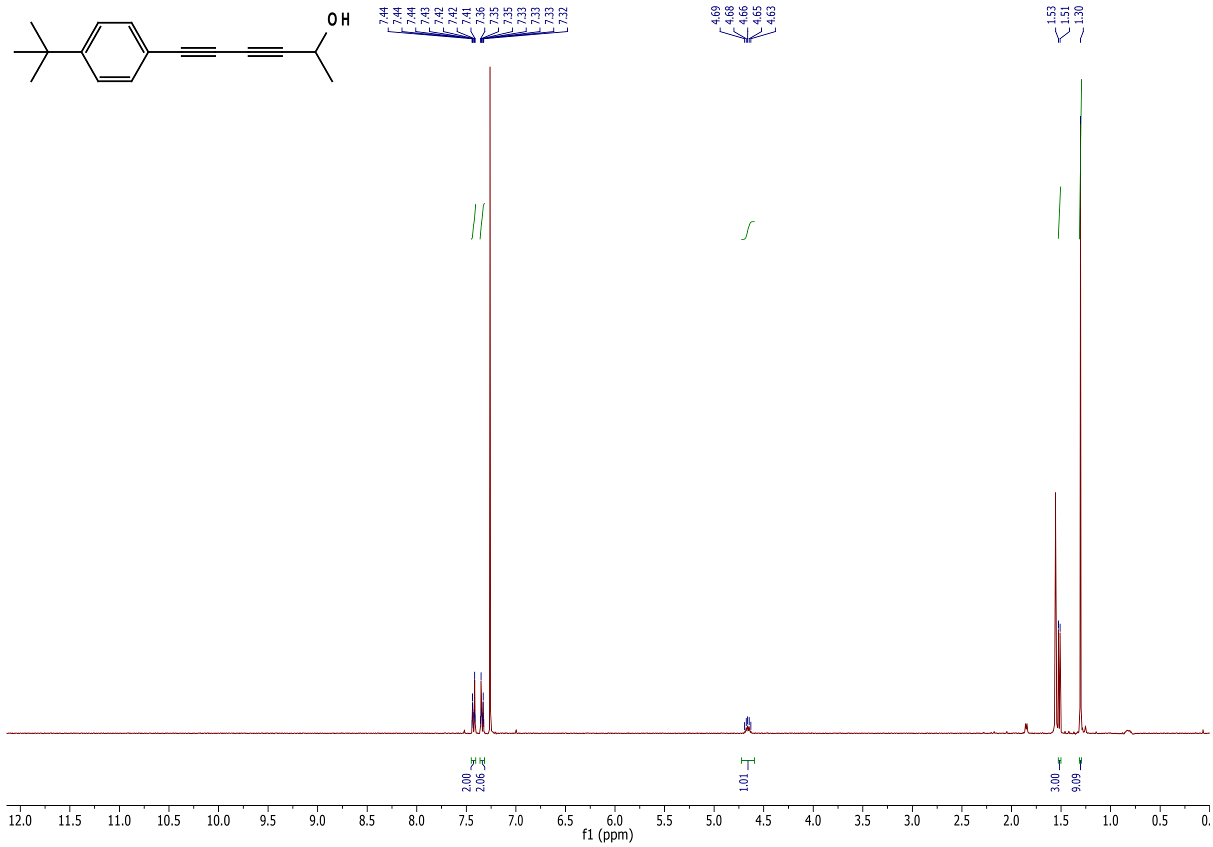

^{1}H NMR spectrum (500.1 MHz, $CDCl_3$) of 6-(4-(*tert*-Butyl)phenyl)hexa-3,5-diyn-2-ol (**21**)

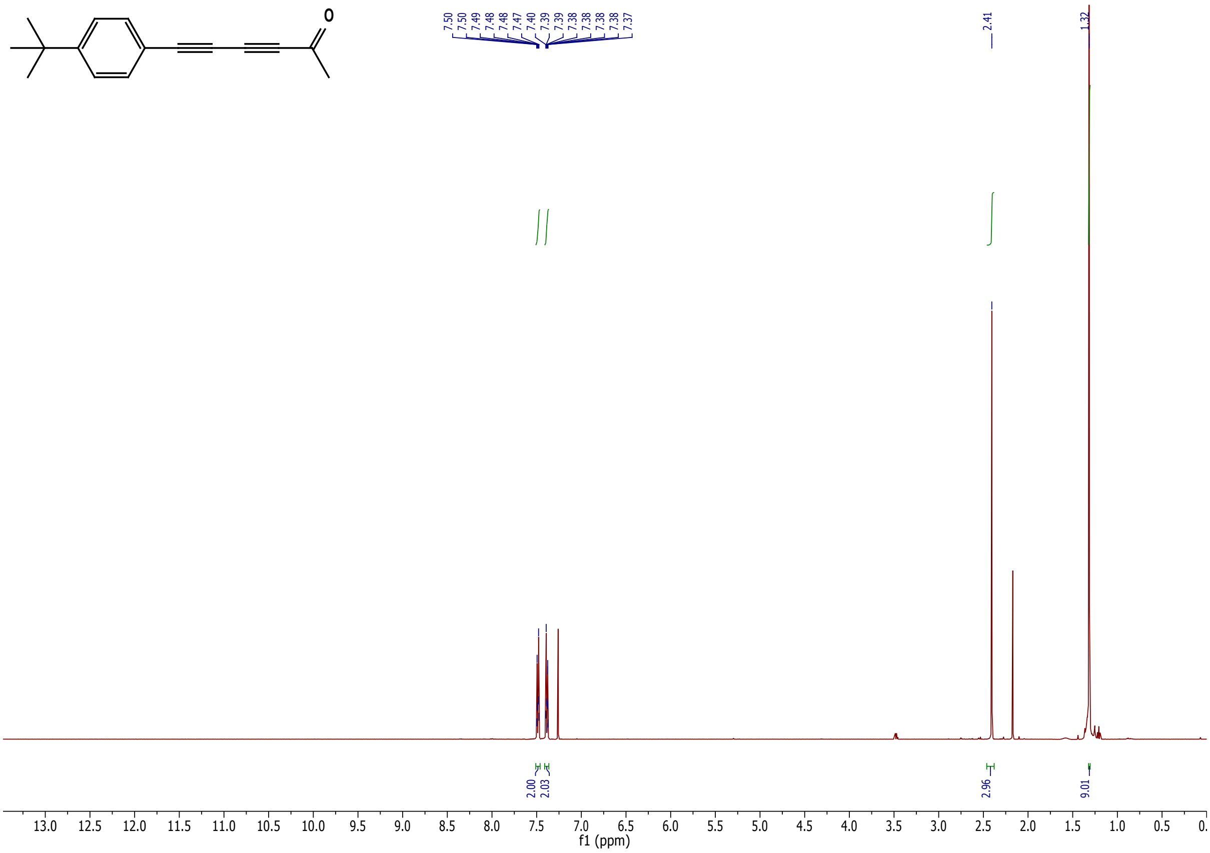

^{1}H NMR spectrum (500.1 MHz, $CDCl_3$) of 6-(4-(*tert*-Butyl)phenyl)hexa-3,5-diyn-2-one (**15**)

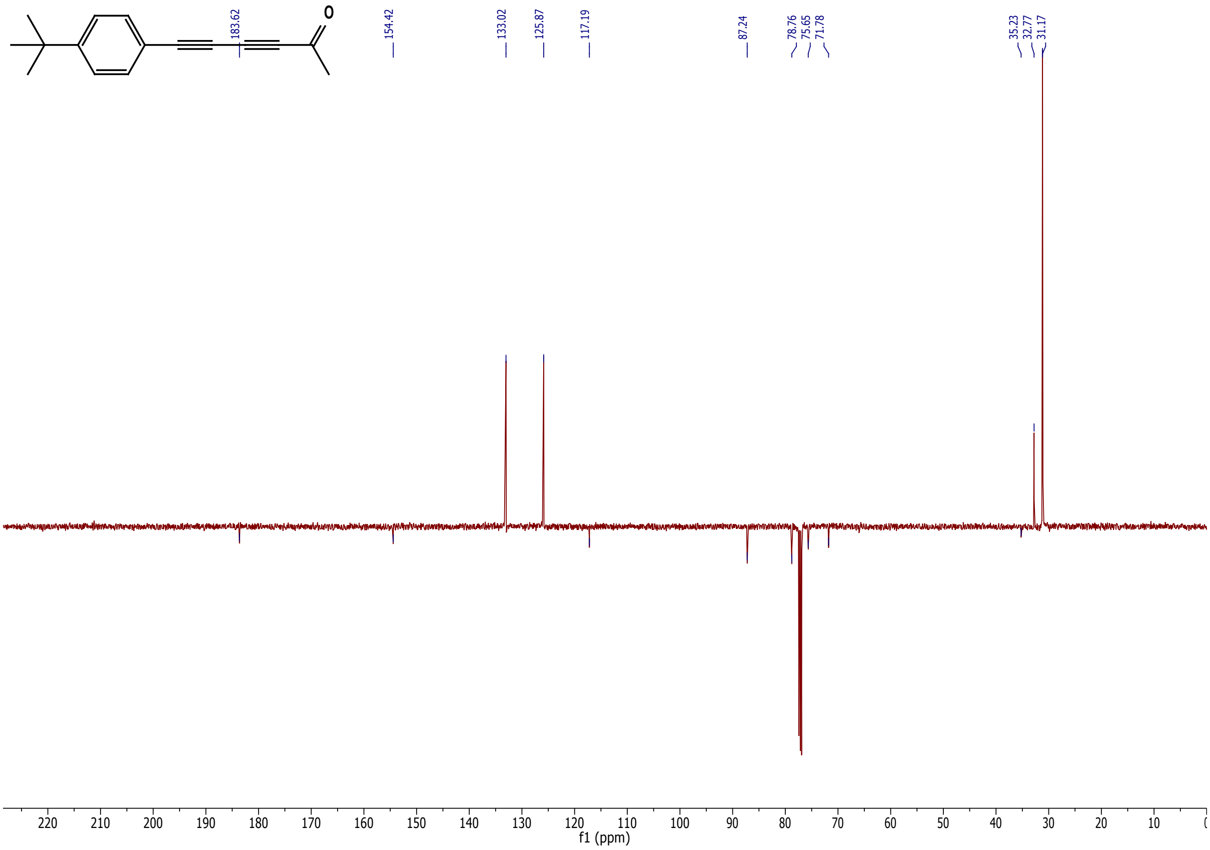

^{13}C NMR spectrum (126 MHz, $CDCl_3$) of 6-(4-(*tert*-Butyl)phenyl)hexa-3,5-diyn-2-one (**15**)

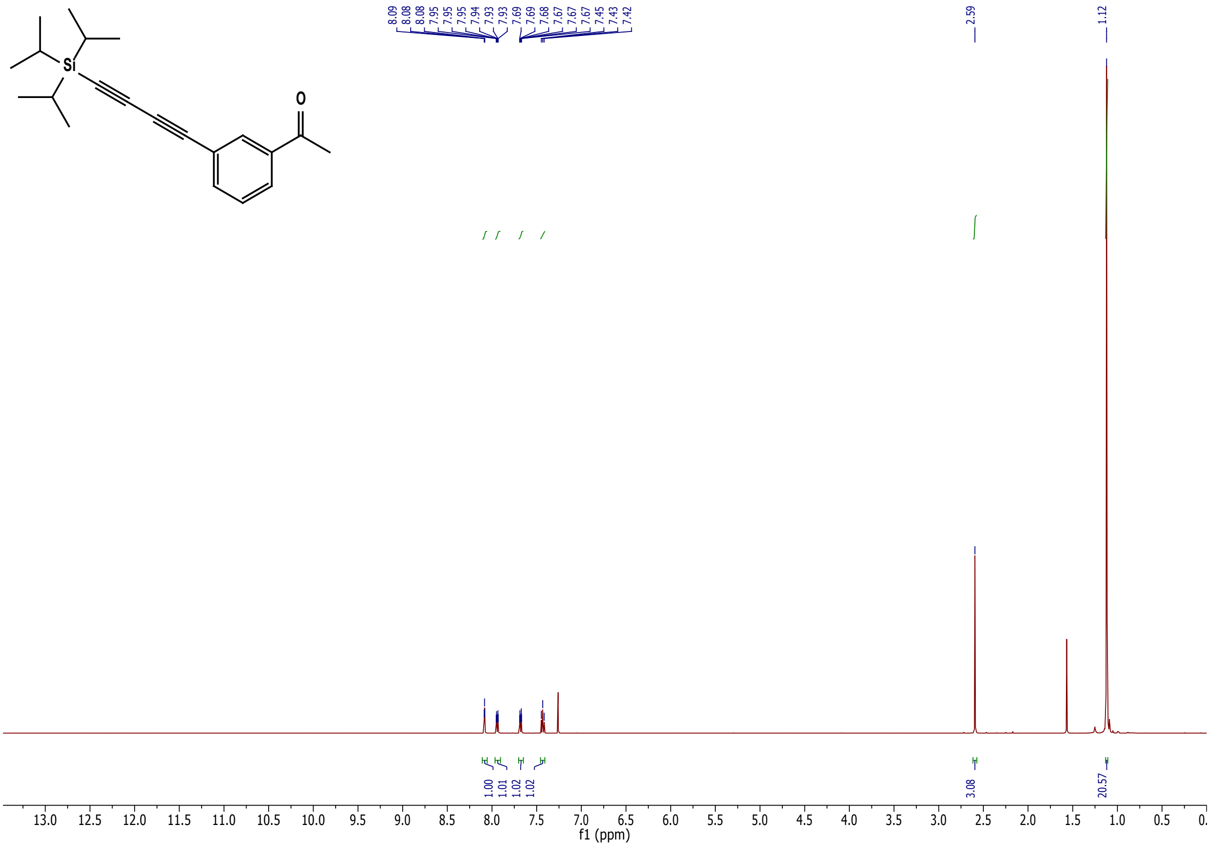

^1H NMR spectrum (500.1 MHz, $CDCl_3$) of 1-(3-((Triisopropylsilyl)buta-1,3-diyn-1-yl)phenyl)ethan-1-one (**38**)

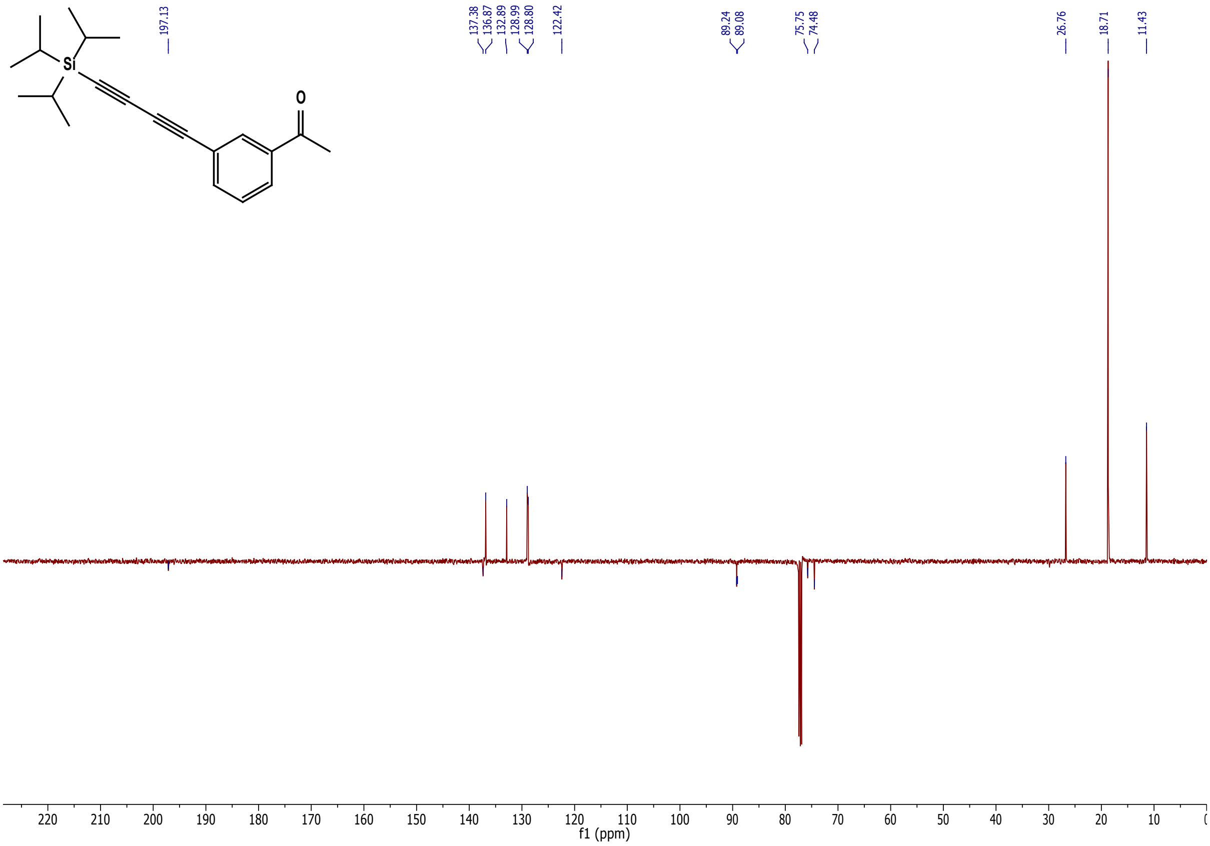

^{13}C NMR spectrum (126 MHz, $CDCl_3$) of 1-(3-((Triisopropylsilyl)buta-1,3-diyn-1-yl)phenyl)ethan-1-one (**38**)

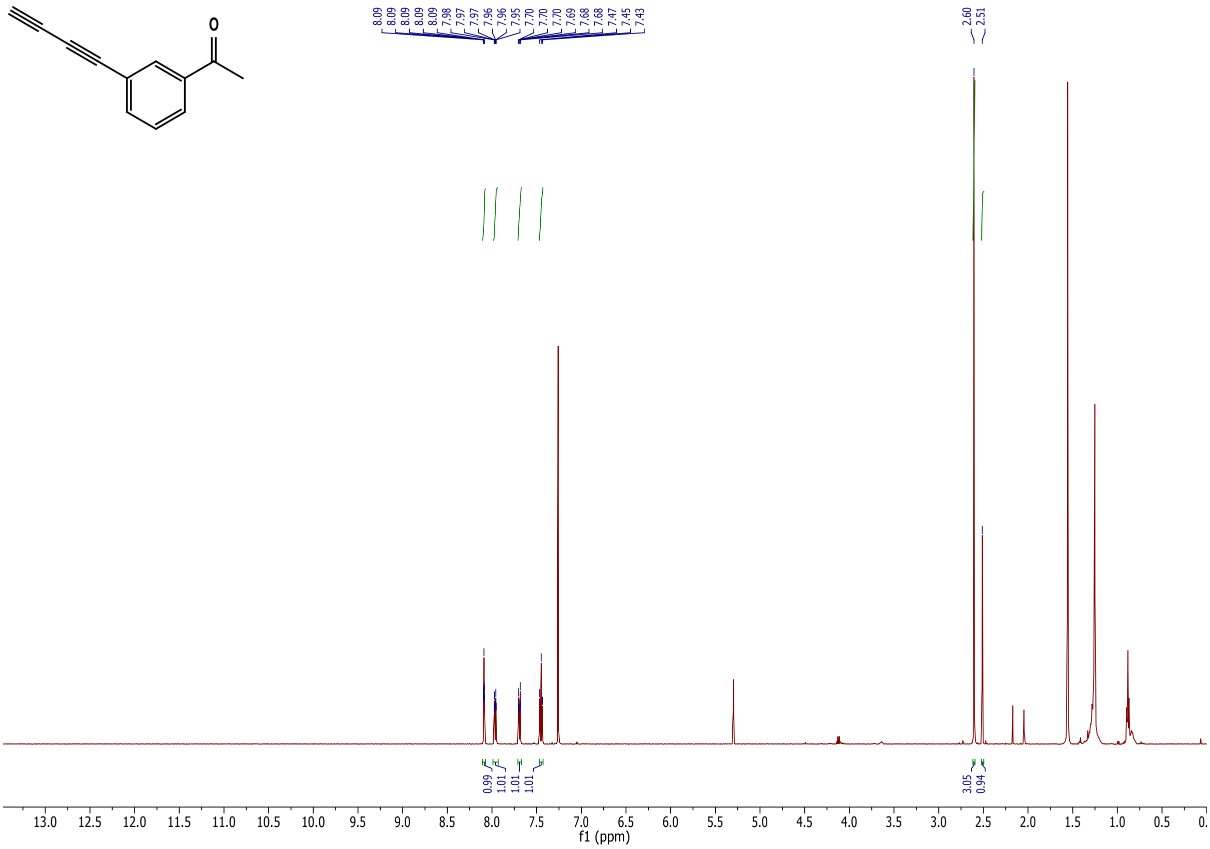

^{1}H NMR spectrum (500.1 MHz, $CDCl_3$) of 1-(3-(Buta-1,3-diyn-1-yl)phenyl)ethan-1-one (**39**)

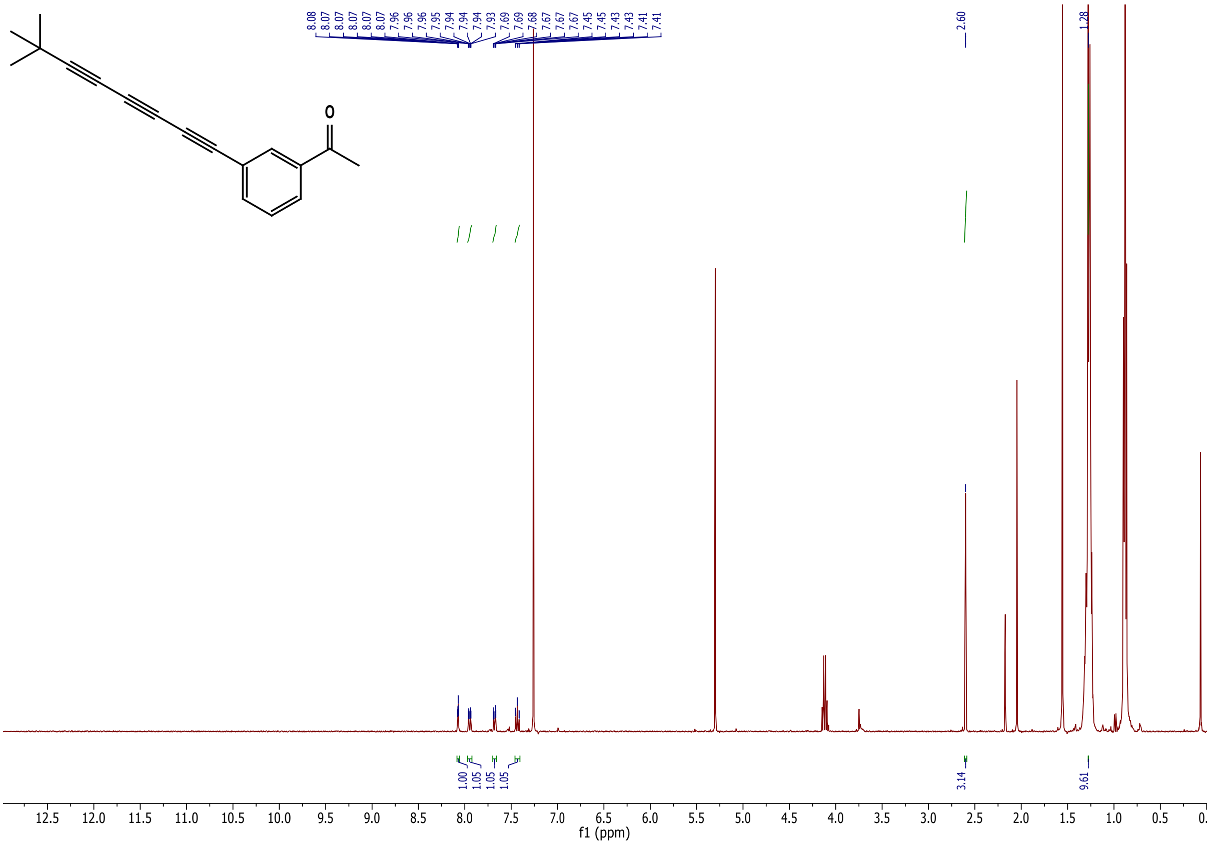

^{1}H NMR spectrum (500.1 MHz, $CDCl_3$) of 1-(3-(7,7-Dimethylocta-1,3,5-triyn-1-yl)phenyl)ethan-1-one (**19**)

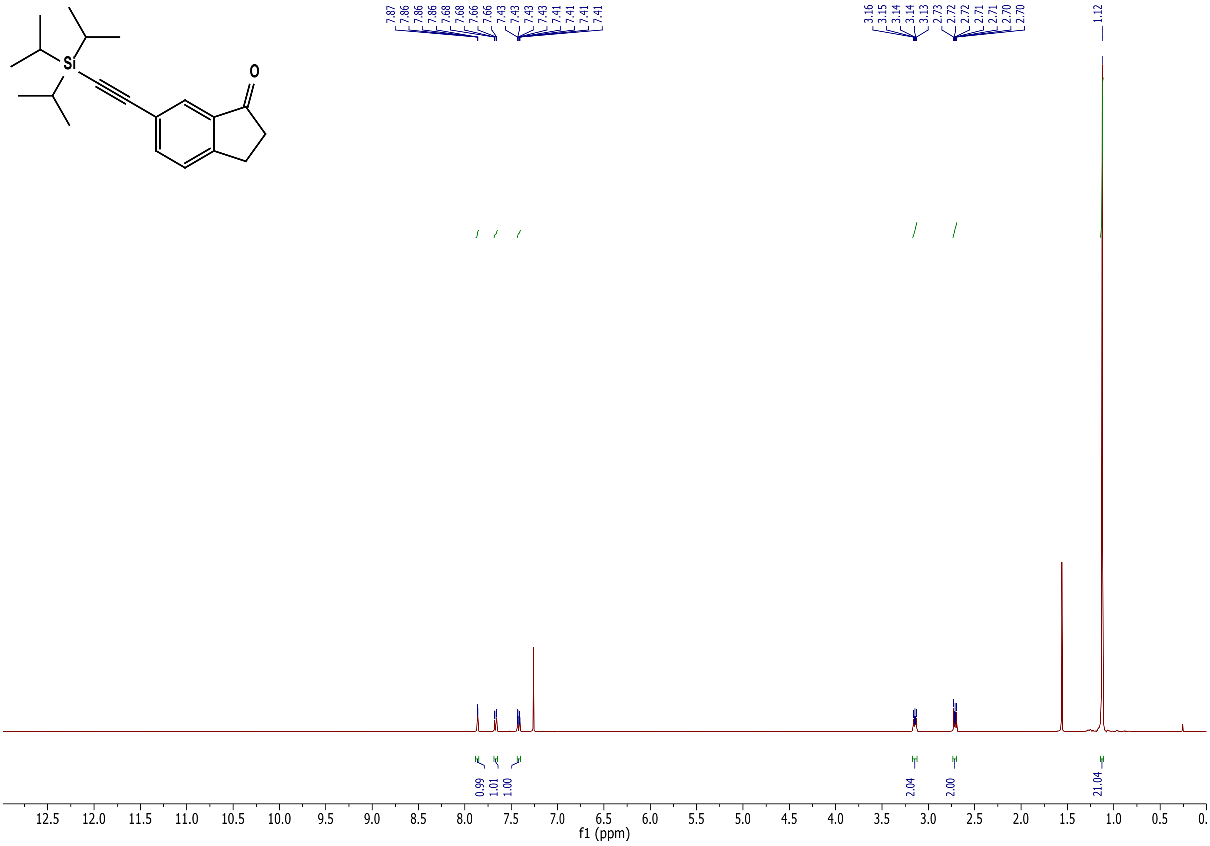

^{1}H NMR spectrum (400.1 MHz, $CDCl_3$) of 6-((Triisopropylsilyl)ethynyl)-2,3-dihydro-1*H*-inden-1-one (**43**)

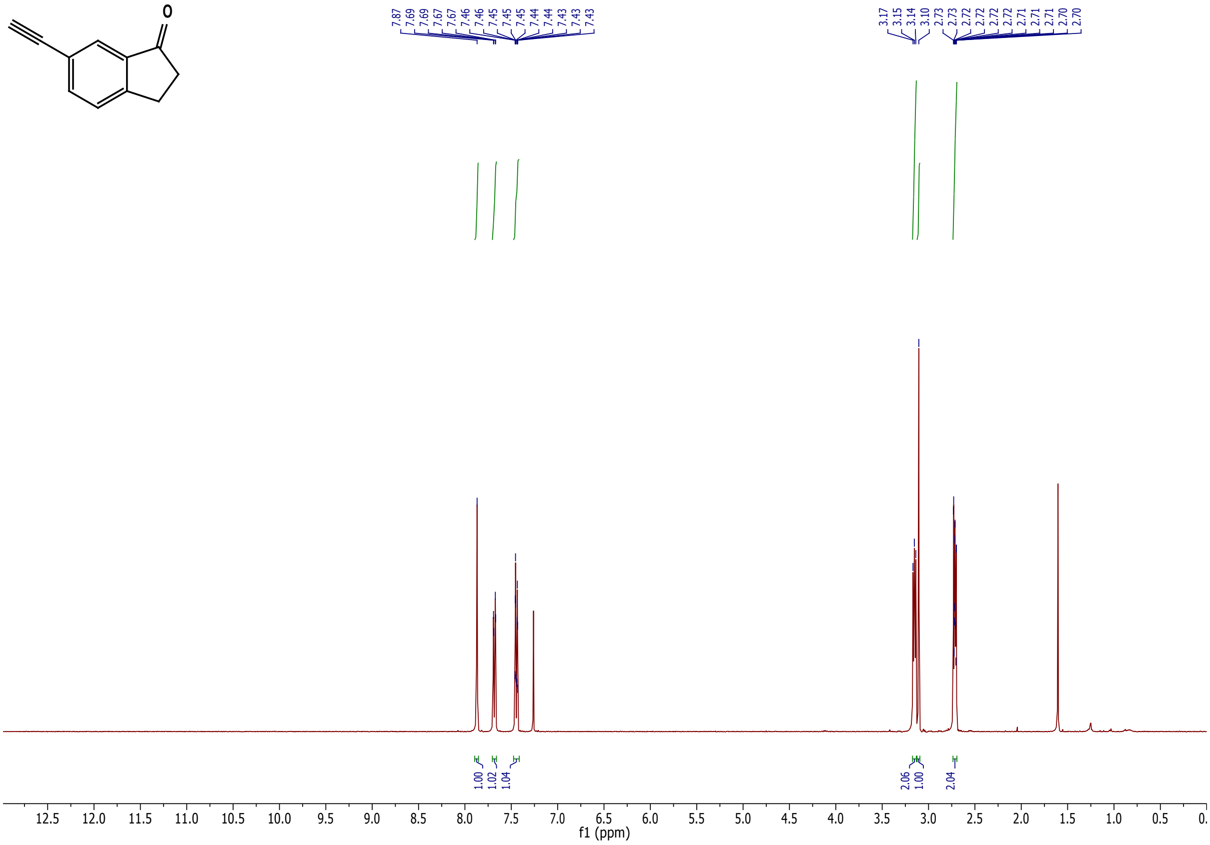

^{1}H NMR spectrum (400.1 MHz, $CDCl_3$) of 6-Ethynyl-2,3-dihydro-1*H*-inden-1-one (**45**)

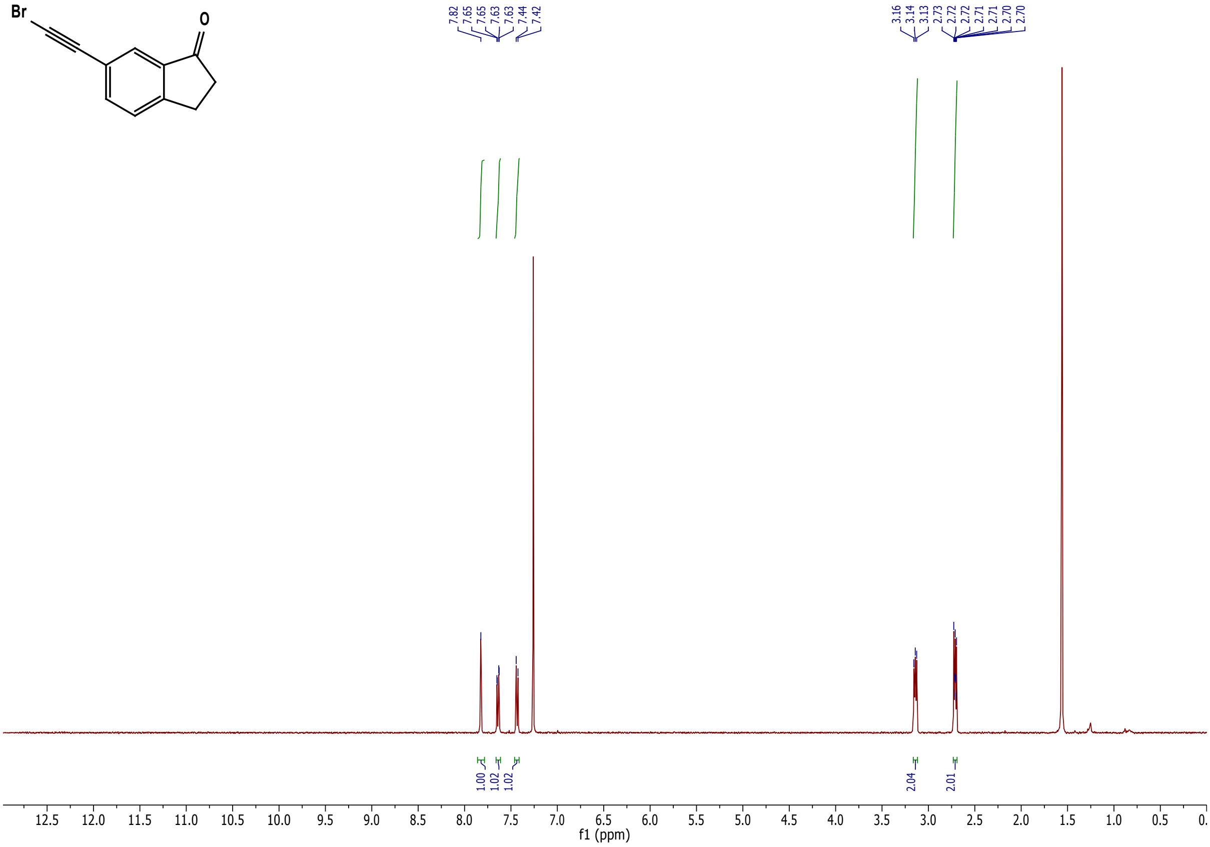

^{1}H NMR spectrum (400.1 MHz, $CDCl_3$) of 6-(Bromoethynyl)-2,3-dihydro-1*H*-inden-1-one (**47**)

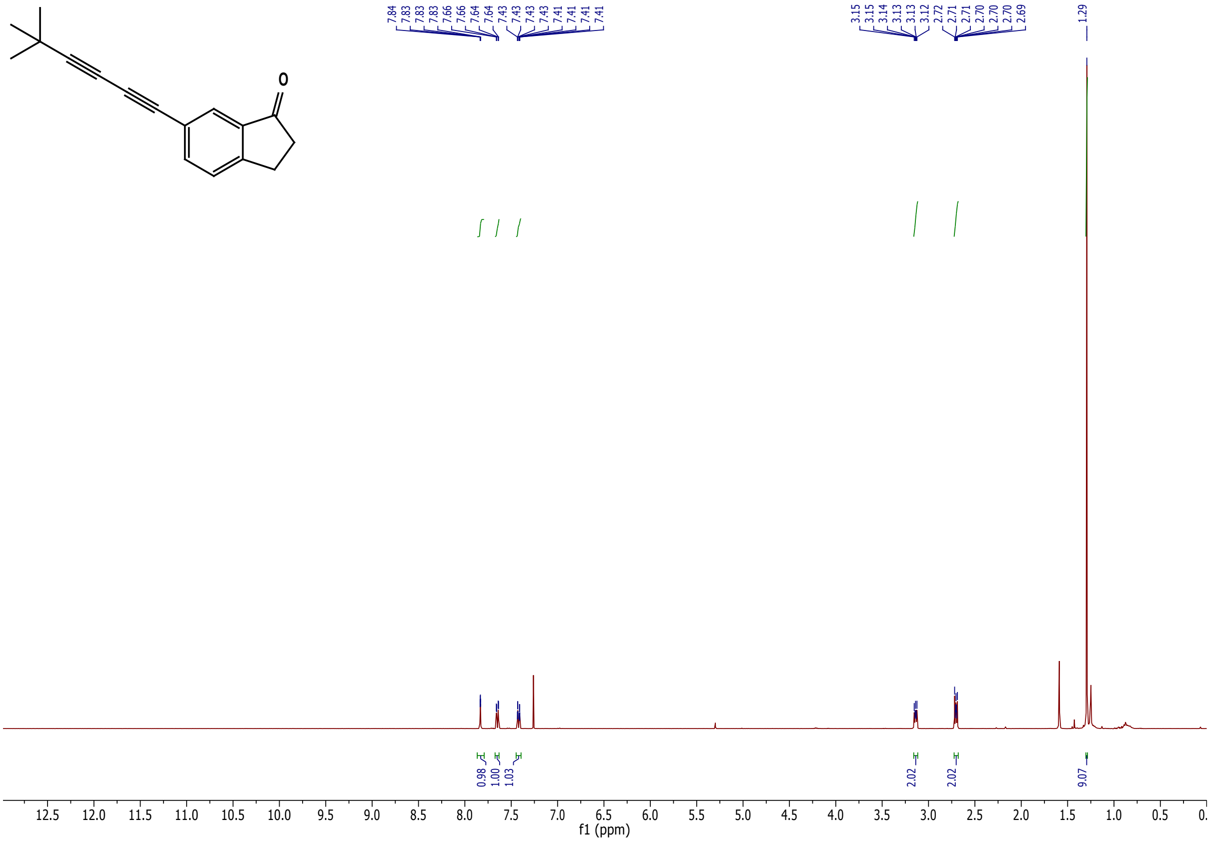

^{1}H NMR spectrum (400.1 MHz, $CDCl_3$) of 6-(5,5-Dimethylhexa-1,3-diyn-1-yl)-2,3-dihydro-1*H*-inden-1-one (**16**)

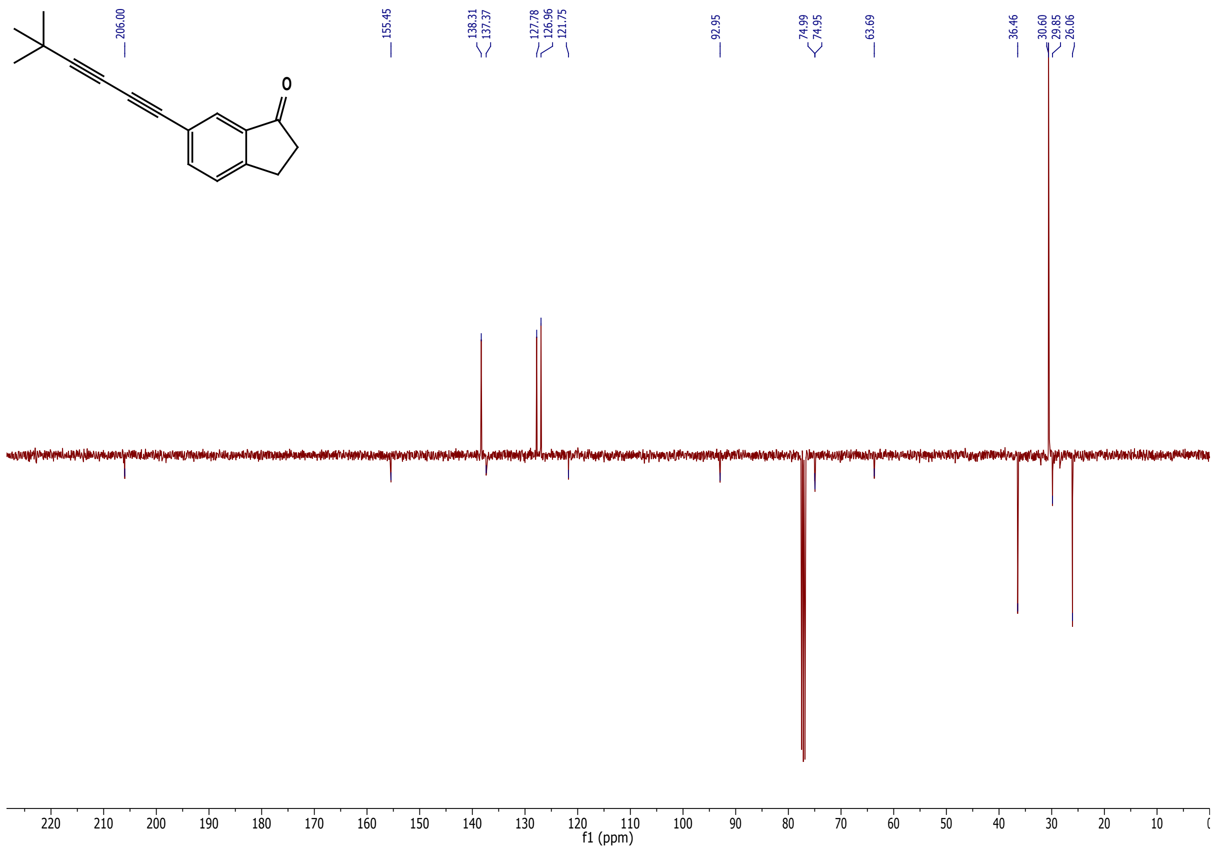

Figure S_. ^{13}C NMR spectrum (126 MHz, $CDCl_3$) of 6-(5,5-Dimethylhexa-1,3-diyn-1-yl)-2,3-dihydro-1*H*-inden-1-one (**16**)

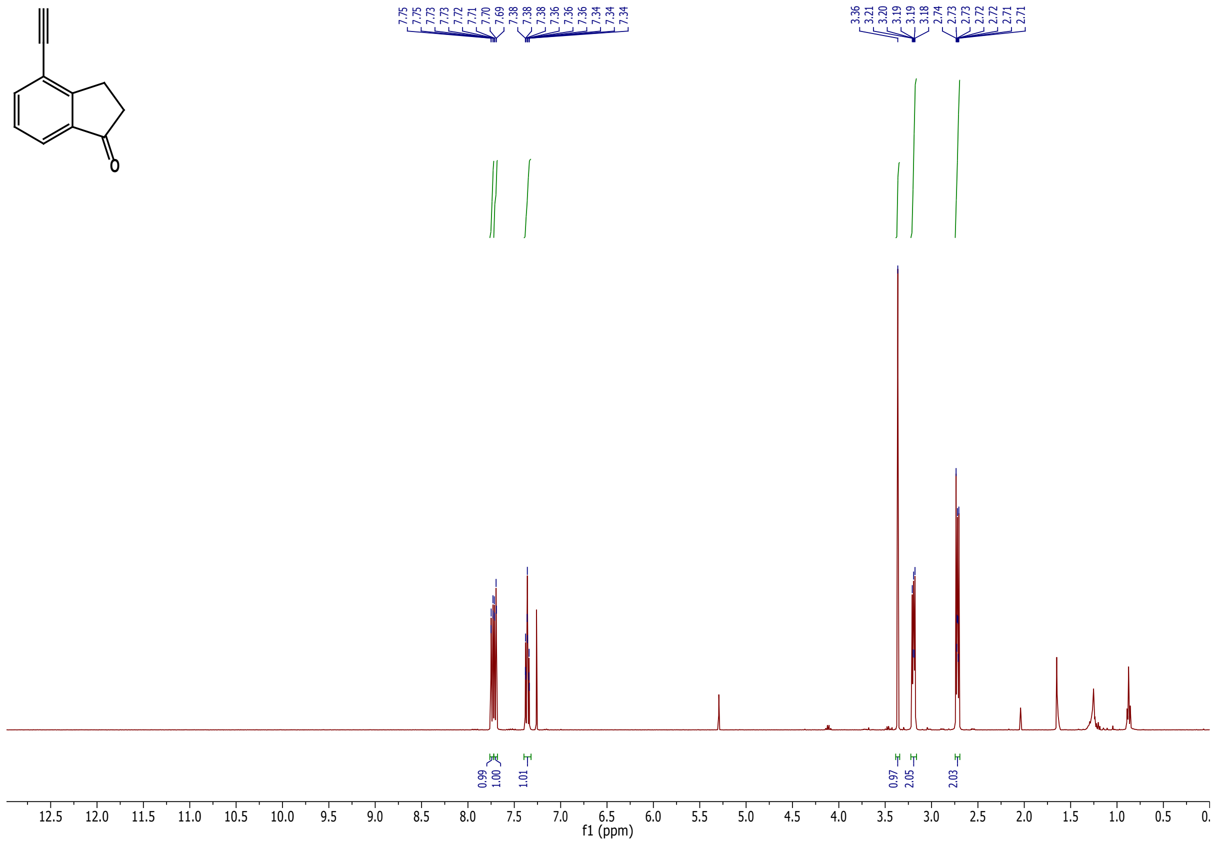

^{1}H NMR spectrum (400.1 MHz, $CDCl_3$) of 4-Ethynyl-2,3-dihydro-1*H*-inden-1-one (**46**)

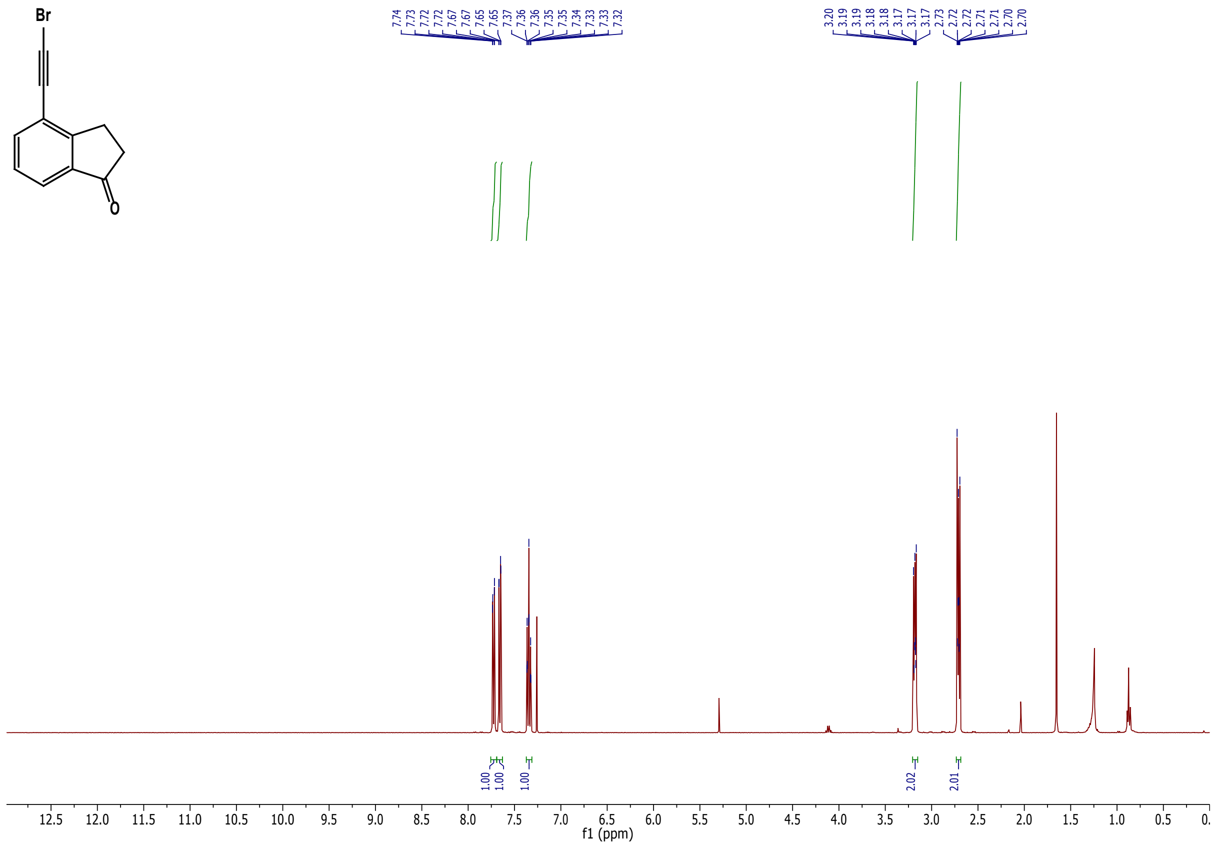

^{1}H NMR spectrum (400.1 MHz, $CDCl_3$) of 4-(Bromoethynyl)-2,3-dihydro-1*H*-inden-1-one (**48**)

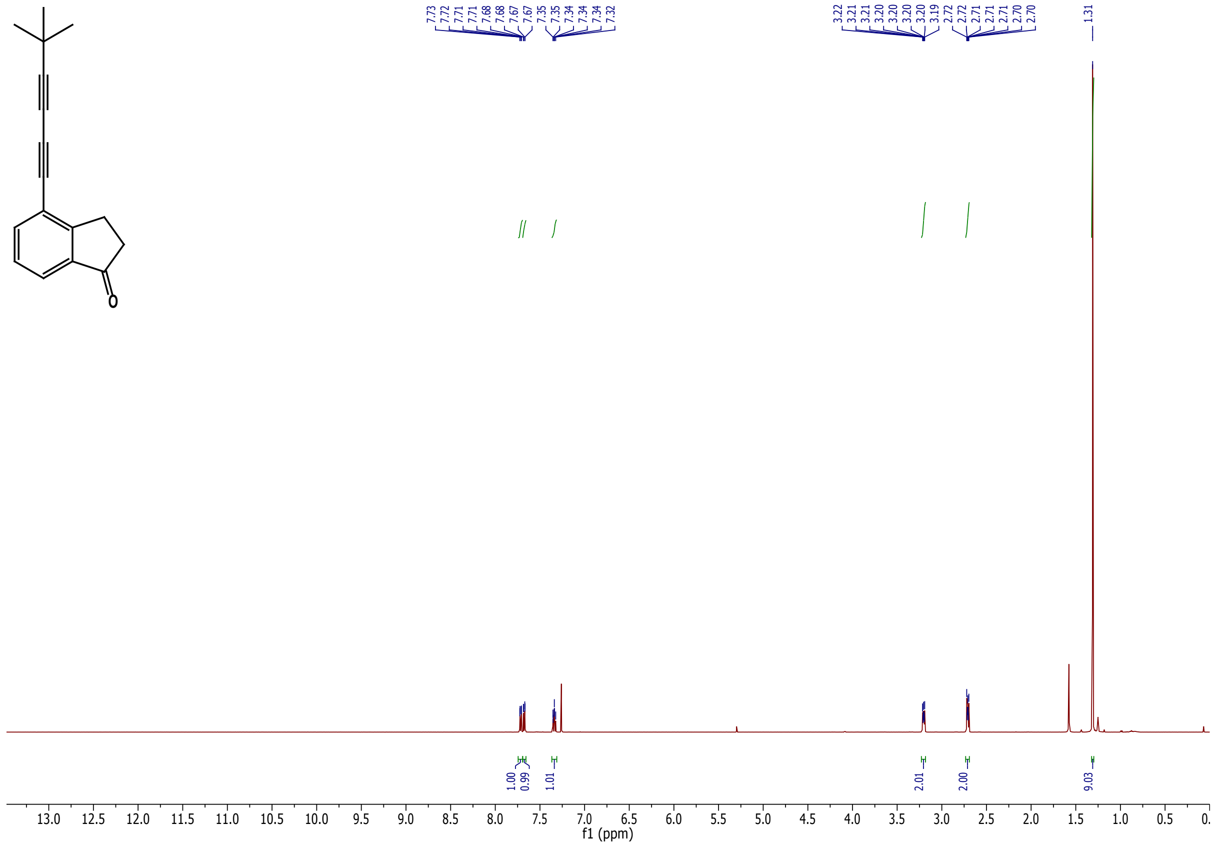

^{1}H NMR spectrum (500.1 MHz, $CDCl_3$) of 4-(5,5-Dimethylhexa-1,3-diyn-1-yl)-2,3-dihydro-1*H*-inden-1-one (**17**)

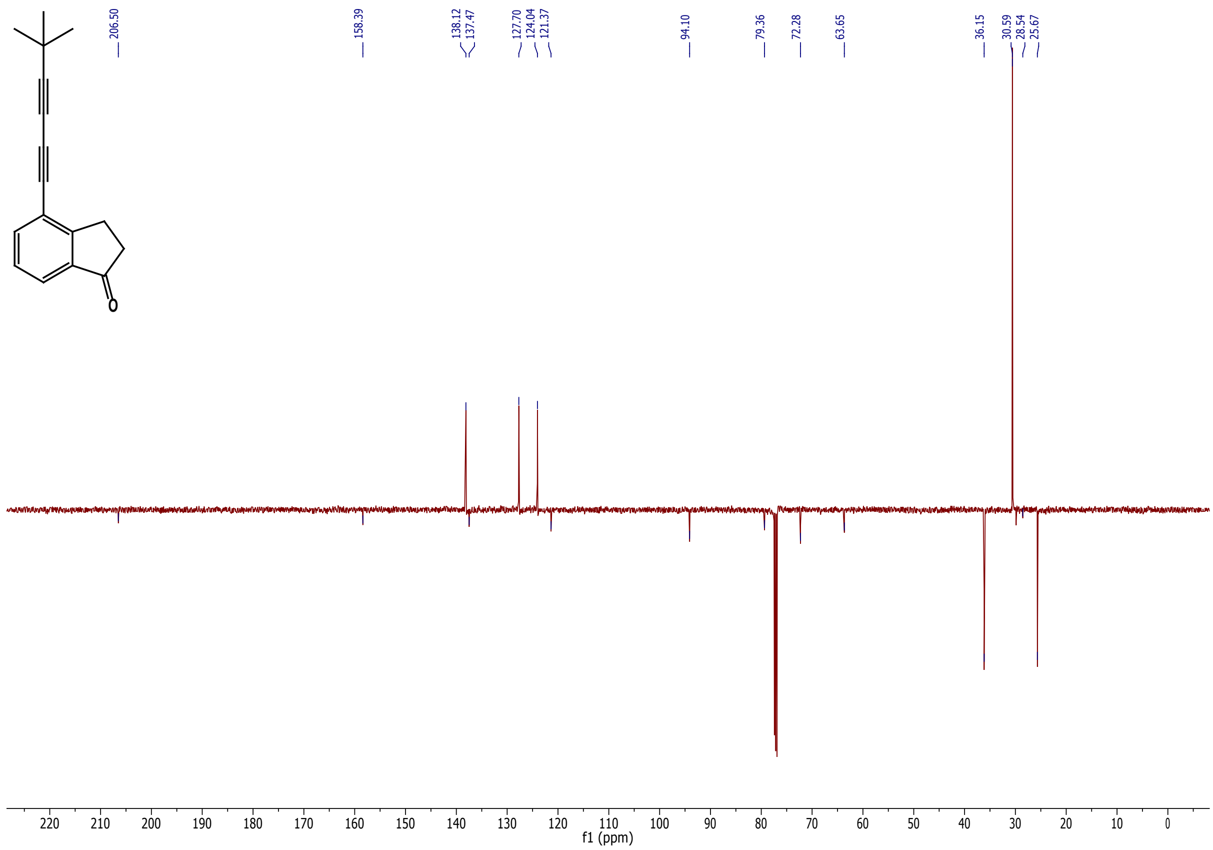

^{13}C NMR spectrum (126 MHz, $CDCl_3$) of 4-(5,5-Dimethylhexa-1,3-diyn-1-yl)-2,3-dihydro-1*H*-inden-1-one (**17**)

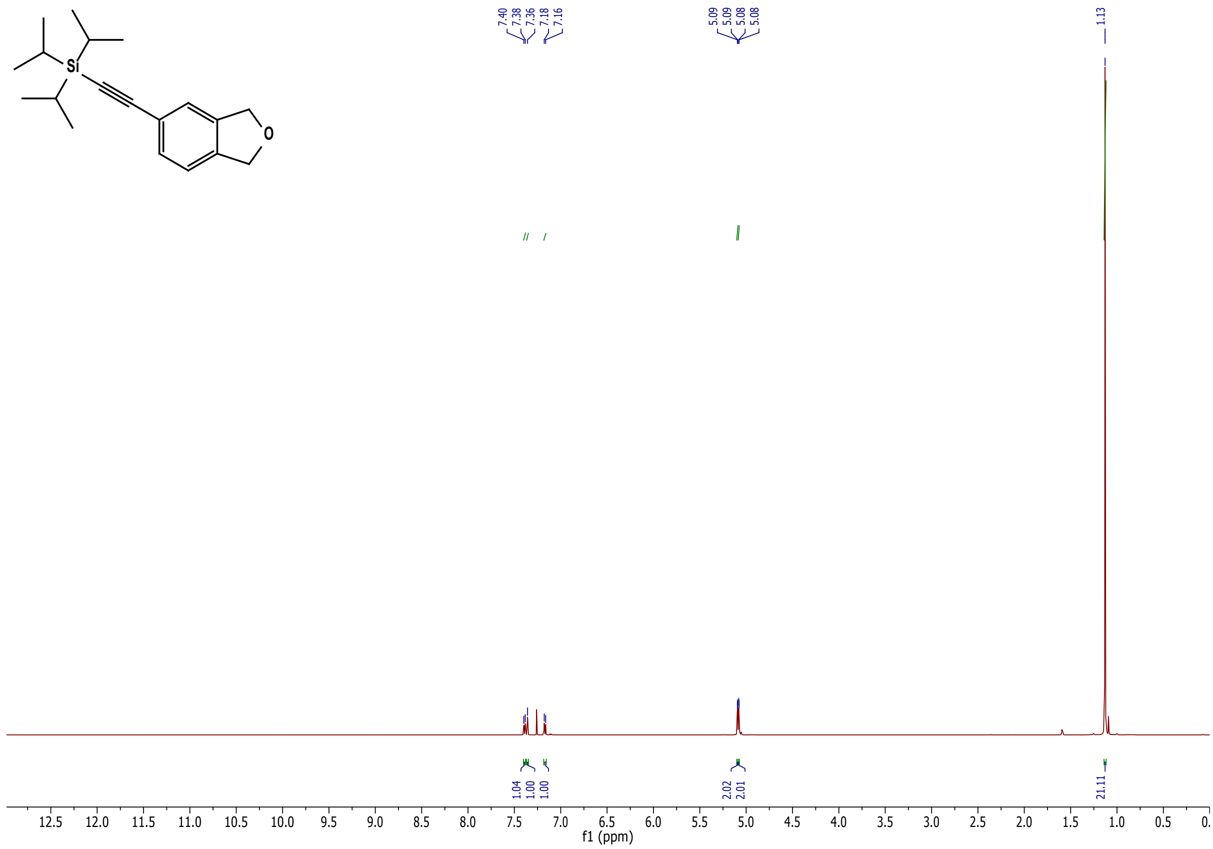

^{1}H NMR spectrum (500.1 MHz, $CDCl_3$) of ((1,3-Dihydroisobenzofuran-5-yl)ethynyl)triisopropylsilane (**53**)

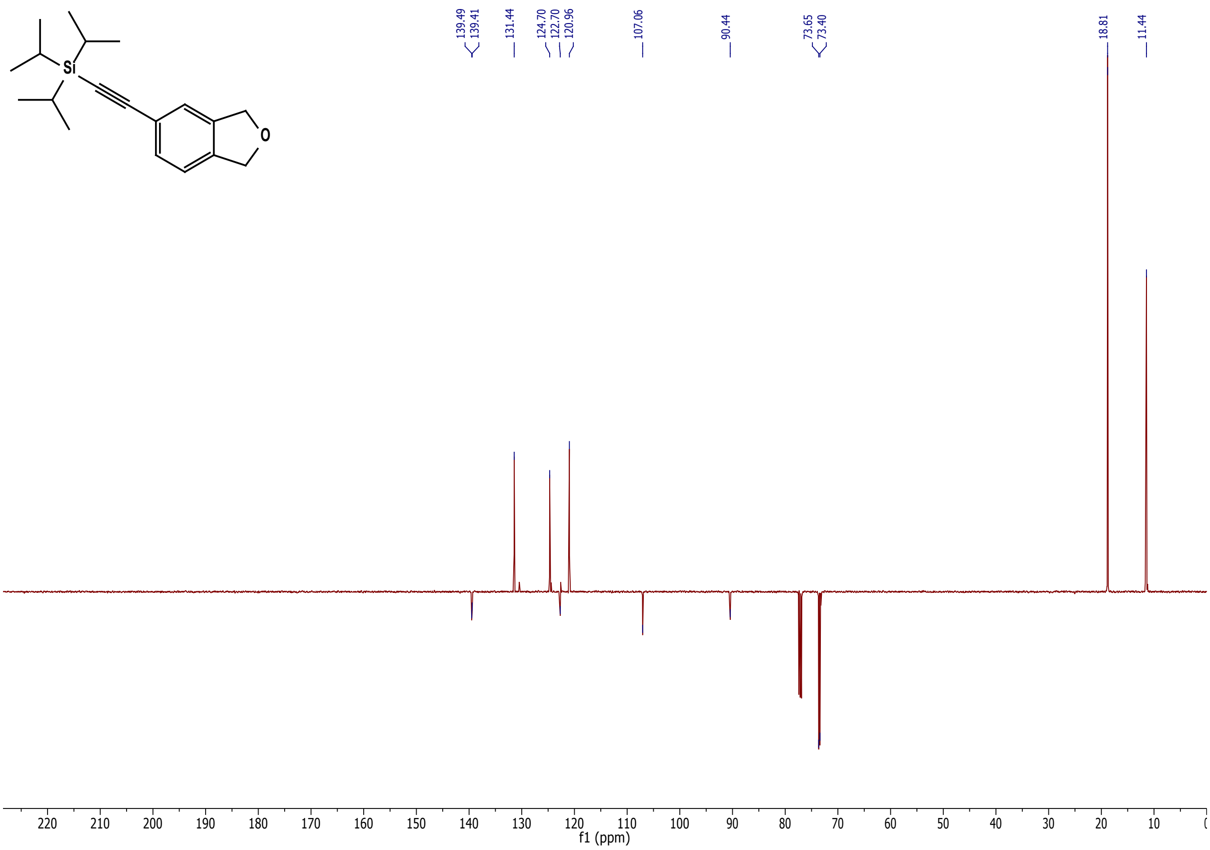

^{13}C NMR spectrum (126 MHz, $CDCl_3$) of ((1,3-Dihydroisobenzofuran-5-yl)ethynyl)triisopropylsilane (**53**)

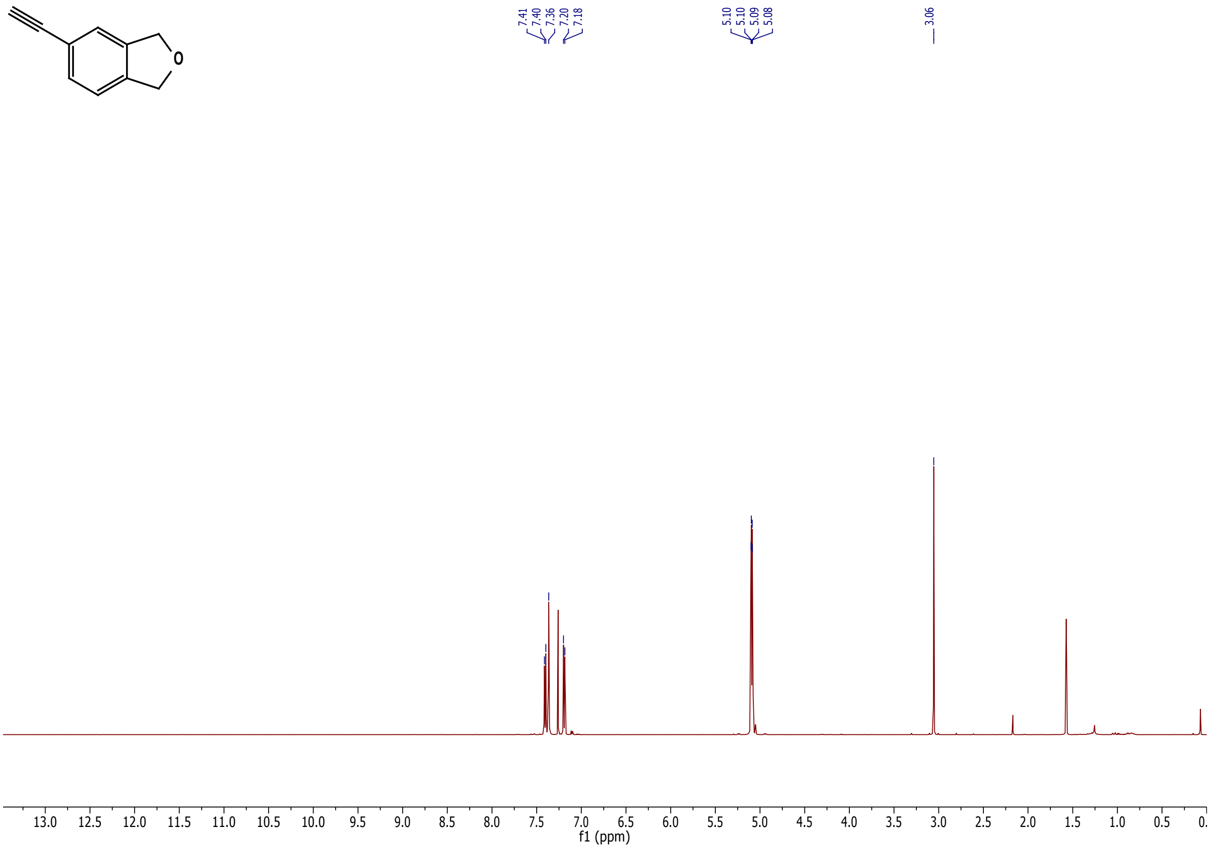

^{1}H NMR spectrum (500.1 MHz, $CDCl_3$) of 5-Ethynyl-1,3-dihydroisobenzofuran (**54**)

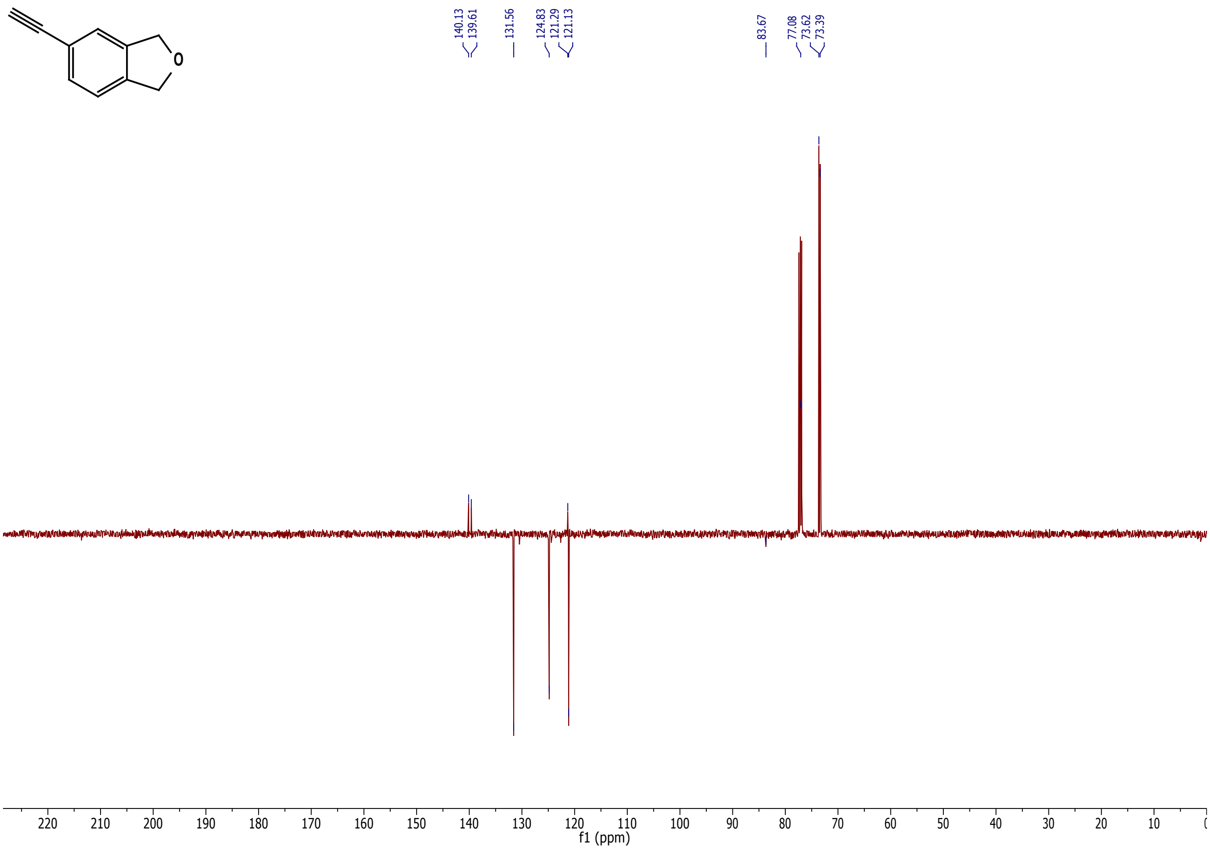

^{13}C NMR spectrum (126 MHz, $CDCl_3$) of 5-Ethynyl-1,3-dihydroisobenzofuran (**54**)

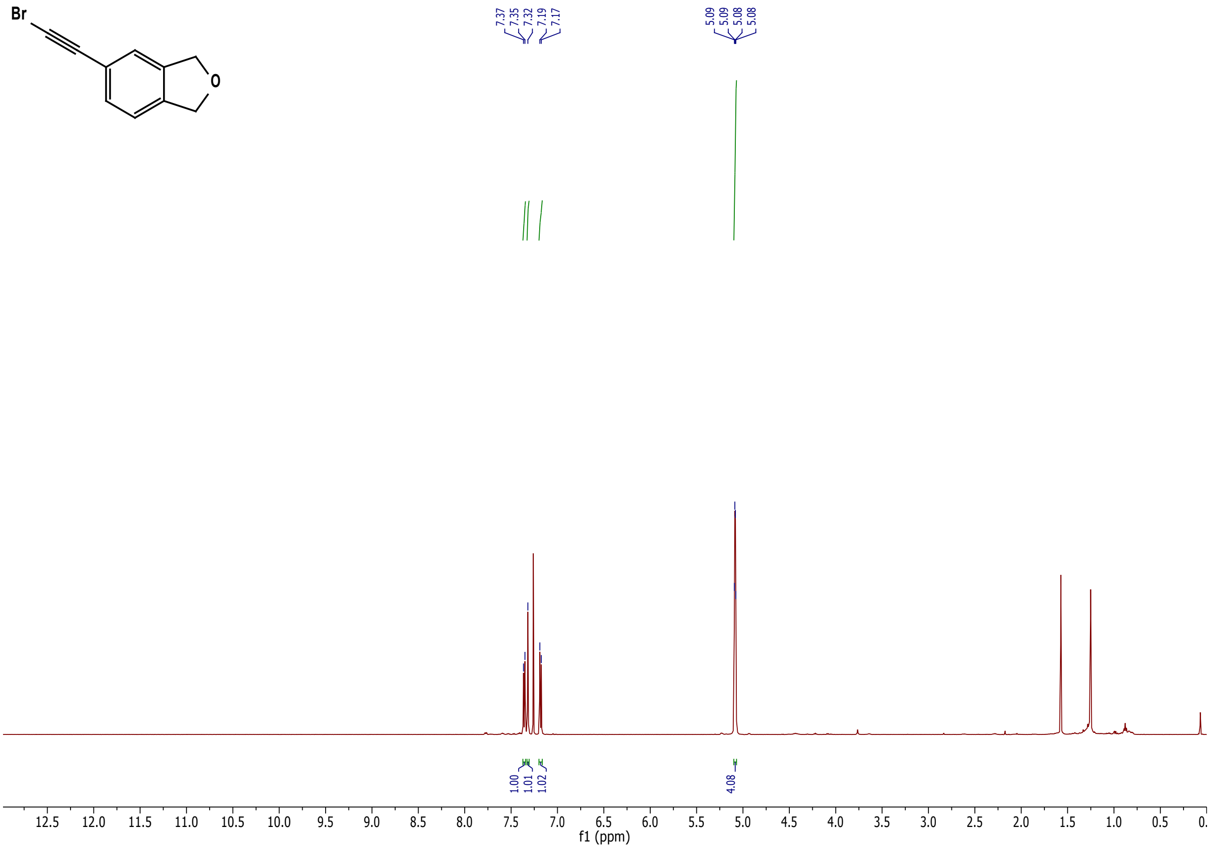

^{1}H NMR spectrum (500.1 MHz, $CDCl_3$) of 5-(Bromoethynyl)-1,3-dihydroisobenzofuran (**55**)

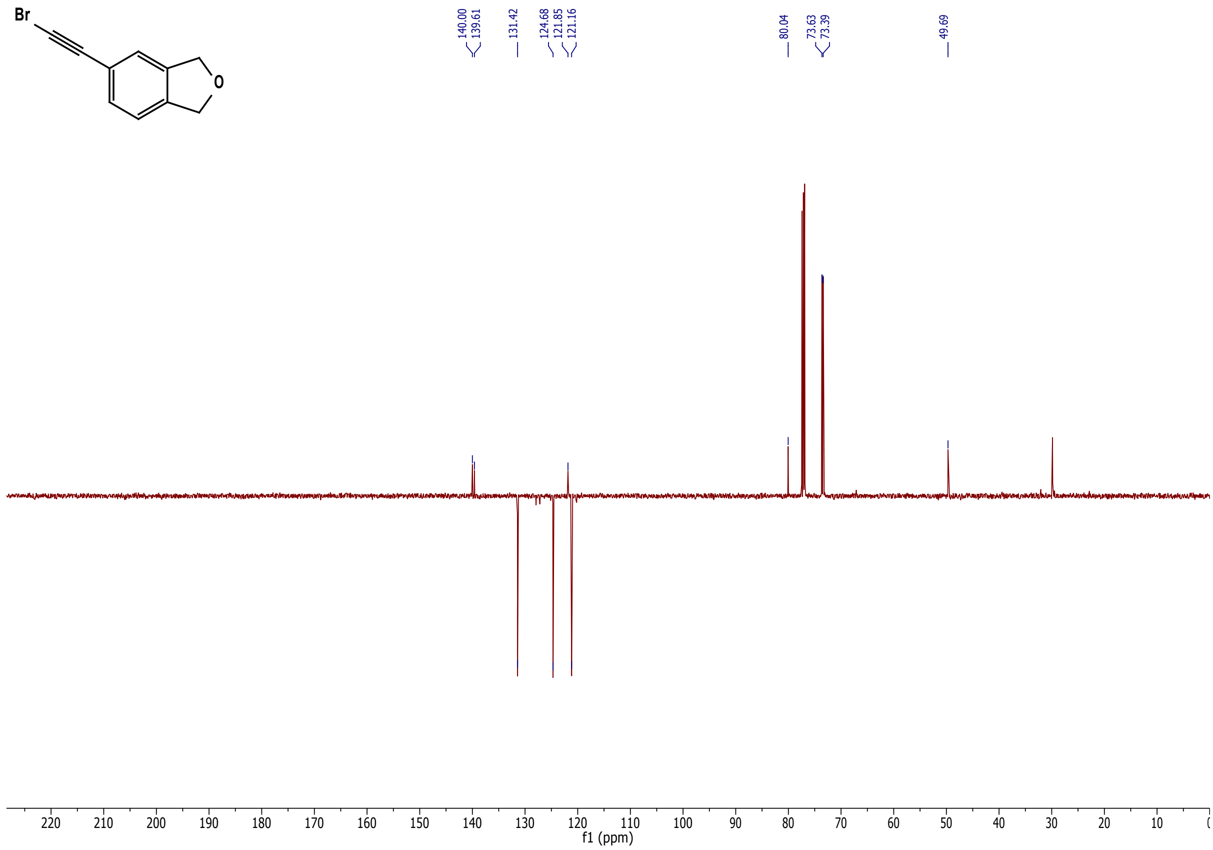

^{13}C NMR spectrum (126 MHz, $CDCl_3$) of 5-(Bromoethynyl)-1,3-dihydroisobenzofuran (**55**)

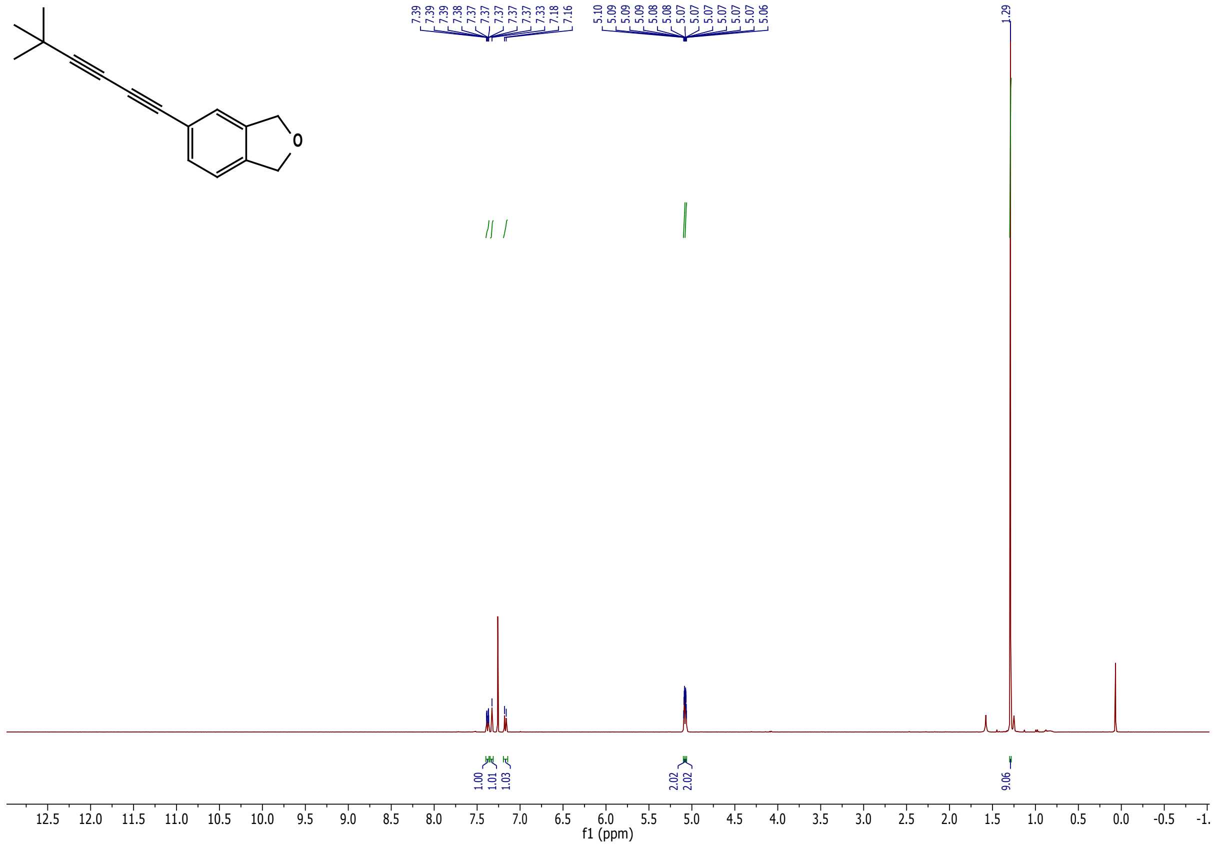

^{1}H NMR spectrum (400.1 MHz, $CDCl_3$) of 5-(5,5-Dimethylhexa-1,3-diyn-1-yl)-1,3-dihydroisobenzofuran (**18**)

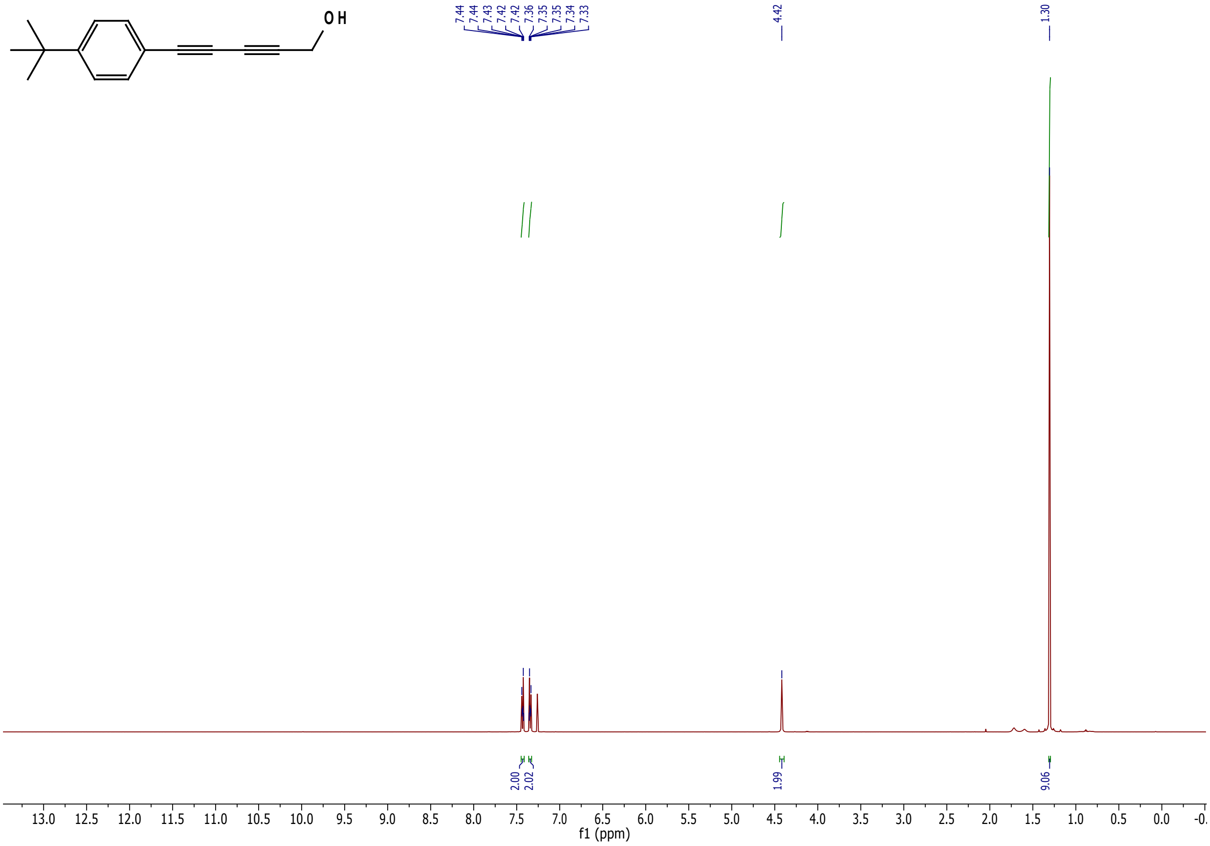

^1H NMR spectrum (500.1 MHz, $CDCl_3$) of 5-(4-(*Tert*-butyl)phenyl)penta-2,4-diyn-1-ol (**20**)

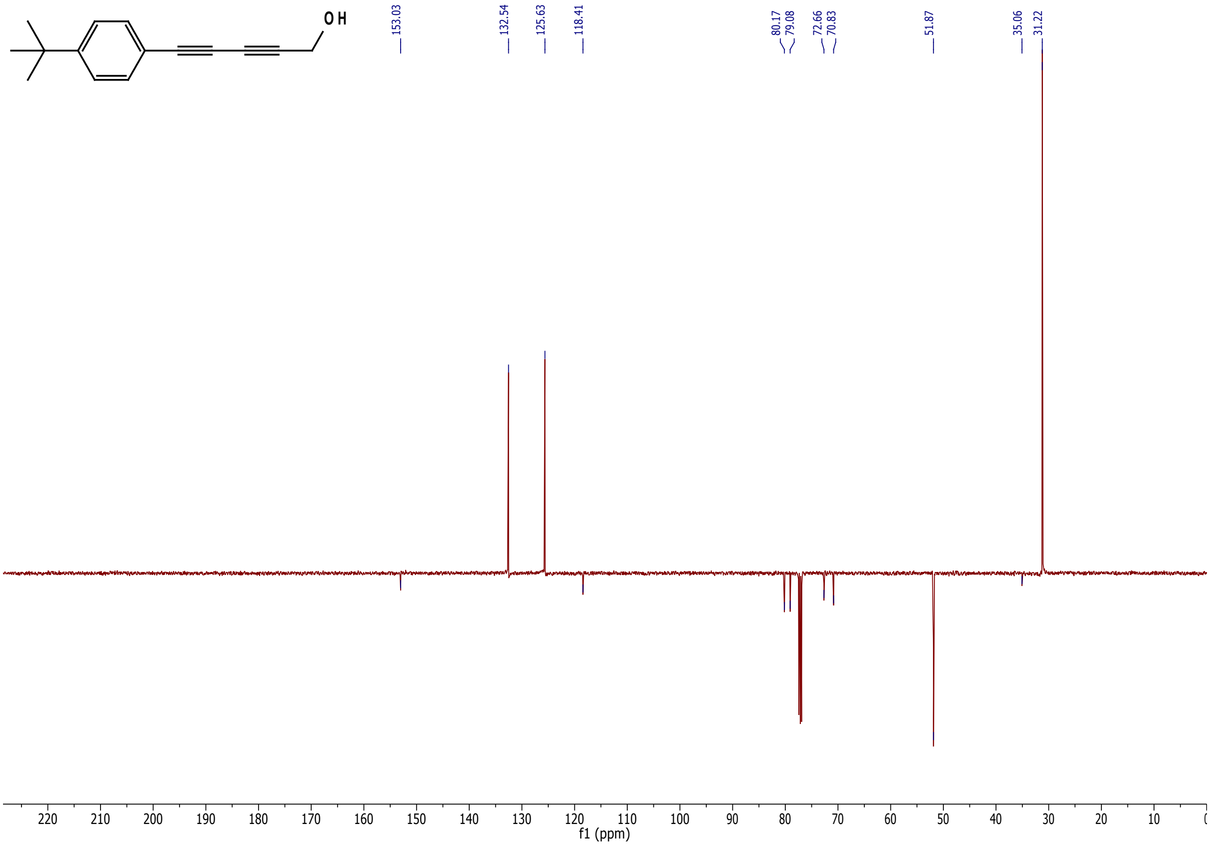

^{13}C NMR spectrum (126 MHz, $CDCl_3$) of 5-(4-(*Tert*-butyl)phenyl)penta-2,4-diyn-1-ol (**20**)

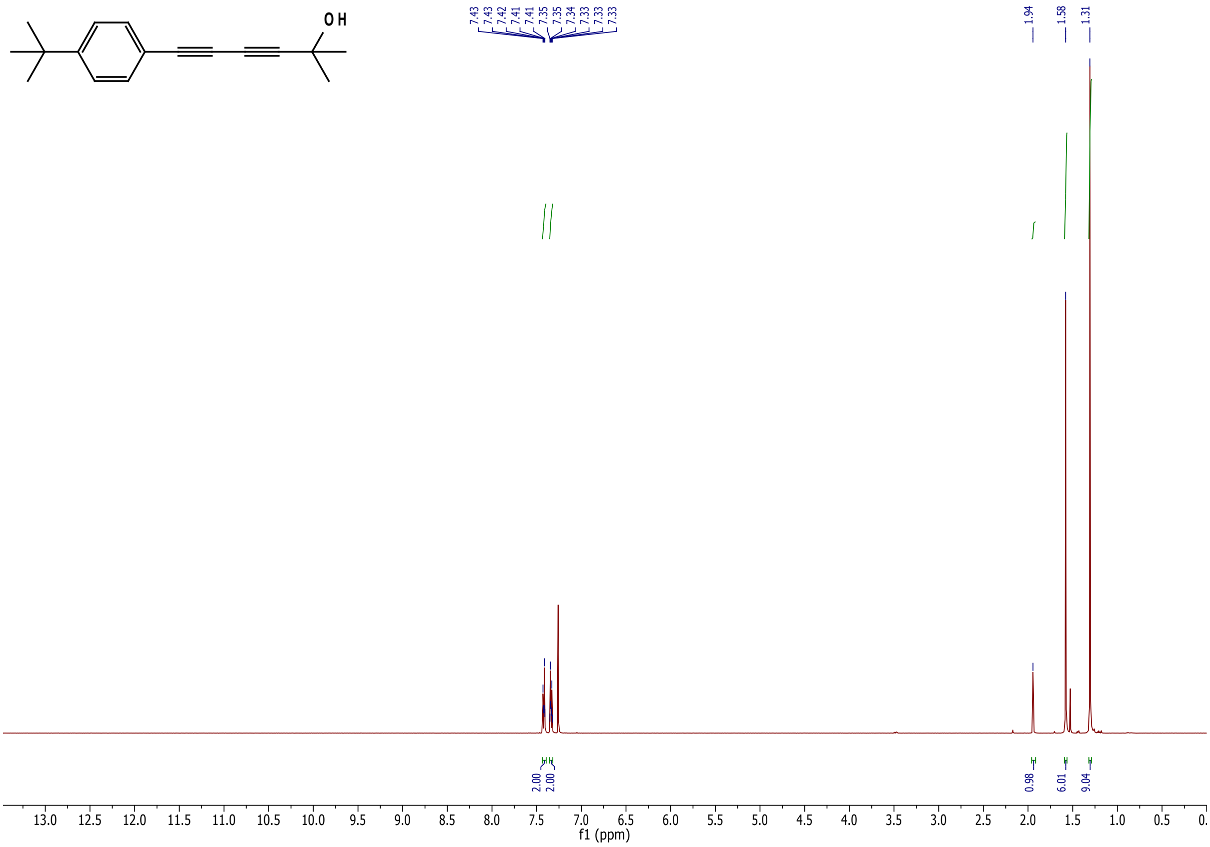

^{1}H NMR spectrum (500.1 MHz, $CDCl_3$) of 6-(4-(*Tert*-butyl)phenyl)-2-methylhexa-3,5-diyn-2-ol (**22**)

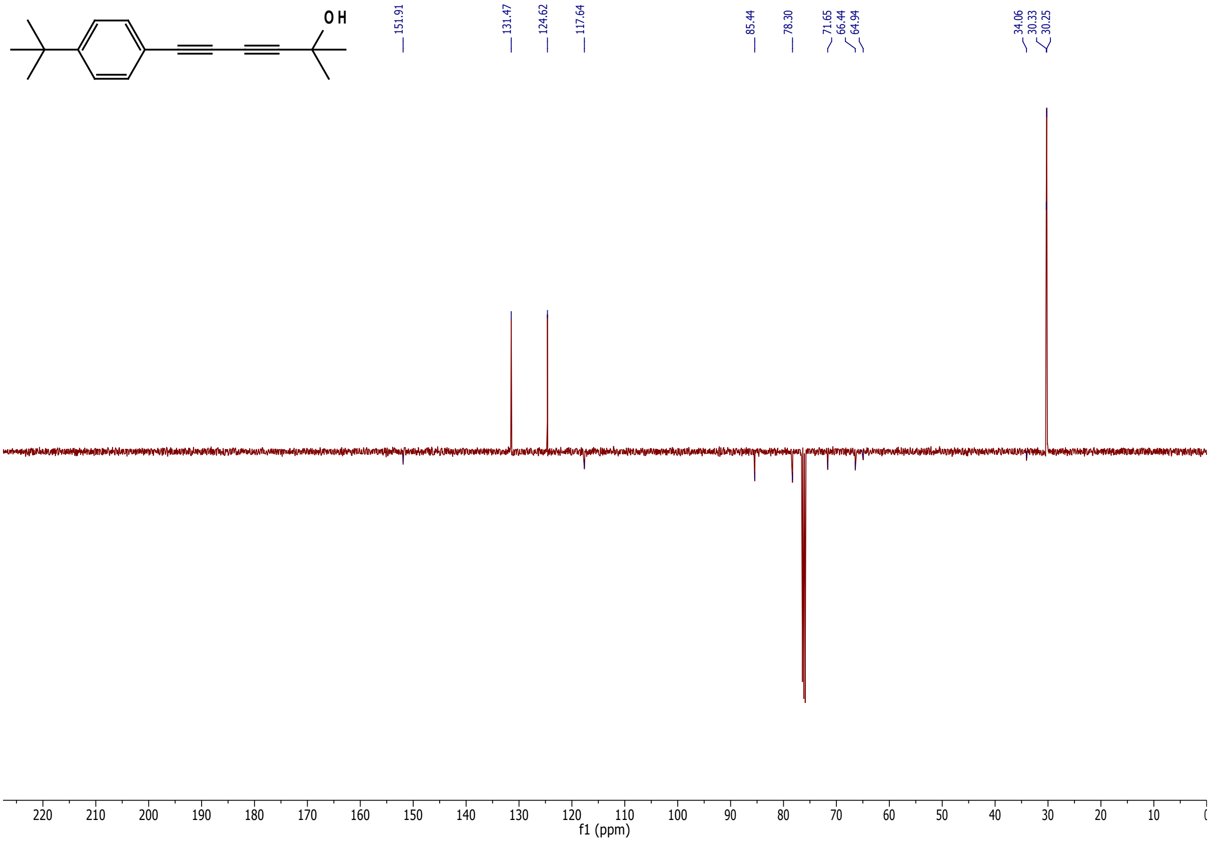

^{13}C NMR spectrum (126 MHz, $CDCl_3$) of 6-(4-(*Tert*-butyl)phenyl)-2-methylhexa-3,5-diyn-2-ol (**22**)

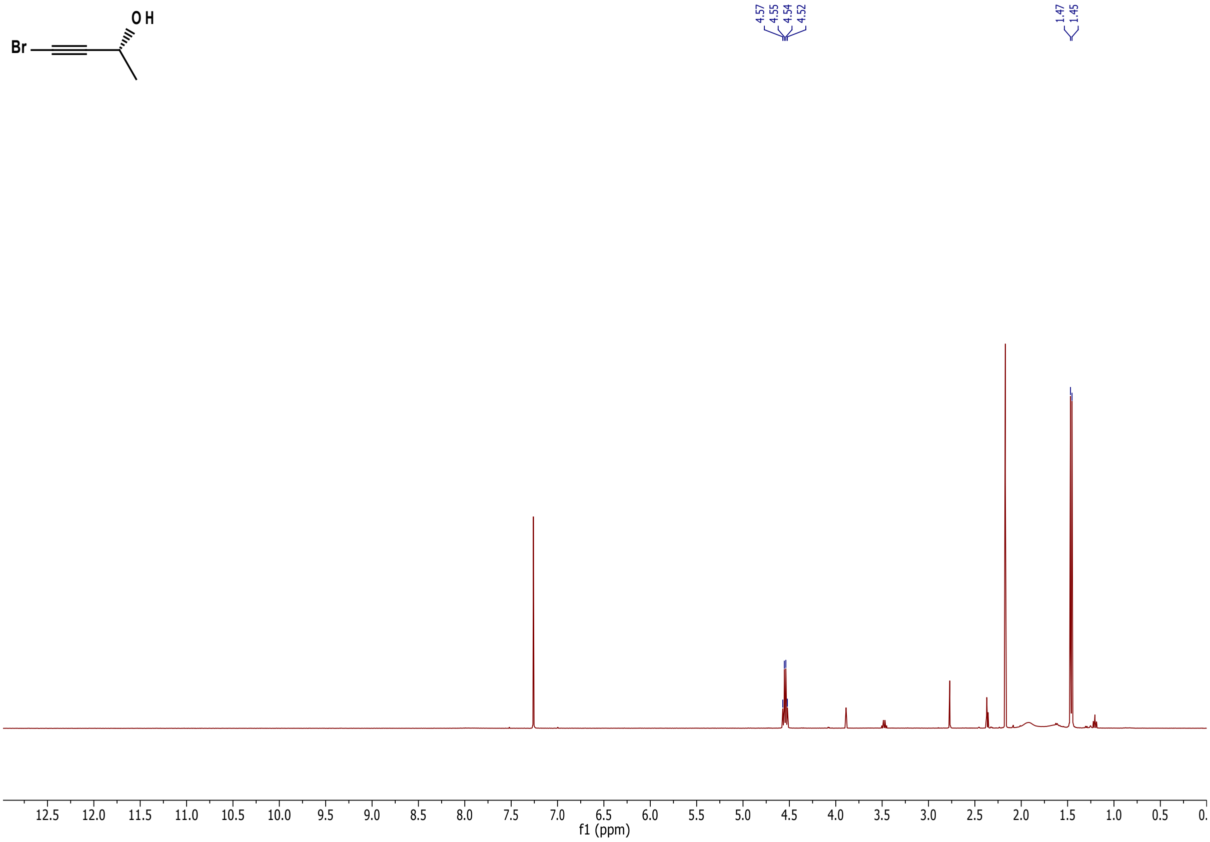

^{1}H NMR spectrum (500.1 MHz, $CDCl_3$) of (*R*)-4-Bromobut-3-yn-2-ol ((*R*)-**36**)

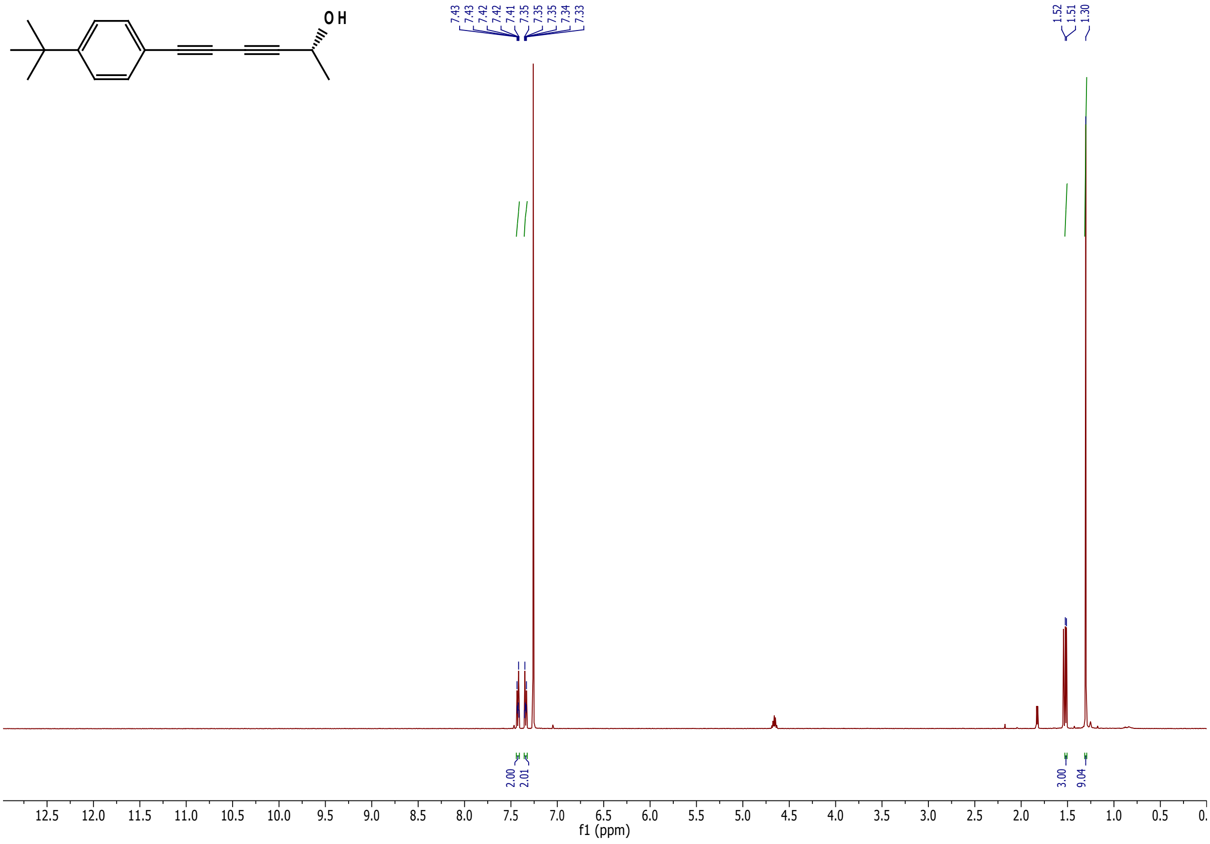

^{1}H NMR spectrum (500.1 MHz, $CDCl_3$) of (*R*)-6-(4-(*Tert*-butyl)phenyl)hexa-3,5-diyn-2-ol ((*R*)-**21**)

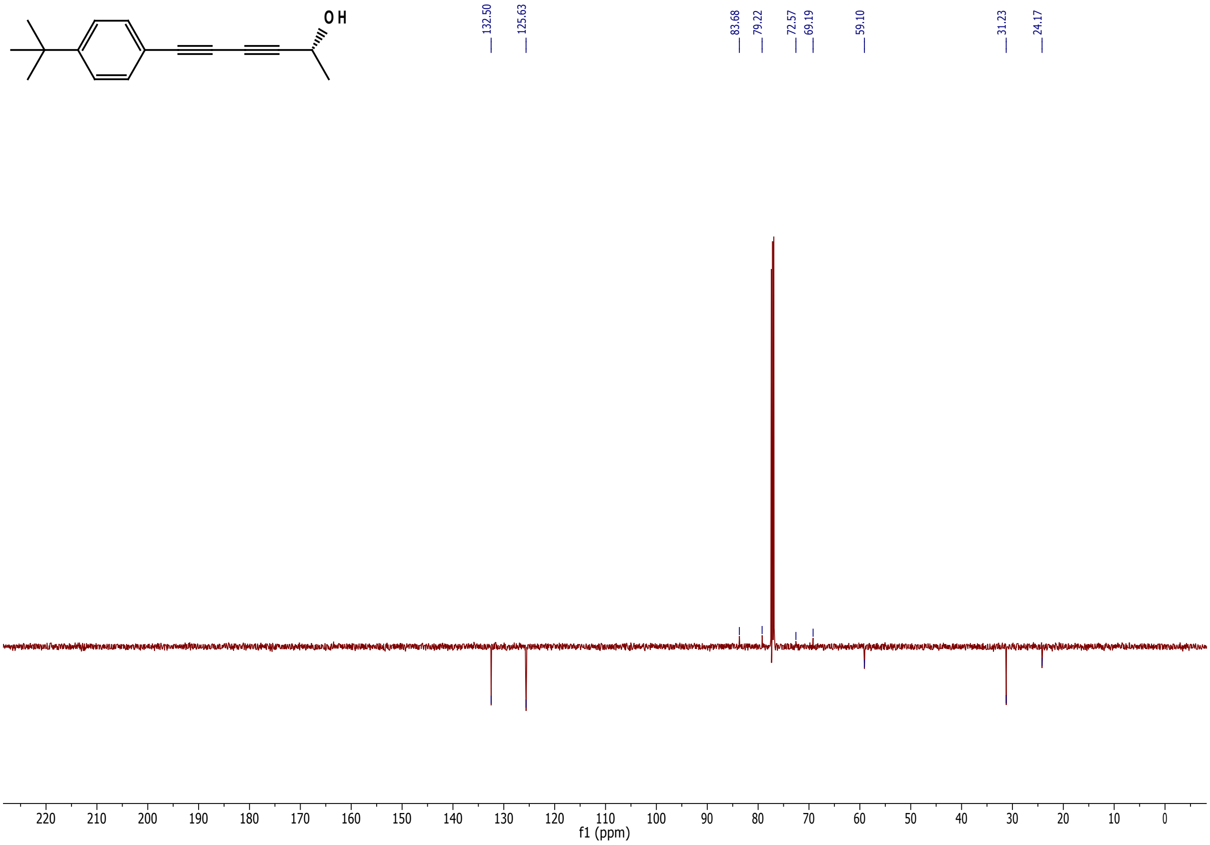

^{13}C NMR spectrum (126 MHz, $CDCl_3$) of (*R*)-6-(4-(*Tert*-butyl)phenyl)hexa-3,5-diyn-2-ol ((*R*)-**21**)

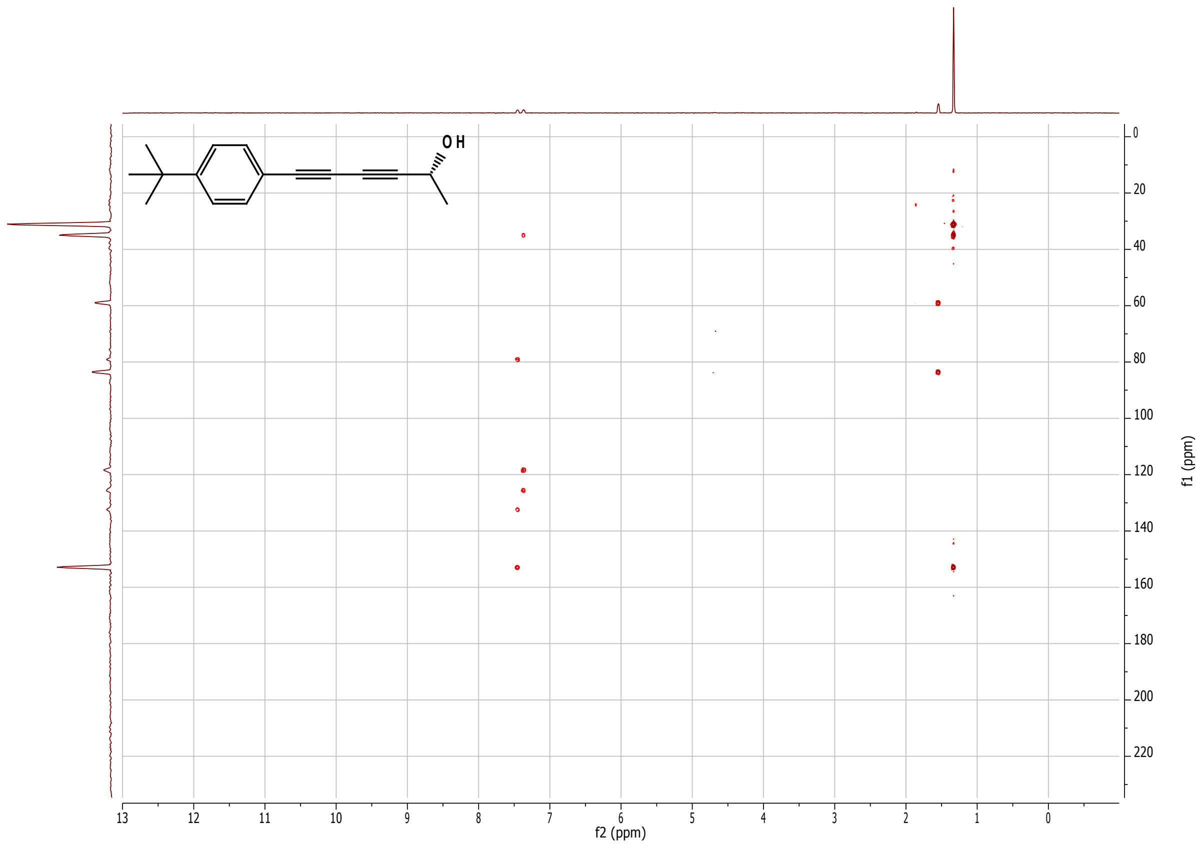

2D ^{1}H,^{13}C HMBC NMR spectrum (126 MHz, $CDCl_3$) of (*R*)-6-(4-(*Tert*-butyl)phenyl)hexa-3,5-diyn-2-ol ((*R*)-**21**)

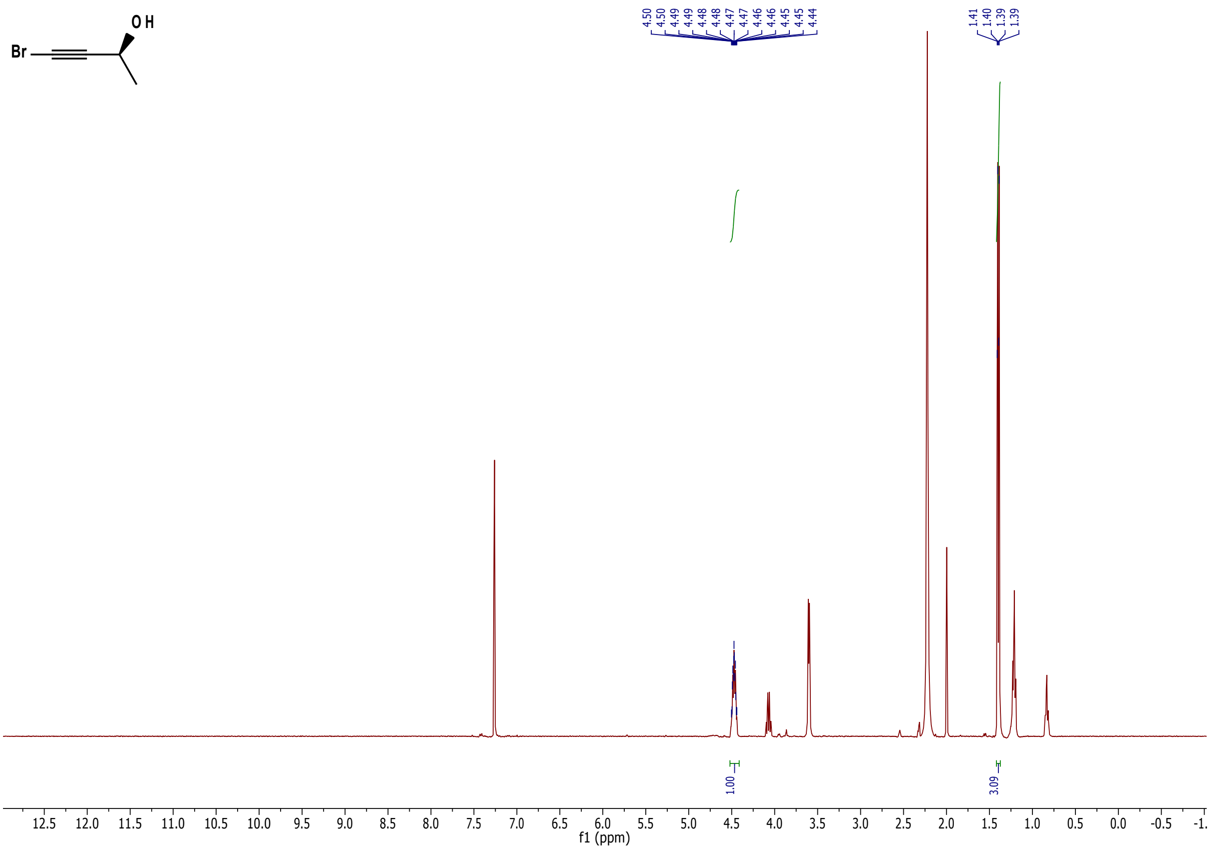

^{1}H NMR spectrum (500.1 MHz, $CDCl_3$) of (*S*)-4-Bromobut-3-yn-2-ol ((*S*)-**36**)

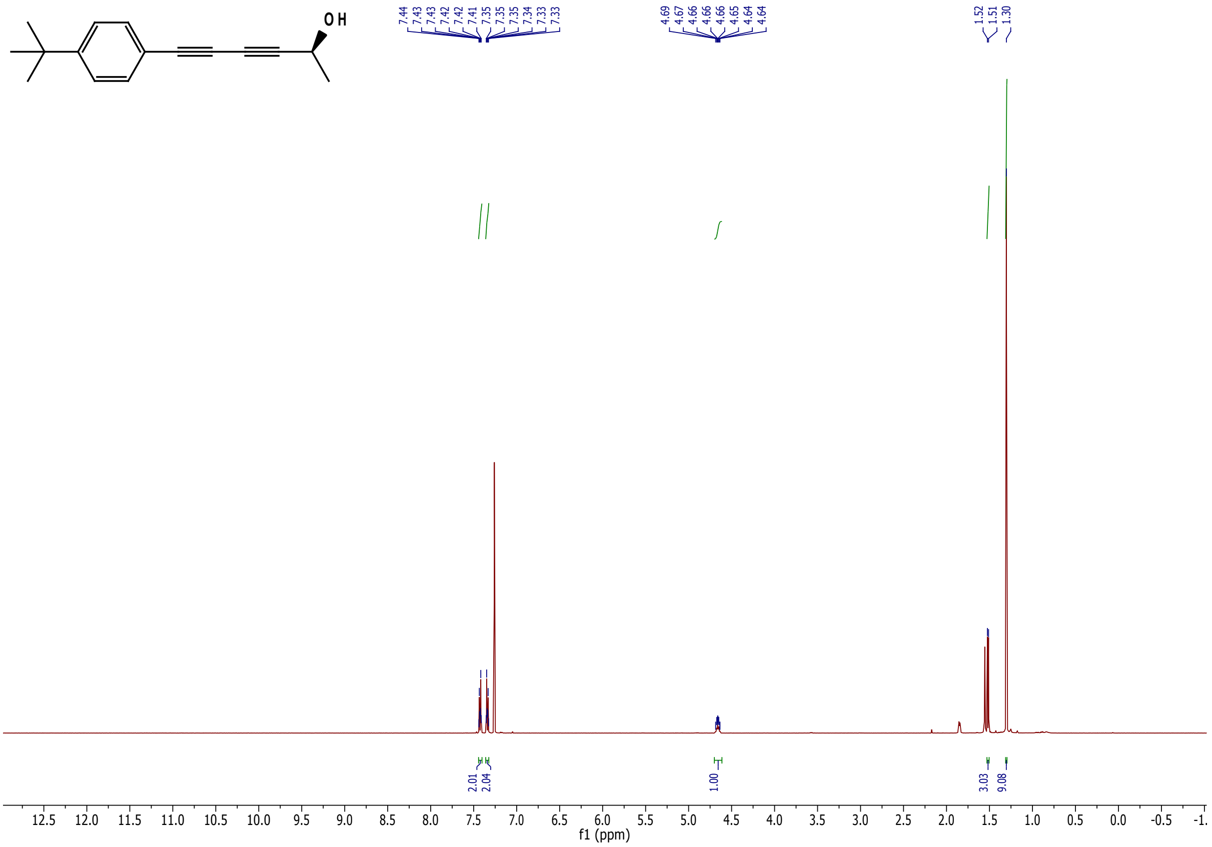

^{1}H NMR spectrum (500.1 MHz, $CDCl_3$) of (*S*)-6-(4-(*Tert*-butyl)phenyl)hexa-3,5-diyn-2-ol ((*S*)-**21**)

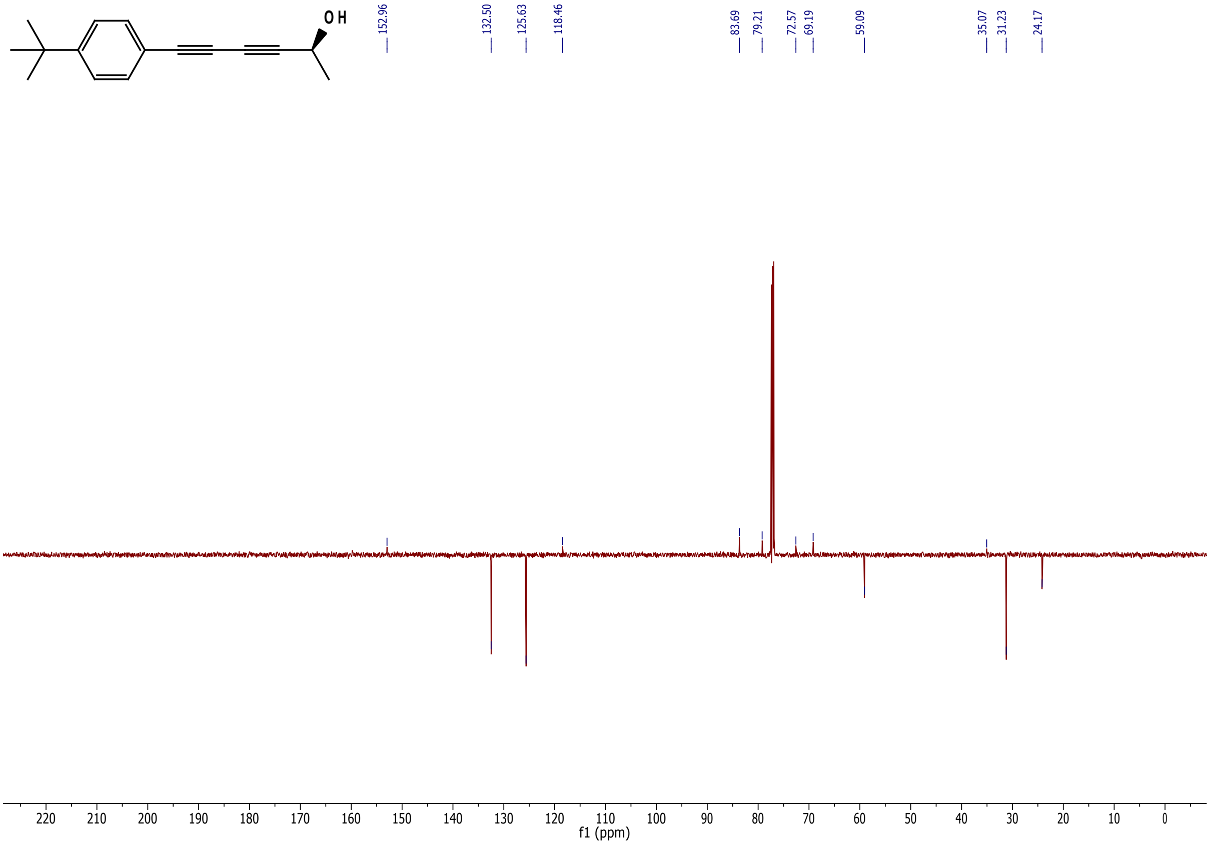

^{13}C NMR spectrum (126 MHz, $CDCl_3$) of (*S*)-6-(4-(*Tert*-butyl)phenyl)hexa-3,5-diyn-2-ol ((*S*)-**21**)

Supporting Figures for receptor assays and computational biology

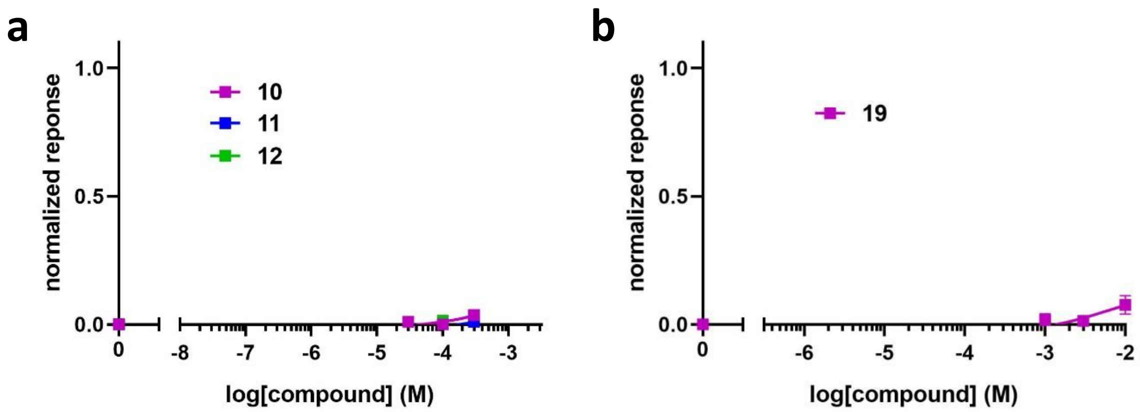

Figure S4. Dose-response curves of the response of OR1A1 to a) the mono-acetylenes **10**-**12** and b) the *tris*-acetylene **19**.

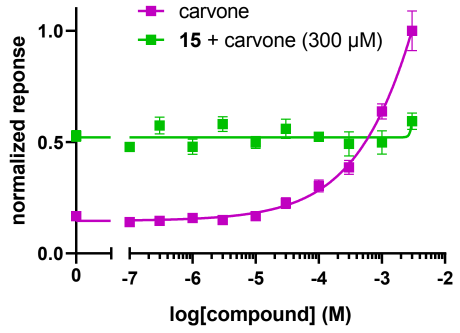

Figure S5. The absence of antagonistic activity of Compound 15. When increasing concentrations of **15** was added to OR1A1 activated by 300 µM of carvone, no change in OR1A1 activity was observed. Normalized dose-response to carvone is shown as a reference.

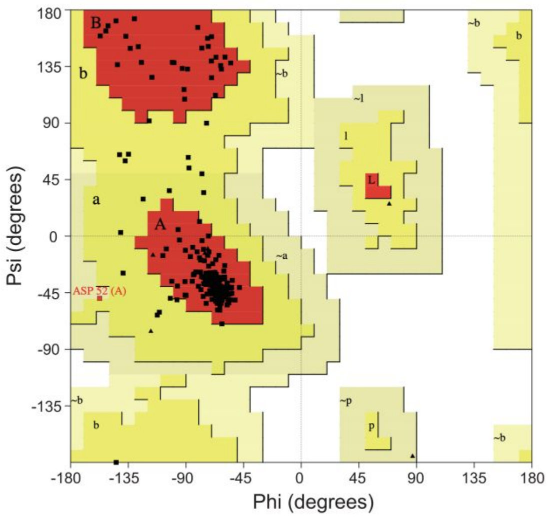

Figure S6. Ramachandran plot of OR1A1. The red regions represent the most favourable dihedral angle positions, the yellow regions represent allowed positions, and the pale-yellow regions represent marginally allowed positions.

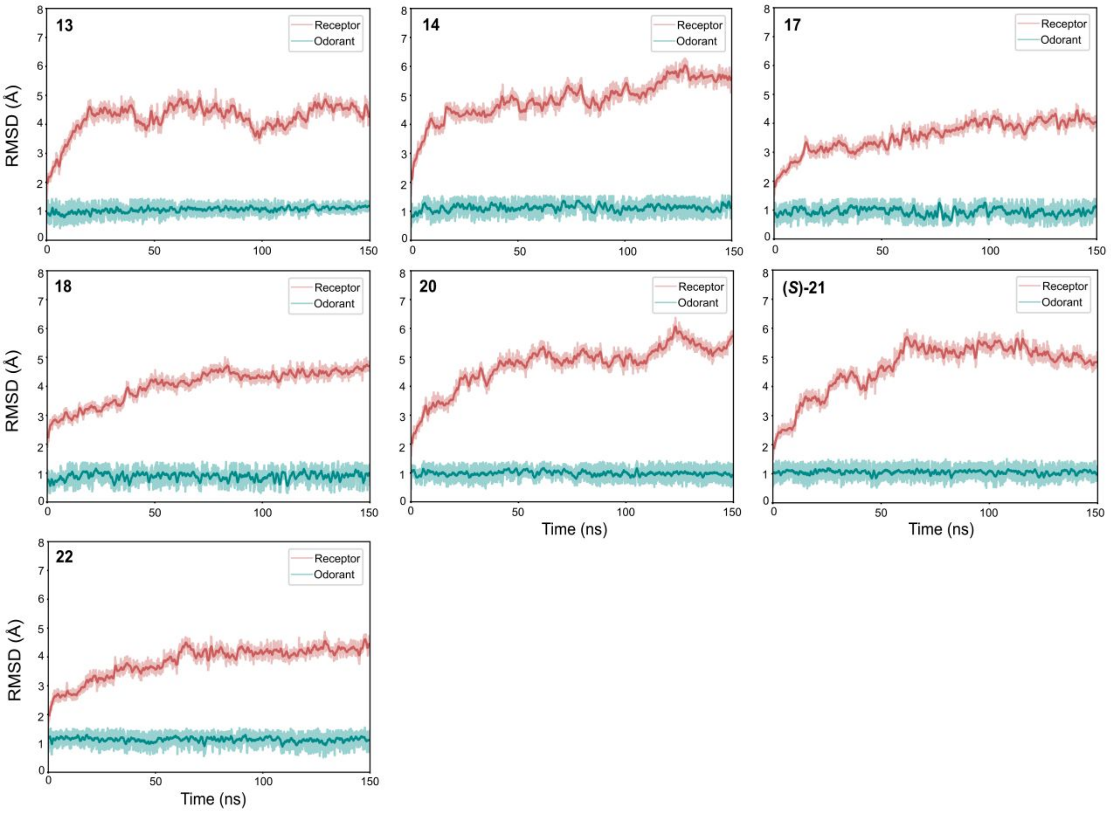

Figure S7. RMSD profiles from the MD simulations of OR1A1 with compounds 13, 14, 17, 18, 20, (*S*)-21, and 22. Three parallel simulations were conducted for each odorant to ensure statistical robustness. The average RMSD of the three simulations is shown. The RMSD of the receptor is depicted in red while the RMSD of the odorant is depicted in cyan.

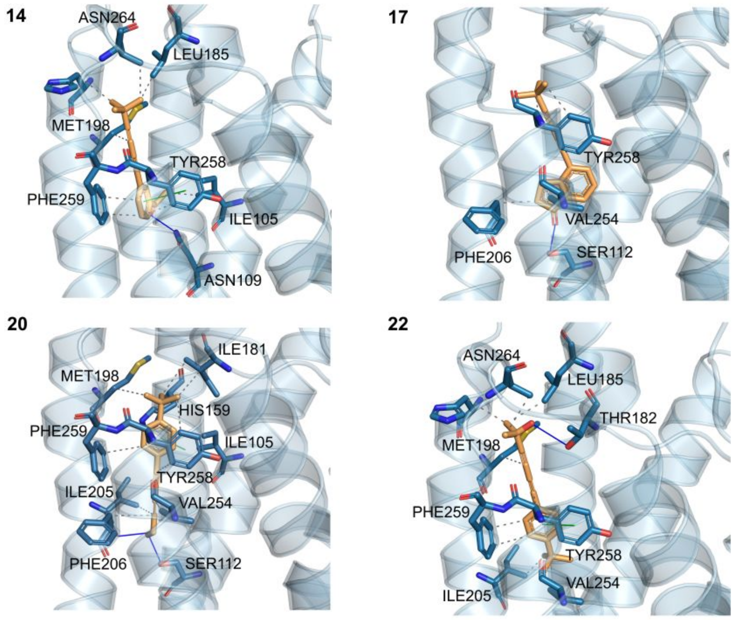

Figure S8. Binding modes of ligands with OR1A1. Each panel represents a different ligand, highlighting the interactions between the odorant (orange) and residues (blue) within the binding pocket of OR1A1, where gray dashed lines represent hydrophobic interactions, green solid lines represent π-π stacking, and blue solid lines represent hydrogen bonds.

References

1 H. Wu, S. Chen, D. Xiao, F. Li, K. Zhou, X. Yin, C. Liu, X. He and Y. Shang, 'Visible-light-mediated deacylated alkynylation of unstrained ketone**.'** *Org. Lett.*, **2023**, *25*, 1166–1171.

2 K. Ni, L.-G. Meng, H. Ruan and L. Wang, 'Controllable chemoselectivity in the coupling of bromoalkynes with alcohols under visible-light irradiation without additives: synthesis of propargyl alcohols and α-ketoesters**.'** *Chem. Commun.*, **2019**, *55*, 8438–8441.

3 K. Nicholson, J. Dunne, P. DaBell, A. B. Garcia, A. D. Bage, J. H. Docherty, T. A. Hunt, T. Langer and S. P. Thomas, 'A boron–oxygen transborylation strategy for a catalytic Midland reduction.' *ACS Catal.*, **2021**, *11*, 2034–2040.

4 K. Yasui, N. Chatani and M. Tobisu, 'Rhodium-catalyzed C-O bond alkynylation of aryl carbamates with propargyl alcohols.' *Org. Lett.*, **2018**, *20*, 2108–2111.

5 C. P. Tüllmann, Y.-H. Chen, R. J. Schuster and P. Knochel, 'Preparation and reactions of mono- and bis-pivaloyloxyzinc acetylides.' *Org. Lett.*, **2018**, *20*, 4601–4605.

6 K. Osowska, T. Lis and S. Szafert, 'Protection/deprotection-free syntheses and structural analysis of (keto-aryl)diynes.' *Eur. J. Org. Chem.*, **2008**, *2008*, 4598–4606.

7 A. Vlasceanu, C. L. Andersen, C. R. Parker, O. Hammerich, T. J. Morsing, M. Jevric, S. Lindbæk Broman, A. Kadziola and M. B. Nielsen, 'Multistate switches: Ruthenium alkynyl–dihydroazulene/vinylheptafulvene conjugates**.'** *Chem. Eur. J.*, **2016**, *22*, 7514–7523.

8 Y.-J. Li, D.-G. Liu, J.-H. Ren, T.-J. Gong and Y. Fu, 'Photocatalytic alkyl radical addition tandem oxidation of alkenyl borates.' *J. Org. Chem.*, **2023**, *88*, 4325–4333.

9 G. C. Tsui, K. Villeneuve, E. Carlson and W. Tam, 'Ruthenium-catalyzed [2 + 2] cycloadditions between norbornene and propargylic alcohols or their derivatives.' *Organometallics*, **2014**, *33*, 3847–3856.

10 R. Plamont, L. V. Graux and H. Clavier, 'Highly selective syn addition of 1,3-diones to internal ynamides catalyzed by zinc iodide.' *Eur. J. Org. Chem.*, **2018**, *2018*, 1372–1376.

11 F. H. Lutter and M. Jouffroy, ‘Facile conversion of molecularly complex (hetero)aryl carboxylic acids into alkynes for accelerated SAR exploration.’ *Chem. Eur. J.*, **2021**, *27*, 14816–14820.

12 A. C. A. D’Hollander and N. J. Westwood, ‘Assessment of the regioselectivity in the condensation reaction of unsymmetrical o-phthaldialdehydes with alanine.’ *Tetrahedron*, **2018**, *74*, 224–239.

13 Y. Gu, S. N. Natoli, Z. Liu, D. S. Clark and J. F. Hartwig, ‘Site-selective functionalization of (sp3)C−H bonds catalyzed by artificial metalloenzymes containing an iridium-porphyrin co-factor.’ *Angew. Chem. Intl. Ed.*, **2019**, *58*, 13954–13960.

14 J. E. D. Kirkham, T. D. L. Courtney, V. Lee and J. E. Baldwin, ‘Asymmetric synthesis of cytotoxic sponge metabolites R-strongylodiols A and B and an analogue.’ *Tetrahedron*, **2005**, *61*, 7219–7232.

15 B. M. Trost, V. S. Chan and D. Yamamoto, ‘Enantioselective ProPhenol-catalyzed addition of 1,3-diynes to aldehydes to generate synthetically versatile building blocks and diyne natural products.’ *J. Am. Chem. Soc.*, **2010**, *132*, 5186–5192.
